# Synthesis of Phosphonate Derivatives of Benzisoselenazolones and Their Remarkable Antiureolytic Activity in *Helicobacter pylori* Cells

**DOI:** 10.1021/acs.jmedchem.5c01385

**Published:** 2025-09-30

**Authors:** Marta Grabarek, Wojciech Tabor, Paweł Krzyżek, Julia Bąkowicz, Agnieszka Grabowiecka, Łukasz Berlicki, Artur Mucha

**Affiliations:** † Department of Bioorganic Chemistry, Faculty of Chemistry, Wrocław University of Science and Technology, Wybrzeże Wyspiańskiego 27, 50-370 Wrocław, Poland; ‡ Department of Microbiology, Faculty of Medicine, Wrocław Medical University, Chałubińskiego 4, 50-368 Wrocław, Poland; § Institute of Advanced Materials, Faculty of Chemistry, Wrocław University of Science and Technology, Wybrzeże Wyspiańskiego 27, 50-370 Wrocław, Poland

## Abstract

The attachment of a nickel-ion-complexing functionality to the structures of covalent inhibitors of ureases has been considered an effective method for enhancing binding to these pivotal virulence factors of various microbial pathogens. Following this approach, we envisioned a structural combination of 1,2-benzisoselenazol-3­(2*H*)-one, a scaffold that produced the most significant antiureolytic effect achieved, with a phosphonic acid group intended to block the function of nickel ions in the catalytic mechanism. The multistep preparation of hybrid compounds involved aminolysis of 2-(chloroseleno)­benzoyl chloride with the key diethyl aminophosphonate intermediates, followed by hydrolysis of the final phosphonate esters. Although not entirely consistent with the rationale of the design idea, the esters themselves, rather than the corresponding acids, demonstrated more substantial inactivation of the model *Sporosarcina pasteurii* urease and inhibition of ureolysis in *Helicobacter pylori*. In particular, IC_50_ values in pathogen cells reached an unprecedented range of 30–40 nM for some compounds.

## Graphical abstract

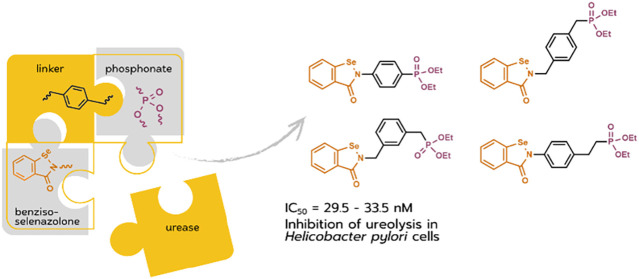

## Introduction

Covalent inhibition based on the specific reactivity of functionalized low-molecular-weight organic compounds has recently emerged as a promising approach to effectively control the activity of bacterial ureases. Urease,^1^ a landmark enzyme in advances in biochemistry and medicine,^2,3^ is a hydrolase that catalyzes amide bond cleavage in urea to form ammonia and carbamate; then, carbamate hydrolyzes spontaneously. The general process of decomposing urea to ammonia and carbon dioxide that occurs in plants, fungi, and bacteria is an important element of the global circulation of nitrogen.^4^ The anthropogenic aspect of this system mainly reflects the issue of nitrogen fertilizers and the limitation of their efficacy due to hydrolysis by macrobiotic ureases in the soil. Nitrogen losses require extensive use of urea, while the overproduction of ammonia as a direct result of uncontrolled ureolysis in the environment leads to water and air pollution and water eutrophication.^5,6^ With respect to human health, the importance of bacterial ureolysis lies in the medical potential of its control as a virulence factor of persistent pathogenic microorganisms, such as *Proteus mirabilis* or *Helicobacter pylori*, which infect the urinary and gastrointestinal tracts, respectively.^7^ Products of ureolytic activity are involved in the formation of urinary kidney stones^8^ or allow bacteria to survive in the acidic environment during colonization and induce chronic gastritis and peptic and duodenal ulceration, with the risk of gastric cancers.^9^ Inhibition of urease activity reduces the virulence, colonization abilities, and viability of pathogens and increases their susceptibility to first-line antibiotics.^7,10,11^

In light of the growing interest in covalent drugs over the last two decades,^12–15^ particularly those reacting with the cysteine residue,^16^ urease has been viewed as an excellent antibacterial target, susceptible to inactivation based on selective reactivity. Three categories of compounds, namely, catechols, Michael acceptors, and organoselenium derivatives, have been successfully utilized for this purpose.^17–19^ Research on catechols as potential inhibitors of bacterial ureases was inspired by previous findings related to the inhibition of ureolysis in soil^20^ and in plants (jack bean)^21^ that involved natural polyphenolic structures. Accordingly, catechins, secondary antioxidant flavonoid metabolites found in green tea extracts, were shown to inactivate *H. pylori* urease (e.g., (−)-epigallocatechin gallate, IC_50_ = 2.2 μM).^22^ Subsequently, various activities (typical IC_50_ values at moderate micromolar concentrations) were measured for other flavonoids isolated from natural sources and/or synthetically modified.^23–25^ Parallel SAR studies of a series of synthetic isoflavone-based pyrogallol and catechol derivatives identified the 1,2-diphenylethane core and two *ortho* hydroxyl groups as essential structural/functional elements for their activity against *H. pylori* urease.^26,27^ Further optimization of this scaffold yielded derivatives with increased affinity, such as 4-((4-nitrophenylamino)­methyl)­benzene-1,2-diol (IC_50_ = 0.62 μM for cell-free *H. pylori* urease; IC_50_ = 1.92 μM for ureolysis in intact cells; Figure 1, panel A).^28,29^ Pagoni et al. identified propargyl 3,4-dihydroxyphenylacetate, an active, covalent, and irreversible inhibitor of *Sporosarcina pasteurii* urease (IC_50_ = 0.52 μM; Figure 1, panel A), among 40 amide and ester derivatives of 3,4-dihydroxyphenylacetic acid, caffeic acid, ferulic acid, and gallic acid.^30^ In the most recent studies, to enhance noncovalent interactions with catalytic nickel ions and surrounding basic amino acid residues, catechol reactivity was combined with the presence of phosphonic/phosphinic and carboxylate moieties.^31,32^ The products were shown to be effective ligands and demonstrated complex binding kinetics. For some of these, such as 1-(3,4-dihydroxyphenyl)-2-methoxycarbonylethylphosphonic acid, the inactivation process was slow but reversible (*K*_i_ = 0.32 μM, dissociation constant of the initial enzyme–inhibitor complex; and *K*_i_* = 0.13 μM, overall dissociation constant; Figure 1, panel A, frame), while others, like 1-(3,4-dihydroxyphenyl)-2-methoxycarbonylethyl­(2-carboxyethyl)­phosphinic acid, were typically irreversible (*k*_inact_/*K*_I_ = 10,420 s^–1^ M^–1^; Figure 1, panel A, frame).^31^ The specific mechanism of ureolysis inhibition contributed to the safety and nontoxicity of catechol-based phosphonic and phosphinic acids against mammalian cells.^32^ Ciurli et al. provided fundamental kinetic, computational, and structural data supporting the inactivation mechanism of ureases by catechols.^33,34^ The authors proposed a multistep radical process leading to the substitution of the aromatic ring with the reactive thiol group of the cysteine residue to create the covalent product (Figure 1, panel A). The key cysteine residue, for instance, Cys322 in *S. pasteurii* and Cys321 in *H. pylori* (numbering of the α subunit of (αβγ)_3_ or ((αβ)_3_)_4_ quaternary arrangement,^33,35^ respectively), is located within the loop of the movable helix–loop–helix flap that changes its conformation to accommodate the substrate for hydrolysis.^36^ Covalent modifications hinder the access of urea to the active site and disrupt the adoption of the closed conformation necessary for catalysis.^37^

**Figure 1:**
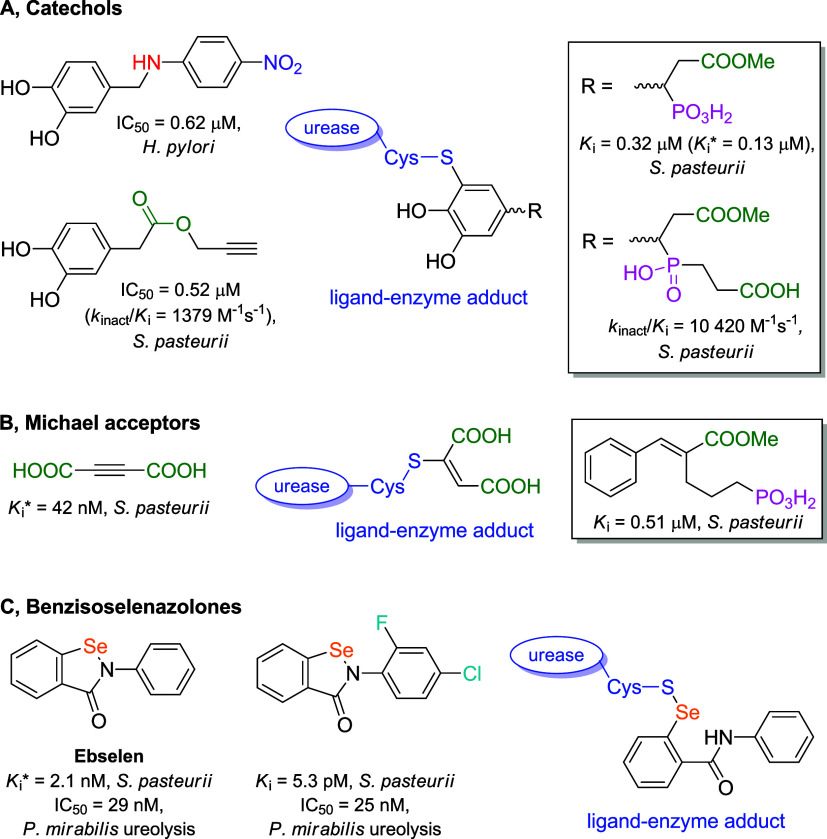
Background of the study. Structures of representative examples of covalent inhibitors of bacterial ureases, their activities against isolated enzymes (indicated by the systematic name of the microorganism) or ureolysis performed by pathogenic live cells, and proposed binding modes (ligand-enzyme adducts): catechol derivatives (panel A), Michael acceptors (panel B), and 1,2-benzisoselenazol-3­(2*H*)-ones (panel C). Modifications containing a phosphorus atom are shown within the frames (for benzisoselenazol-3­(2*H*)-ones, the focus of the current work).

The second group of covalent inhibitors of ureases consists of α,β-unsaturated compounds that undergo nucleophilic addition with the sulfhydryl group of the key cysteine residue (Michael addition). This activity was first demonstrated to retard the hydrolysis of urea in soil with *p*-benzoquinone.^38^ Regarding bacterial enzymes, differently substituted benzo- and naphthoquinones noncompetitively and irreversibly inhibited *S. pasteurii* urease and ureolysis in the whole cells of *H. pylori*, *Klebsiella oxytoca*, and *P. mirabilis*.^39^ *p*-Benzoquinone was also confirmed as the most active among 70 commercially available compounds screened against the ureolytic bacterium *Klebsiella pneumoniae*.^40^ However, the application of quinones is not beneficial, as it is associated with low selectivity issues that result in cytotoxicity.^41^ Macegoniuk et al. identified potent inactivators of *S. pasteurii* urease among a series of 40 α,β-unsaturated carbonyl and carboxyl compounds.^42^ Low nanomolar values of steady-state inhibition constants (slow-binding mechanism of action, *K*_i_* ∼ 10 nM) were calculated for methyl and ethyl esters of acetylenedicarboxylic acid. The acid itself was somewhat less potent (*K*_i_* = 42 nM; Figure 1, panel B) but highly specific, as it demonstrated low chemical reactivity with alternative thiols and no cytotoxicity with mammalian cells. Its modeled binding mode revealed simultaneous addition with the Cys322 sulfhydryl group and coordination of a carboxylate with nickel ions of the active site. To further explore this cooperativeness, we combined the reactive α,β-unsaturated structure of cinnamate with phosphonic/phosphinic functionalities that were expected to enhance the complexation abilities of the products.^43,44^ As a result, a slow-binding submicromolar inhibitor of *S. pasteurii* urease (*K*_i_ = 0.51 μM; Figure 1, panel B, frame) was identified; it also affected the whole cell-mediated ureolysis of *P. mirabilis* at the micromolar level (IC_50_ = 11 μM, with a 2 h preincubation time).

2-Substituted 1,2-benzisoselenazol-3­(2*H*)-ones, derivatives of Ebselen—an antioxidant, anti-inflammatory, cytoprotective organoselenium compound that reacts with thiols,^45,46^ represent the most recently developed and powerful group of covalent inhibitors of bacterial ureases. Ebselen (2-phenyl-1,2-benzisoselenazol-3­(2*H*)-one) was initially characterized as a potent competitive binder of the urease of *S. pasteurii* (*K*_i_* = 2.1 nM; Figure 1, panel C).^47^ Substitution of the 2-phenyl ring, particularly dihalogenation, yielded derivatives that demonstrated an impressive three orders of magnitude improvement in affinity compared to the lead structure (e.g., 2-(4-chloro-2-fluorophenyl)-1,2-benzisoselenazol-3­(2*H*)-one, *K*_i_ = 5.3 pM; Figure 1, panel C).^48^ The mentioned compounds effectively controlled ureolysis in live pathogenic *P. mirabilis* cells at remarkably low concentrations (IC_50_ = 29 nM for Ebselen and IC_50_ = 25 nM for 4-chloro-2-fluoro modification). However, this effect was not observed in *H. pylori* cells. Recently, we published data on halogenated 2-benzyl benzisoselenazolones.^49^ These compounds altered the ureolytic activity of live *H. pylori* at IC_50_ < 100 nM, as reported for the first time. Other modified organoselenium derivatives of this framework, namely, 2-aryloyl benzisoselenazolones and benzoselenazolyl benzoates, were currently targeted against ureolysis performed by *H. pylori*.^50^ The best newly synthesized covalent inhibitor exhibited an IC_50_ = 0.14 μM against the free enzyme and MIC = 8 μg/mL with pathogenic cells. The commonly accepted mechanism of action of Ebselen and its derivatives on proteins involves the cleavage of the Se–N bond in the benzisoselenazolone ring by a nucleophilic thiol/thiolate of the cysteine residue, forming a new S–Se bond (Figure 1, panel C).^51^ The reaction is rapid and efficient, selective against other nucleophiles, and can be reversed by adding low-molecular-weight thiols. The formation of the enzyme–inhibitor complex is typically facilitated by extensive hydrophobic interactions with the protein surface.^52^ This mode of action was also proposed for the inhibition of urease by Ebselen and was supported by molecular modeling studies.^47^ However, the initial complex may undergo aging due to its S_N_Ar-like nucleophilic hydrolysis, releasing salicylanilide and the selenated enzyme as recently observed for the inactivation of M^pro^, the main protease of SARS-CoV-2,^53^ and then *S. pasteurii* urease.^48^

In the current work, we envisaged using the 1,2-benzisoselenazol-3­(2*H*)-one structure for synthesizing hybrid compounds modified with a phosphonic acid functionality at the terminus of 2-substituents. To classify the results in the context of previous data, we first studied these compounds with the model bacterial urease of *S. pasteurii*; however, efficient inhibition of ureolysis in whole *H. pylori* cells remained the most challenging goal.

## Results and Discussion

### Compounds

Among the three discussed groups of covalent inhibitors of bacterial ureases (catechols, Michael acceptors, and organoselenium derivatives), benzisoselenazolones, although not antibiotics as such, appear to exhibit the most significant potential for developing effective antimicrobial therapies through a repurposing approach.^54^ The anti-inflammatory, antioxidant, and cytoprotective activities of Ebselen, the prototypical representative, are well characterized;^45,46^ the same holds for its toxicity and pharmacological properties.^55^ On the other hand, the fundamental activity of this compound in bacteria is specific and involves inhibition of thioredoxin reductase, which alters the reduction of disulfides in multiple substrates, influences cell redox status, impacts the biosynthesis of DNA and cellular proteins, and induces oxidative stress.^56^ Beneficially, these effects can be enhanced by inactivating bacterial virulence factors, such as urease.^57^ Employing these principles, we proposed functionalizing the advantageous 1,2-benzisoselenazol-3­(2*H*)-one structure with a phosphonic acid functionality (Figure 2). This group was intended to be located at the 2-substituent to facilitate the formation of additional ligand–protein interactions, particularly the complexation of catalytic nickel ions. Noncovalent blocking of the catalytic function of the metal ions by phosphorus-based acid has been proven to be one of the most effective approaches to inhibit microbial ureases.^58–60^ As mentioned above, the corresponding functionalization of catechols and Michael acceptors resulted in covalent enzyme inactivators with improved characteristics. To perform these modifications, a series of 2-substituents was utilized: alkyl, aryl, or arylalkyl (compounds **1a**–**l**; Figure 2); their length and regioisomerism varied widely to bridge the gap between the target sites in the enzyme structure: the cysteine residue in the movable flap and the central nickel ions.

**Figure 2:**
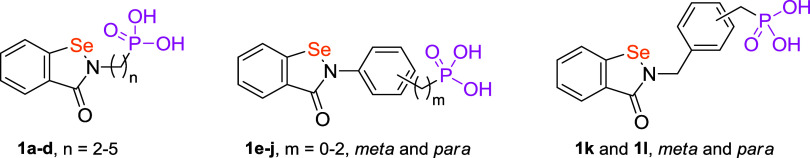
Designed structures: phosphonic acid-modified 2-alkyl, aryl, or arylalkyl 1,2-benzisoselenazol-3­(2*H*)-ones that contain a selenium heterocycle reactive with cysteine thiolate located within the movable flap at the entrance to the active site of urease, along with a phosphonic group intended for forming noncovalent ligand–protein interactions, particularly complexation with nickel ions.

### Synthesis and Structure

The synthesis of the target compounds was based on the aminolysis of 2-(chloroseleno)­benzoyl chloride with aminophosphonates **5a**–**l** of the designed structures (Scheme 1). To achieve these key aminophosphonate scaffolds, the phosphonate group was introduced to suitable alkyl or aryl halide substrates using classical approaches, namely, the Michaelis–Arbuzov reaction^61^ or palladium-catalyzed cross coupling,^62^ respectively. Amino functionalization, in turn, involved Gabriel synthesis (for the alkyl amino group)^63^ or reduction of the nitro group (for the aryl amino group),^64^ as outlined in panels A–D. Specifically, diethyl ω-aminoalkylphosphonates **5a**–**d** were obtained from α,ω-dibromoalkanes **2a**–**d** in a three-step synthetic route via 2-(ω-bromoalkyl)­phthalimides **3a**–**d** and the corresponding diethyl ω-phthalimidoalkylphosphonates **4a**–**d** through two consecutive substitutions with potassium phthalimide and triethyl phosphite, followed by hydrazinolysis of imides **4a**–**d** (Scheme 1, panel A). The Arbuzov reaction step was aided by microwave induction,^65^ which allowed for a short heating time and resulted in excellent yields. As chromatography purification of **5a**–**d** did not enhance the quality of the final materials (due to partial ester hydrolysis), the crude products after hydrazinolysis were used in the subsequent aminolysis of chloride **13**.

**Scheme 1:**
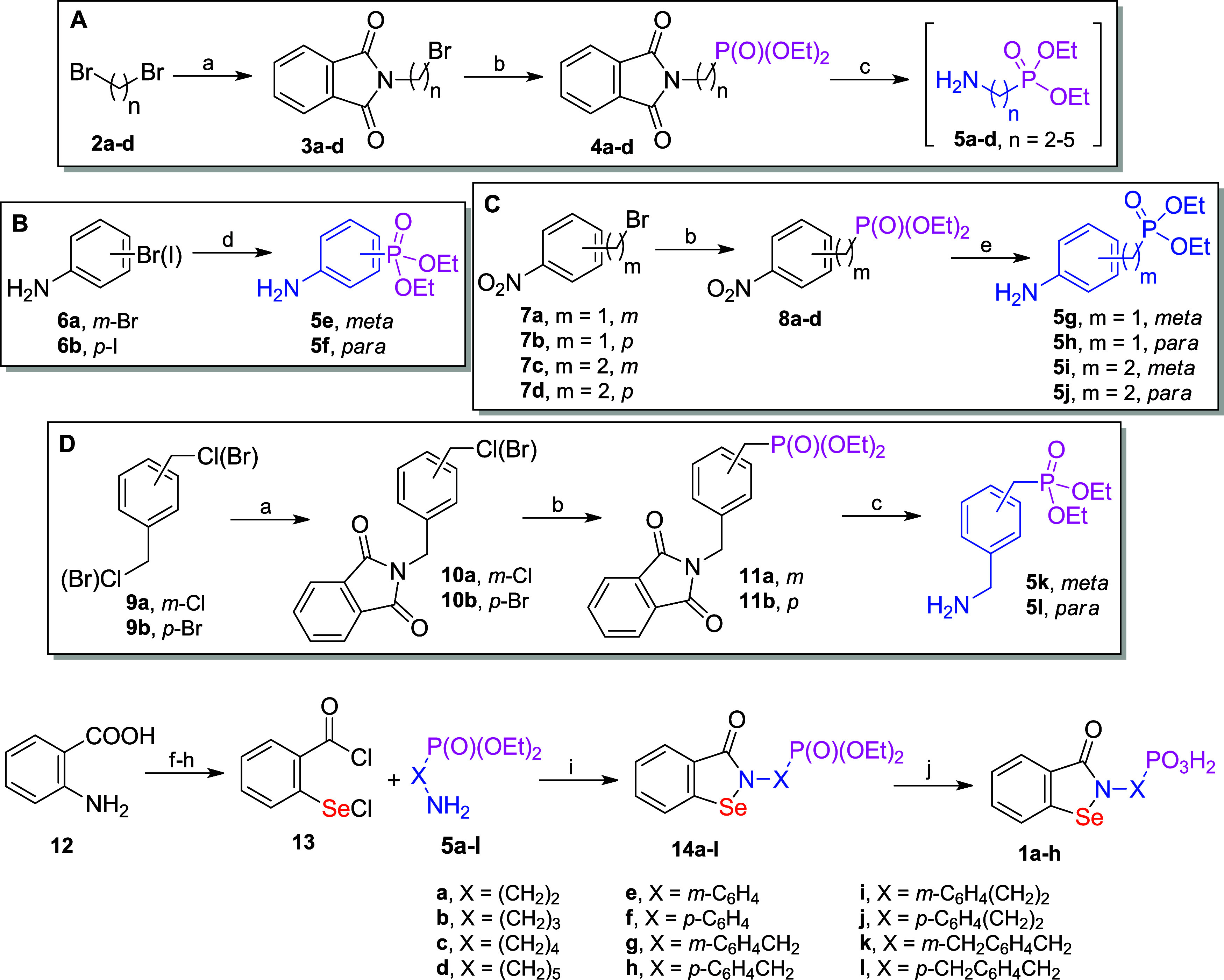
Multistep Synthesis of Diethyl Phosphonate and Phosphonic Acid Derivatives of 2-Substituted Benzisoselenazolones, Including the Preparation of Key Intermediate Aminophosphonates (Panels A–D, Frames)^a^

a Reagents and conditions: (a) potassium phthalimide, DMF, 100 °C, 8 h, 93% (**3a**), 92% (**3b**), 90% (**3c**), 89% (**3d**), 65% (**10a**), 65% (**10b**); (b) P­(OEt)_3_, MW, 140 °C, 2 h, 96% (**4a**), 96% (**4b**), 89% (4c), 95% (**4d**), 91% (**8a**), 84% (**8b**), 73% (**8c**), 71% (**8d**), 66% (**11a**), 69% (**11b**); (c) N_2_H_4_, EtOH, rt, 12 h, not separated (**5a–d**), 77% (**5k**), 81% (**5l**); (d) HPO­(OEt)_2_, Pd­(OAc)_2_, Ph_3_P, Et_3_N, EtOH, reflux, Ar, 24 h, 80% (**5e**) or P­(OEt)_3_, PdCl_2_, TBAB, *n*-Pr_3_N, H_2_O, reflux, 3 h, 53% (**5f**); (e) SnCl_2_, EtOH, HCl, reflux, Ar, 2h, 82% (**5g**), 87% (**5h**), 76% (**5i**), 72% (**5j**); (f) NaNO_2_, 10% HCl_aq_, −5 °C; (g) Na_2_Se_2_, H_2_O, −20 to −10 °C, then rt, 48 h; (h) SOCl_2_, DMF, reflux, 2 h, 44% (13, steps f–h, totally); (i) Et_3_N, CH2Cl_2_, rt, 52–81% (**14a–l**); and (j) 35–37% HCl_aq_/CH_3_COOH (1:1, v/v), MW, 140 °C, 20 min, 48–70% (**1a–h**).

To obtain diethyl *meta*- and *para*-aminophenylphosphonates (**5e** and **5f**, Scheme 1, panel B), two alternative conditions for palladium-catalyzed coupling with a phosphorus component were compared. The Hirao reaction, starting with *m*-bromoaniline (**6a**) and diethyl phosphite,^66^ proved to be more effective than the coupling of iodoaniline (**6b**) and triethyl phosphite under aqueous conditions.^67^ Diethyl *meta*- and *para*-aminobenzyl- and aminophenetylphosphonates (**5g**–**j**) were synthesized from nitrophenylalkyl bromides **7a**–**d** in the Arbuzov reaction, followed by the reduction of diethyl nitrophenylalkylphosphonates **8a**–**d** with tin­(II) chloride (Scheme 1, panel C). Finally, diethyl *meta*- and *para*-(aminomethyl)­benzylphosphonates (**5k** and **5l**) were obtained from xylylene halides (**9a** and **9b**) via (phthalimidomethyl)­benzyl halides (**10a** and **10b**) and (phthalimidomethyl)­benzylphosphonates (**11a** and **11b**), combining the analogous transformations described above for diethyl alkylphosphonates **5a**–**d** (Scheme 1, panel D). Several aminophosphonate compounds mentioned have been reported in the literature.^66–72^ Nevertheless, the preparation details, along with a comprehensive characterization of all structures (except aminoesters **5a**–**d**, used as crude) and their precursors, both known and previously unreported, are provided in the Supporting Information.

The resulting diethyl aminophosphonates **5a**–**l** were subjected to reactions with 2-(chloroseleno)­benzoyl chloride (**13**), the latter obtained from anthranilic acid (**12**), carried out according to the standard procedure.^73,74^ The entire series of final diethyl phosphonate derivatives of 2-substituted benzisoselenazolones **14a**–**l** was separated with good yields after workup and chromatographic purification. Subsequently, several conditions for acidic hydrolysis or dealkylation (TMSBr) of phosphonate esters were tested to ensure practical yields of free phosphonic acids. A mixture of acetic acid and concentrated hydrochloric acid, heated briefly in a microwave oven at 140 °C, was found to be the most convenient approach. This method avoided extensive isoselenazolone ring opening, the main unwanted acid-mediated side reaction. After chromatographic purification, some target products yielded well-defined crystals (Figure 3). Compound **1a** crystallized in the triclinic space group *P*1̅ with three symmetrically independent molecules, A, B, and C, in the asymmetric unit (only one of them, molecule A, is presented in Figure 3, panel A), while compound **1c** (Figure 3, panel B) crystallized in the monoclinic space group *P*2_1_/*n*.

**Figure 3:**
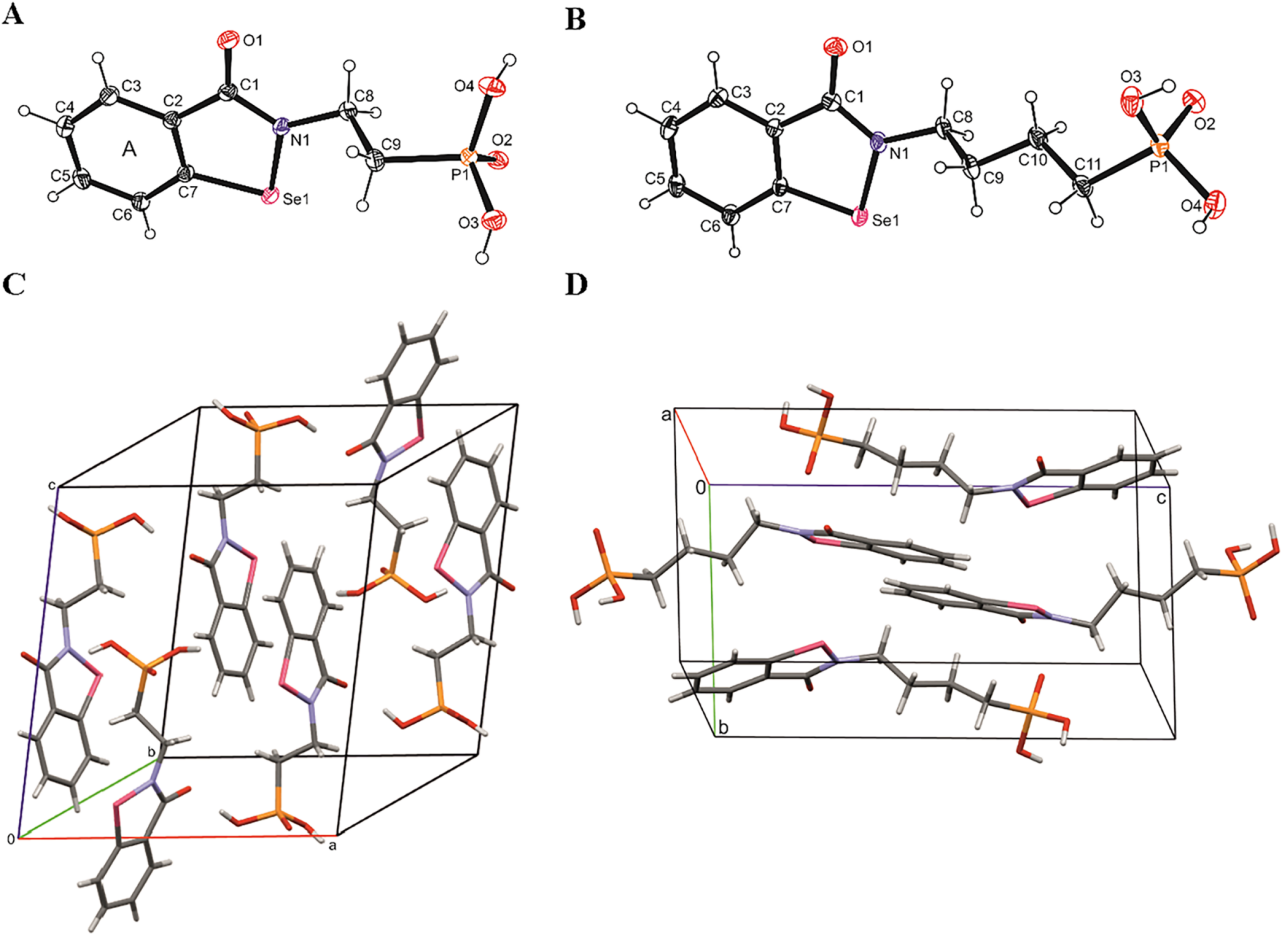
Molecular structure of phosphonic acids **1a** and **1c** with the atom-numbering scheme (panels A and B).^75^ Displacement ellipsoids are drawn at the 25% probability level, and H atoms are represented as small spheres with arbitrary radii. The arrangement of the molecules in the unit cells was prepared using the Mercury program (panels C and D).^76^ Crystallographic data for the structures have been deposited with the Cambridge Crystallographic Data Centre as supplementary publication nos. CCDC 2323717 and CCDC 2323716.

Regarding the supramolecular arrangement, the structures of compounds **1a** and **1c** revealed somewhat different organizations and modes of interaction (Figure 3, panels C and D, respectively). In fact, in both cases, all three oxygen atoms of the phosphonic acid group were involved in hydrogen contacts; two of them included the adjacent acid functionality­(ies), while the third one pointed toward the oxo substituent of the neighboring isoselenazole ring. However, for the structure of **1a**, the contact between the phosphonic acid groups was bidentate and formed a dimeric arrangement, while for the structure of **1c**, the interactions involved the groups located above and below, linking a stack of molecules perpendicularly to their planar surface. The hydrophobic benzisoselenazolone fragments of **1a** molecules, alternately inverted in opposite directions, formed π-stacking piles. In the layers of **1c**, nonpolar contacts involved an interchangeable superposition of the alkyl on the aryl part of the molecules.

We decided against performing hydrolysis for all members of the compound set. When tested for inhibition of ureolysis, somewhat surprisingly and contrary to design principles, the acids appeared significantly less active than their esterified counterparts, in several instances even by an order of magnitude (*vide infra*). Therefore, we deemed it unnecessary to remove the ethyl groups from the last four compounds, **14i**–**l**, of the series. Instead, we explored the potential of selective hydrolysis. A single phosphonate alkyl residue could be cleaved under basic conditions (Scheme 2). Indeed, it performed satisfactorily, and two exemplified benzisoselenazolones were obtained and characterized: monoethyl ethylphosphonate **15a**, the analogue of **14a** and **1a**, and monoethyl pentylphosphonate **15b**, the analogue of **14d** and **1d**.

**Scheme 2:**
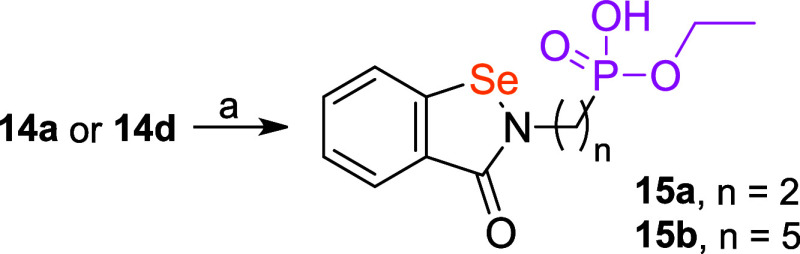
Selective Hydrolysis of Chosen Diethyl Phosphonates to Obtain Monoethyl Esters of Phosphonic Acids^a^

a Reagents and conditions: (a) 2 M NaOH_aq_, MeOH, rt, 2 h, 43% (1**5a**), 48% (**15b**).

### Antiureolytic Activity

The first group of compounds studied, 2-alkyl derivatives of 1,2-benzisoselenazol-3­(2*H*)-one containing diethyl phosphonate, phosphonic acid, or monoethyl phosphonate functionality at the ω position (**14a**–**d, 1a**–**d, 15a**, and **15b**, respectively), exhibited a quite unexpected structure–activity relationship. Phosphonate diesters **14a**–**d** were found to act as excellent inhibitors of purified urease from *S. pasteurii* (*K*_i_ = 12.8–71.6 nM) and of urea decomposition in live cells of *H. pylori* (IC_50_ = 130–299 nM; Table 1). The values of the kinetic parameters varied within a narrow range. For *S. pasteurii* urease, these changes were irregular with the length of the alkyl chain linker; nevertheless, the most extended compound (**14d**) was found to act as the most potent inhibitor. For *H. pylori* cells, the effectiveness of the ureolysis control decreased from the shortest homologue (**14a**) to the longest homologue (**14d**). To contextualize these data within the literature, a much more striking level of inhibition of *S. pasteurii* urease was previously reported with Ebselen^47^ and its derivatives,^48^ although the alteration of ureolytic activity in *H. pylori* described in this work reached nearly top levels.^49^

**Table 1:**
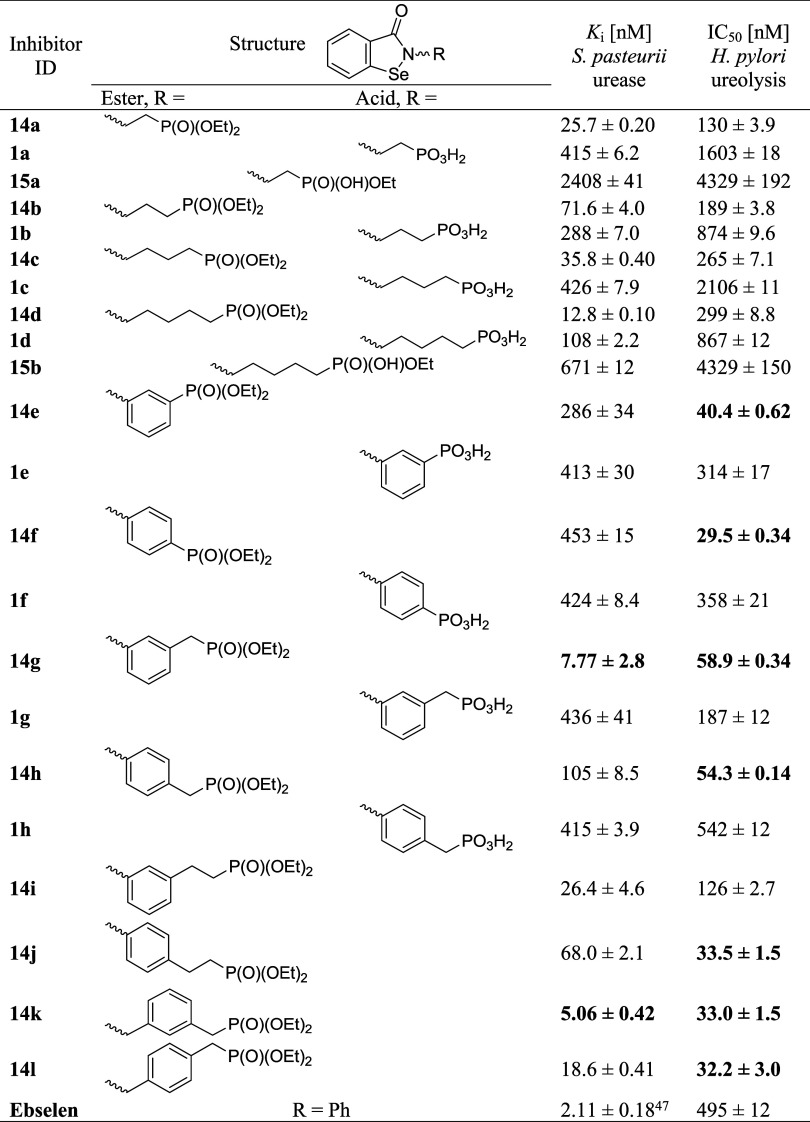
Inhibitory Activity of Synthesized Compounds against Urease of *S. pasteurii* and Ureolysis of Live Cells of *H. pylori* Tx30a (the Most Significant Data Are Indicated in Bold)

Surprisingly, phosphonic acids **1a**–**d** did not follow the remarkable characteristics of their parent diethyl esters **14a**–**d**. These acids were several times, up to an order of magnitude, weaker inhibitors in the two systems studied (*K*_i_ = 108–426 nM for *S. pasteurii* urease and IC_50_ = 867–2106 nM for *H. pylori* ureolysis, Table 1). In both assays, the most notable kinetic parameters were acquired for the 2-(5-phosphono)­pentyl analogue **1d**. Although objectively significant, the results suggested that the covalent binding of benzisoselenazolones was not facilitated by cooperative interactions of the phosphonic acid moiety. Partial hydrolysis of selected diethyl esters was also found to be ineffective with regard to potency. Monoethyl esters of phosphonic acids (**15a** and **15b**) demonstrated a further decrease in antiureolytic activity compared to the corresponding diethyl esters (**14a** and **14d**) and acids (**1a** and **1d**), as evidenced by micromolar values of measured kinetic constants (Table 1).

Studies of *meta*- and *para*-2-phenyl benzisoselenazolones, diethyl phosphonates **14e** and **14f**, and phosphonic acids **1e** and **1f** revealed a structure–activity relationship distinct from that discussed for 2-alkyl derivatives. The four compounds were practically equipotent toward *S. pasteurii* urease (*K*_i_ = 286–453 nM; Table 1), indicating good but decreased potency compared to compounds **14a**–**d**. For antiureolytic activity in *H. pylori*, the differentiation of phosphonate esters and phosphonic acid, by an order of magnitude of the IC_50_ value, became evident again. Furthermore, the control of urea decomposition by esters **14e** and **14f** in pathogen cells reached an unprecedented level, with IC_50_ at a nanomolar concentration: IC_50_ = 40.4 ± 0.62 nM for *meta* analogue **14e** and IC_50_ = 29.5 ± 0.34 nM for its *para* regioisomer **14f**. To the best of our knowledge, these IC_50_ values were lower than any other data in the literature reporting inhibition of ureolysis in native *H. pylori*.

The final structural combinations involved the installation of the diethyl phosphonate functionality at the termini of the 2-benzisoselenazolone substituents, which comprised alkyl and aryl fragments (*meta* and *para* regioisomers), such as benzyl (**14g** and **14h**), phenethyl (**14i** and **14j**), and xylyl (**14k** and **14l**). Addition of one flexible methylene linker prompted a visible improvement in inhibiting the activity of *S. pasteurii* urease by benzylphosphonates **14g** and **14h** compared to sterically rigid phenylphosphonates **14e** and **14f** (*K*_i_ = 7.77 ± 2.8 nM versus 286 ± 34 nM for *meta* homologues and *K*_i_ = 105 ± 8.5 nM versus 453 ± 15 nm for *para* homologues), while the potency of the products of the hydrolysis, phosphonic acids **1g** and **1h**, remained practically the same as that measured for **1e** and **1f**. In *H. pylori* cells, esters **14g** and **14h** exhibited excellent potency, characterized by IC50 values that are only twice that of **14f**. As the ester hydrolysis of compounds **14g** and **14h** did not enhance the activity of the resulting phosphonic acids **1g** and **1h**, we discarded this step for the remaining four structures and focused solely on phosphonates.

Further structural extension and increased flexibility resulted in the optimized potency of diethyl esters **14i**–**l**. These compounds demonstrated a high affinity for *S. pasteurii*, as indicated by low nanomolar *K*_i_ values (below 68 nM), with phosphonate **14k** being the most potent inhibitor identified in this study (*K*_i_ = 5.06 ± 0.42 nM). At the same time, three of them (**14j**–**l**) maintained the top antiureolytic effect in *H. pylori* cells (IC_50_ = 32.2–33.5 nM), comparable to that found for the 2-aryl derivative **14f**. In both systems studied, xylyl derivatives **14k** and **14l**, which possess two benzyl-type connections at the nitrogen atom of benzisoselenazolone and the phosphonate functionality, acted more efficiently than their structural isomers that comprised an ethylene linker (**14i** and **14j**). The reasons might be differences in the reactivity of the isoselenazolone system (*N*-benzyl versus *N*-aryl-substituted) or a potential to form noncovalent interactions with the enzymes (a lower steric rigidity of the xylyl system).

Finally, a certain regularity in the structure–activity relationship of phosphonate esters that include a phenyl fragment in their 2-substituent (**14e**–**l**) could be observed. Within each pair of compounds, the *meta* regioisomer exhibited a higher affinity in inhibiting *S. pasteurii* urease than its *para* counterpart (**14e** vs **14f**, **14g** vs **14h**, **14i** vs **14j**, and **14k** vs **14l**). Interestingly, the influence on ureolysis in *H. pylori* cells showed a consistently opposite behavior pattern. In most cases, the changes are subtle, and we would rather avoid further generalization.

### Binding Kinetics

Despite the structural and activity differences between diethyl phosphonates **14a**–**l** and phosphonic acids **1a**–**h**, all of these compounds demonstrated comparable binding kinetics to the urease of *S. pasteurii* (see the Supporting Information, Figures S1 and S2). The progress curves of the inhibited reactions without enzyme–inhibitor preincubation were nonlinear, and the initial and steady-state velocities were inversely proportional to the concentration of benzisoselenazolones. The inhibitors were determined to be slow-binding and operated according to a two-step binding mechanism: a relatively rapid formation of enzyme-ligand complexes was followed by a limiting step of slow conformational changes, plausibly necessary for the formation of the S–Se bond. The binding mechanism of selected derivatives (esters **14e**–**h** and acids **1e**–**h**) was further studied through experiments that utilized dithiothreitol (DTT) as an agent that protected/reinstated the urease activity in the presence of inhibitors that bind to the thiol group of the cysteine in the active site. The inhibitors studied in this experiment were used at a saturating concentration of 5 μM, and the DTT concentration in all experiments was twice as high. First, DTT was added simultaneously to the inhibitor to assess its ability to disrupt the initial stage of EI complex formation (enzyme protection). No inhibitory activity was observed for compounds **14e**, **1e**, **14f**, and **1f** (Table 2). However, for both diethyl benzylphosphonates **14g** and **14h**, as well as phosphonic acids **1g** and **1h**, the velocity of ureolysis was only partially maintained (53.1–86.2%). It is noteworthy that benzylphosphonate esters **14g** and **14h**, in particular, were much more active inhibitors of bacterial ureases than their homologues **14e** and **14f**. In subsequent studies, DTT was added 30 min after the start of the reaction, allowing for the measurement of the stability of interactions already formed at the active site of urease (enzyme reactivation). In this case, enzymatic activity could be fully (100% for **14f**) or partially (27.3–76.3% for other derivatives) recovered. The inhibition with phosphonic acids **1e**–**h** appeared to be more susceptible to reconstitution than that achieved with the corresponding phosphonate esters (**14e**–**h**). Enzyme reactivation was also performed in an experiment where the enzymes were incubated with inhibitors for 2 h before the addition of substrate and measurement. Recovery in activity was significantly less pronounced and was observed only for compounds **14f**, **1f**, and **1h**, which were relatively weaker inhibitors (Table 2).

**Table 2:** DTT-Mediated *S. pasteurii* Urease Protection or Recovery from Inhibition

		retained enzyme activity [%], DTT added 0.5 h after the reaction initialization	
inhibitor ID	retained enzyme activity [%], DTT added before the reaction initialization	no enzyme–inhibitor preincubation	2 h enzyme–inhibitor preincubation
**14e**	100	27.3 ± 0.7	0
**1e**	100	31.9 ± 3.0	0
**14f**	100	100	36.5 ± 1.5
**1f**	100	76.3 ± 3.5	59.6 ± 3.9
**14g**	80.4 ± 2.7	53.8 ± 0.4	0
**1g**	86.2 ± 3.1	67.7 ± 7.2	0
**14h**	53.1 ± 0.8	51.8 ± 4.1	0
**1h**	56.7 ± 5.1	53.5 ± 1.9	13.5 ± 0.6

### Molecular Modeling

Molecular modeling demonstrated a regular mode of binding of diethyl phosphonate inhibitors to bacterial ureases. For the enzyme from *S. pasteurii*, this involves the assumed opening of the isoselenazolone ring and the formation of the covalent S–Se adduct with the Cys322 residue, which generally corresponds to what was previously reported for Ebselen^47^ and Ebselen-based inhibitors.^48,49^ Despite differences in the structure of the 2-substituent, representatives of alkyl (**14a**), aryl (**14f**), or extended alkylaryl compounds (**14l**) fit the active site similarly (Figure 4). The fit includes the incorporation of lipophilic portions of the inhibitors within the hydrophobic cleft formed by Met318, Met367, Ala366, and Ala170. The ethyl analogue **14a** is buried somewhat deeper than the others (Figure 4, panel A), while the rigid *p*-phenyl derivative **14f** is less favorably positioned despite having a large surface area for potential contacts (Figure 4, panel B). The **14l** xylyl compound is the most extended, yet it fills the cavity tightly due to increased flexibility and a gently twisted conformation (Figure 4, panel C). In all cases, a phosphonate group points toward Arg339, allowing the formation of a hydrogen bond between the phosphoryl oxygen atom and the NH of the guanidino group. Conversely, this interaction prevents the guanidino group from engaging in hydrogen bonding with the oxygen atom of the ligand’s amide group, which is typical for Ebselen and its nonphosphorylated derivatives.^47–49^ This difference may explain the lack of improvement in antiurease activity observed with the phosphonate functionalization compared with the unmodified structure. Finally, one of the ethyl residues of the phosphonate ester is situated near lipophilic residues Leu319 and Phe335.

**Figure 4:**
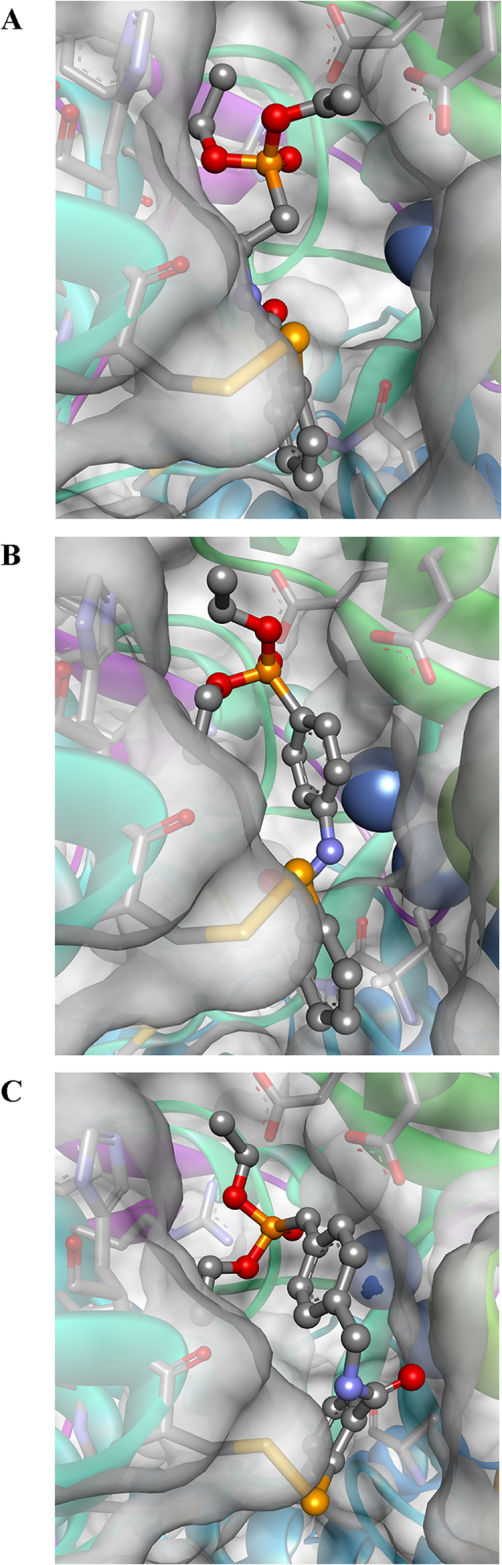
Modeled covalent complexes of inhibitors **14a** (panel A), **14f** (panel B), and **14l** (panel C) with *S. pasteurii* urease (PDB id 5G4H).^33^ The enzyme is depicted as a solid ribbon with a solvent-accessible surface, while nickel ions are represented as blue spheres.

The predicted mode of interaction for inhibitors **14a**, **14f**, and **14l** with *H. pylori* urease (see Figure S3) resembles that described for *S. pasteurii*. This is not surprising, as microbial ureases exhibit a high degree of regularity in the arrangement of the catalytic site region.^36,77^ These minor variations in the generally well-conserved mode of action of diethyl phosphonates with the enzymes rendered a more comprehensive discussion of the structure–activity relationship based on a molecular modeling complex. Besides conformational aspects of ligand–protein binding, other factors could influence inhibitor affinity, such as differences in the chemical reactivity of 2-alkyl versus 2-aryl and 2-benzylic benzisoselenazolones with the enzyme thiols.

The rational comments regarding the unexpectedly low potency of phosphonic acid **1a**–**h** were also problematic. The simultaneous presence of the two functional fragments in these structures appeared nonoptimal and did not yield the expected synergy of action (Figure 5 for *S. pasteurii* urease and Figure S4 for *H. pylori* urease). First, only alkylphosphonic acids **1a**–**d** could bind to the nickel ions present in the active sites, with the optimal distance found for the shortest homologue **1a** (Figures 5 and S4, panel A). The longest aliphatic homologue **1d** forms more constrained complexes; however, its structure offers an extended surface of lipophilic contacts (Figures 5 and S4, panel B). Other benzisoselenazolone analogues, phenyl- (e.g., **1f**; Figures 5 and S4, panel C) or benzylphosphonic acids, could not locate the acidic functionality in proximity to the metal ions due to the spatial size and rigidity of their structures. Instead, the phosphorus-containing substituents of these compounds are buried deeper in the hydrophobic environment of the active site clefts, with the phosphonic acid group in the proximity of the imidazole of His323/His322. Second, these compounds do not form any specific hydrogen bonds that typically involve NH of the guanidino group of Arg339 (*S. pasteurii* urease) bonded either with the amide oxygen atom of Ebselen^47^ or the phosphoryl oxygen atom of compounds **14a**–**l** (*vide supra*). Concluding, the structures of the complexes of phosphonic acids with ureases indicated the lack of concerted mixed-type interactions indispensable for favorable binding.

**Figure 5:**
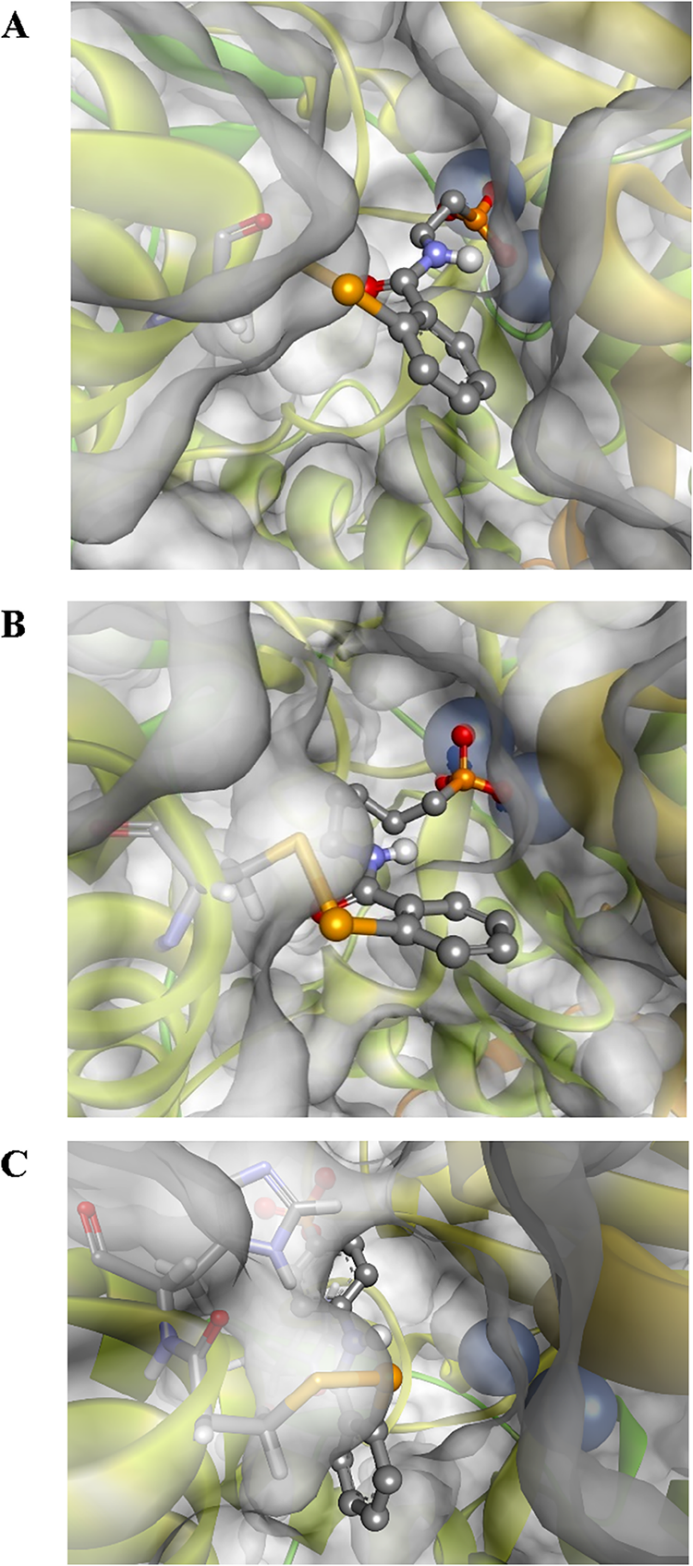
Modeled covalent complexes of inhibitors **1a** (panel A), **1d** (panel B), and **1f** (panel C) with *S. pasteurii* urease (PDB id 5G4H).^33^ The enzyme is depicted as a solid ribbon with a solvent-accessible surface, while nickel ions are represented as blue spheres.

### Antimicrobial Activity

The antimicrobial activity of the selected potent inhibitors of whole cell ureolysis (five phosphonate esters **14e**–**h** and **14k** and phosphonic acid **1h**) was further characterized on the *H. pylori* Tx30a strain. The antibacterial effect was moderate: the MIC values varied from 20 to 80 μM (8.24–33.0 μg/mL), were several fold higher than those previously reported for Ebselen,^49,56^ and approximately three orders of magnitude higher than that of amoxicillin, which was used as a positive control (Table 3). The most significant activity was observed for the best inhibitor **14f**. Interestingly, ester **14h** and corresponding acid **1h** were found to be equipotent.

**Table 3:** Antimicrobial Effects of Hybrid Organophosphorus/Selenium Urease Inhibitors, Compared to Amoxicillin, against *H. pylori* Tx30a

compound ID	MIC
**14e**	80 μM (33.0 μg/mL)
**14f**	20 μM (8.24 μg/mL)
**14g**	40 μM (17.0 μg/mL)
**14h**	80 μM (34.1 μg/mL)
**1h**	80 μM (29.6 μg/mL)
**14k**	40 μM (17.6 μg/mL)
amoxicillin	0.015 μg/mL

Furthermore, the potential of the phosphonate with the highest antibacterial activity (**14f**) to work in combination with the most common anti-*H. pylori* antibiotic (amoxicillin) was confirmed. Using checkerboard assays, an additive interaction between these compounds was shown by obtaining a fractional inhibitory concentration index (FICI) of 0.75 (Table 4). Interestingly, this value resulted from simultaneously halving the concentration of phosphonate **14f** and reducing amoxicillin by a factor of 4 or vice versa. This suggests that each compound operates through a different antibacterial mechanism against *H. pylori*. On the one hand, amoxicillin might facilitate the entry of phosphonate **14f** into *H. pylori* cells by weakening the cell envelope’s integrity.^78,79^ On the other hand, compound **14f**, as an Ebselen derivative, could inhibit the antioxidant function of some *H. pylori* proteins, thereby enhancing the antibacterial effects of free oxygen radicals produced as a side effect of amoxicillin activity.^57,80,81^

**Table 4:** Antimicrobial Effects of Diethyl Phosphonate **14f** in Combination with Amoxicillin against *H. pylori* Tx30a

	MIC [μg/mL]					
compound ID	alone	combination	fold change	FIC	FICI	interpretation
**14f**	8.24	4.12	×2↓	0.5	0.75	additive
amoxicillin	0.015	0.038	×4↓	0.25		
**14f**	8.24	2.06	×4↓	0.25		
amoxicillin	0.015	0.075	×2↓	0.5		

### Cytotoxicity

Finally, selected benzisoselenazolone inhibitors, six phosphonate esters (**14a**, **14d**, and **14e**–**h**) and one phosphonic acid (**1h**), were evaluated for their cytotoxic effects on eukaryotic cells, specifically normal fibroblasts from mouse embryo (BALB/3T3-L1) and epithelial cells from human embryo kidney (HEK-293). All compounds displayed moderate to low antiproliferative activity, with IC_50_ values typically ranging from 40 to 80 μM and in two cases exceeding 100 μM (Table 5). No substantial differences in growth inhibition were noted between benzisoselenazolones with different 2-substituent structures, specifically alkyl (**14a** and **14d**) versus phenyl (**14e** and **14f**) and benzyl (**14g** and **14h**), or between an ester (**14h**) and an acid (**1h**), for both BALB/3T3-L1 and HEK-293 cells. The organoselenium/phosphorus inhibitors were significantly less toxic than cisplatin, which was used as a positive control, by an order of magnitude for murine fibroblasts and more than two orders of magnitude for human kidney cells.

**Table 5:** Antiproliferative Activity of Selected Urease Inhibitors against Murine Embryonic Fibroblast Balb/3T3-L1 and Human Embryonic Kidney HEK-293 Cell Lines (IC_50_ Concentrations and Their 95% Confidence Intervals)

	IC_50_/IC_50_ 95CI [μM]	
compound ID	Balb/3T3-L1	HEK-293
**14a**	65.6/51.0–84.0	72.5/19.4–195
**14d**	>100	39.8/24.2–51.2
**14e**	76.9/52.3–127	>100
**14f**	43.7/35.3–54.4	62.5/26.7–177
**14g**	44.8/33.3–61.8	63.0/42.0–103
**14h**	64.6/54.3–76.9	42.4/31.4–59.1
**1h**	48.4/39.3–60.4	61.8/42.5–95.7
cisplatin	3.30/2.69–4.11	0.142/0.022–0.319

## Summary

The phosphonic acid group is a prominent pharmacophoric functionality in numerous antiviral, antimicrobial, and anticancer drugs.^82^ Regarding the mode of action, this group enhances the specific molecular recognition of bioactive compounds by target receptors, which frequently involves complexation with metal ions. In applying this general rationale to the present work, we combined the benzisoselenazolone structure—characterized by its strong antiureolytic activity—with the phosphonate functionality, which is designed to bind to the nickel ions present in the enzyme’s active site. We expected an enhancement in the potency of the hybrid compounds; however, the effect of substitution was not cooperative. The inhibitory activity of phosphonic acids **1a**–**h** against the model urease of *S. pasteurii* varied within a relatively narrow range (*K*_i_ = 108–436 nM), indicating that the structural diversity of the phosphonoalkyl/aryl portion barely influenced enzyme–inhibitor interactions. The differentiation of the SAR data was much more pronounced for diethyl phosphonates **14a**–**l** (*K*_i_ = 5.06–453 nM) and involved almost two orders of magnitude between the most potent extended arylalkyl analogues and the phenyl derivatives of the lowest affinity (despite the absence of significant variations in the binding mode illustrated by molecular modeling). Apparently, none of the representatives from the two series of compounds outscored the activity of Ebselen, the prototypical benzisoselenazolone inhibitor of bacterial urease (*K*_i_* = 2.11 nM for *S. pasteurii*; Figure 6).^47^

**Figure 6:**
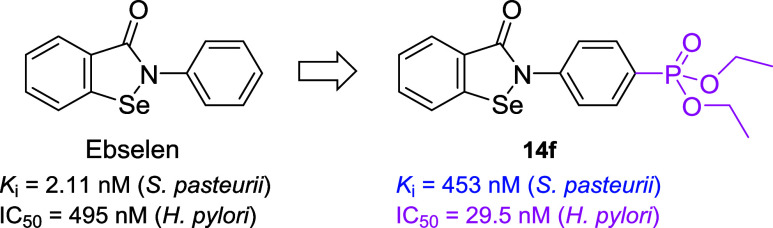
Comparison of inhibitory activity of Ebselen and its *para*-diethyl phosphonate analogue **14f** against urease of *S. pasteurii* and ureolysis of live cells of *H. pylori*.

Although phosphorylation seemed to deteriorate the inhibition of a purified bacterial enzyme from *S. pasteurii*, it significantly improved the prevention of ureolysis exhibited by pathogenic *H. pylori* cells. Phosphonic acids **1a**–**h** were again less potent (IC_50_ = 187–2106 nM), which was expected due to the negative charge(s) that make the functional group very polar and poorly bioavailable because of inadequate permeability through microbial cell walls. To mitigate these unfavorable physicochemical properties, the polarity of phosphonic acids is often masked in prodrug forms using various protecting groups.^83^ Ethyl esters are generally not applied for constructing prodrugs (not sufficiently cleavable), but in our case, they served a protective role. The structure of diethyl phosphonate enabled us to achieve unprecedented antiureolytic activity of compounds **14a**–**l** (IC_50_ = 29.5–299 nM), which has never been reported for intact *H. pylori* cells. All developed compounds exhibited higher potency than Ebselen (IC_50_ = 495 nM; Figure 6), with IC_50_ values 12–17-fold lower for compounds **14e**, **14f**, and **14j**–**l**. This effect was clearly the result of improved physicochemical properties combined with high complementarity to the target enzyme: the appropriate size and lipophilicity of the inhibitors balanced with the ability to form a specific hydrogen bond (differing from the binding postulated for Ebselen). The excellent antiureolytic activity in cells, advantageous bioavailability, and reasonable cytotoxicity make the developed compounds promising adjuvants in combination therapies against *H. pylori* infections, aimed at eliminating the major bacterial virulence factor. A cooperative antibacterial action of diethyl phosphonate **14f** with amoxicillin was preliminarily demonstrated by decreasing the MIC values when both compounds were used simultaneously against *H. pylori*; however, understanding the molecular mechanisms governing these effects requires further investigation.

## Experimental Section

### Chemistry

#### General Methods

All reagents used were purchased from the following commercial suppliers: Merck Poland–Sigma-Aldrich, Avantor Performance Materials Poland, Chemland Poland, and Stanlab Poland. They were of analytical grade and used without further purification unless otherwise stated. Anhydrous dichloromethane was prepared prior to use by distillation over phosphorus pentoxide. Triethylamine was distilled and stored in potassium hydroxide. Microwave-induced reactions were performed on an Initiator^+^ SP Wave Biotage apparatus. Reactions were monitored by thin-layer chromatography (TLC) on 0.25 mm silica gel plates with fluorescent labels (silica gel 60F254, Merck), and components were visualized using UV light absorption and/or incubation with iodine. Flash chromatography was performed on a Teledyne ISCO CombiFlash using RediSep Gold silica or C18 columns. High-performance liquid chromatography was conducted on a Shimadzu system with the ReproSil-Pur Basic-C18 column from Maisch, SHIM-POL. The melting points were determined on an Electrothermal IA 91100 digital melting point apparatus using the standard open capillary method. The ^1^H, ^13^C, ^31^P and ^77^Se NMR spectra were recorded in CD_3_OD or D_2_O on a Jeol ECZ 400S spectrometer at frequencies of 399.8 MHz (^1^H), 151.0 or 100.5 MHz (^13^C), 161.8 MHz (^31^P), and 76.2 MHz (^77^Se), respectively, at 295 K. Chemical shifts were reported in parts per million (ppm, δ) downfield from tetramethylsilane. Residual solvent central signals were recorded as follows: CD_3_OD, δ_H_ = 3.31 ppm, δ_C_ = 49.00 ppm, and D_2_O, δ_H_ = 4.79 ppm. Coupling patterns were described as singlet (*s*), doublet (*d*), triplet (*t*), quartet (*q*), quintet (*quin*), and multiplet (*m*). High-resolution mass spectra (HRMS) were recorded by using an electron spray ionization (ESI) technique on a Waters LCT Premier XE spectrometer. Analytical reversed-phase high-performance liquid chromatography was performed using the UFLC Shimadzu system and the CHROMSHELL C18-XB HPLC column, 4.6 × 75 mm (0 min, 10% B → 1 min, 10% B → 12.5 min, 90% B → 15 min, 90% B → 17 min, 10% B, flow rate 0.9 mL/min for compounds **14a**–**l**, **1d**–**h**, **15a**, and **15b**, or 0 min, 0% B → 10 min, 40% B → 13 min, 90% B → 14 min, 90% B → 15 min, 0% B, flow rate 0.9 mL/min for compounds **1a**–**c**). Solvent A is 0.1% TFA in water; solvent B is 0.1% TFA in acetonitrile. Chromatograms were recorded at wavelengths of 222 and 254 nm using background compensation. The final 2-benzyl-1,2-benzisoselenazol-3­(*2H*)-ones **14a**–**l**, **1d**–**h**, **15a**, and **15b** gave satisfactory NMR and HRMS spectra and were *>*95% pure.

#### Diethyl Phosphonates, Derivatives of 2-Substituted 1,2-Benzisoselenazol-3­(*2H*)-ones (**14a**–**l**)

Compounds **14a**–**l** were synthesized through the aminolysis of 2-(chloroseleno)­benzoyl chloride (**13**), obtained from anthranilic acid (**12**), with diethyl aminophosphonates **5a**–**l** (detailed procedures for their preparation are described in the Supporting Information), as outlined in the procedure previously reported with minor modifications.^73,74^ Anhydrous triethylamine (2.5 equiv) and 2-(chloroseleno)­benzoyl chloride (**13**, 1 eq., dissolved in anhydrous dichloromethane, typically 2–5 mmol in 1 mL) were then added through a septum to a solution of a diethyl aminophosphonate **5a**–**l** (1.0 equiv) in anhydrous dichloromethane (5 mL). After the mixture was stirred at room temperature for 48 h, a 5% aqueous sodium bicarbonate solution (20 mL) was added, and the product was extracted with methylene chloride (50 mL). The organic phase was washed with a 5% aqueous sodium bisulfate solution (20 mL) and brine (20 mL) and then dried over anhydrous Na_2_SO_4_. The drying agent was filtered off, and the filtrate was concentrated *in vacuo*. The residue was purified by flash chromatography (using a gradient of dichloromethane/methanol, 100/0 → 90/10 vv).

##### Diethyl 2-(3-oxobenzo­[*d*]­[1,2]­selenazol-2­(3*H*)-yl)­ethylphosphonate (**14a**)

Light orange solid, yield 63%, mp 123–124 °C. ^1^H NMR (400 MHz, CD_3_OD) δ 7.95–7.92 (m, 2H), 7.62 (ddd, *J*_HH_ = 8.3, 7.2, 1.4 Hz, 1H), 7.45 (ddd, *J*_HH_ = 8.0, 7.2, 1.0 Hz, 1H), 4.15–4.01 (m, 6H), 2.35–2.26 (m, 2H), 1.29 (t, *J*_HH_ = 7.1 Hz, 6H). ^13^C NMR (101 MHz, CD_3_OD) δ 169.35, 141.21, 133.18, 128.75, 128.73, 127.20, 126.40, 63.54 (d, *J*_CP_ *=* 6.6 Hz), 39.84 (d, *J*_CP_ *=* 2.1 Hz), 26.66 (d, *J*_CP_ *=* 138.9 Hz), 16.62 (d, *J*_CP_ *=* 6.2 Hz). ^31^P NMR (162 MHz, CD_3_OD) δ 28.88. ^77^Se NMR (76 MHz, CD_3_OD) δ 914.51. HRMS (ESI) *m*/*z* calculated for C_13_H_18_NO_4_PSe+H^+^ 364.0217, found 364.0226.

##### Diethyl 3-(3-oxobenzo­[*d*]­[1,2]­selenazol-2­(3*H*)-yl)­propylphosphonate (**14b**)

Brown oil, yield 68%. ^1^H NMR (400 MHz, CD_3_OD) δ 7.95–7.92 (m, 2H), 7.61 (ddd, *J*_HH_ *=* 8.3, 7.2, 1.4 Hz, 1H), 7.44 (ddd, *J*_HH_ *=* 8.0, 7.2, 1.0 Hz, 1H), 4.13–4.03 (m, 4H), 3.90 (t, *J*_HH_ *=* 6.8 Hz, 2H), 2.03–1.94 (m, 2H), 1.91–1.82 (m, 2H), 1.30 (t, *J*_HH_ *=* 7.1 Hz, 6H). ^13^C NMR (101 MHz, CD_3_OD) δ 169.39, 140.93, 133.10, 128.85, 128.82, 127.21, 126.36, 63.30 (d, *J*_CP_ *=* 6.6 Hz), 45.40 (d, *J*_CP_ *=* 19.0 Hz), 24.45 (d, *J*_CP_ *=* 4.7 Hz), 23.07 (d, *J*_CP_ *=* 142.2 Hz), 16.71 (d, *J*_CP_ *=* 6.0 Hz). ^31^P NMR (162 MHz, CD_3_OD) δ 32.83. ^77^Se NMR (76 MHz, CD_3_OD) δ 907.96. HRMS (ESI) *m*/*z* calculated for C_14_H_20_NO_4_PSe+H^+^ 378.0373, found 378.0396.

##### Diethyl 4-(3-oxobenzo­[*d*]­[1,2]­selenazol-2­(3*H*)-yl)­butylphosphonate (**14c**)

Brown oil, yield 73%. ^1^H NMR (400 MHz, CD_3_OD) δ 7.95–7.92 (m, 2H), 7.62 (ddd, *J*_HH_ *=* 8.2, 7.2, 1.4 Hz, 1H), 7.45 (ddd, *J*_HH_ = 8.7, 7.2, 1.0 Hz, 1H), 4.13–4.01 (m, 4H), 3.88 (t, *J*_HH_ *=* 6.8 Hz, 2H), 1.92–1.82 (m, 4H), 1.68–1.61 (m, 2H), 1.27 (t, *J*_HH_ *=* 7.1 Hz, 6H). ^13^C NMR (101 MHz, CD_3_OD) δ 169.31, 140.89, 133.03, 129.00, 128.82, 127.19, 126.33, 63.15 (d, *J*_CP_ *=* 6.6 Hz), 44.46, 31.86 (d, *J*_CP_ *=* 16.2 Hz), 25.19 (d, *J*_CP_ *=* 140.5 Hz), 20.42 (d, *J*_CP_ *=* 5.1 Hz), 16.69 (d, *J*_CP_ *=* 6.1 Hz). ^31^P NMR (162 MHz, CD_3_OD) δ 33.56. ^77^Se NMR (76 MHz, CD_3_OD) δ 902.97. HRMS (ESI) *m*/*z* calculated for C_15_H_22_NO_4_PSe+H^+^ 392.0530, found 392.0533.

##### Diethyl 5-(3-oxobenzo­[*d*]­[1,2]­selenazol-2­(3*H*)-yl)­pentylphosphonate (**14d**)

Brown oil, yield 81%. ^1^H NMR (400 MHz, CD_3_OD) δ 7.94–7.91 (m, 2H), 7.61 (ddd, *J*_HH_ *=* 8.2, 7.2, 1.4 Hz, 1H), 7.44 (ddd, *J*_HH_ *=* 7.9, 7.2, 1.1 Hz, 1H), 4.09–4.01 (m, 4H), 3.84 (t, *J*_HH_ *=* 7.0 Hz, 2H), 1.83–1.72 (m, 4H), 1.67–1.59 (m, 2H), 1.52–1.44 (m, 2H), 1.29 (t, *J*_HH_ *=* 7.0 Hz, 6H). ^13^C NMR (101 MHz, CD_3_OD) δ 169.23, 140.87, 132.99, 129.08, 128.80, 127.17, 126.30, 63.09 (d, *J*_CP_ *=* 6.6 Hz), 45.14, 30.91, 28.39 (d, *J*_CP_ *=* 16.1 Hz), 25.62 (d, *J*_CP_ *=* 140.4 Hz), 23.09 (d, *J*_CP_ *=* 5.2 Hz), 16.71 (d, *J*_CP_ *=* 6.0 Hz). ^31^P NMR (162 MHz, CD_3_OD) δ 33.90. ^77^Se NMR (76 MHz, CD_3_OD) δ 902.95. HRMS (ESI) *m*/*z* calculated for C_16_H_24_NO_4_PSe+H^+^ 406.0687, found 406.0688.

##### Diethyl 3-(3-oxobenzo­[*d*]­[1,2]­selenazol-2­(3*H*)-yl)­phenylphosphonate (**14e**)

Light yellow crystals, yield 53%, mp 107–110 °C. ^1^H NMR (400 MHz, CD_3_OD) δ 8.09–7.98 (m, 3H), 7.88 (ddd, *J*_HH_ *=* 7.8, 1.2 Hz, *J*_HP_ *=* 2.3 Hz, 1H), 7.73–7.64 (m, 3H), 7.50 (ddd, *J =* 8.1, 7.3, 1.1 Hz, 1H), 4.20–4.12 (m, 4H), 1.35 (t, *J*_HH_ *=* 7.0 Hz, 6H). ^13^C NMR (101 MHz, CD_3_OD) δ 166.82, 139.93 (d, *J*_CP_ *=* 18.9 Hz), 139.37, 132.58, 129.80 (d, *J*_CP_ *=* 16.5 Hz), 129.41 (d, *J*_CP_ *=* 3.4 Hz), 129.22 (d, *J*_CP_ *=* 9.4 Hz), 129.02 (d, *J*_CP_ *=* 189.9 Hz), 128.19, 127.95, 127.83, 126.35, 125.03, 62.79 (d, *J*_CP_ *=* 5.9 Hz), 15.31 (d, *J*_CP_ *=* 6.3 Hz). ^31^P NMR (162 MHz, CD_3_OD) δ 18.30. ^77^Se NMR (76 MHz, CD_3_OD) δ 969.96. HRMS (ESI) *m*/*z* calculated for C_17_H_18_NO_4_PSe+H^+^ 412.0217, found 412.0180.

##### Diethyl 4-(3-oxobenzo­[*d*]­[1,2]­selenazol-2­(3*H*)-yl)­phenylphosphonate (**14f**)

Light yellow crystals, yield 74%, mp 165–170 °C. ^1^H NMR (400 MHz, CD_3_OD) δ 8.02–7.97 (m, 2H), 7.88–7.85 (m, 4H), 7.69 (ddd, *J*_HH_ *=* 8.3, 7.2, 1.4 Hz, 1H), 7.50 (ddd, *J*_HH_ *=* 8.1, 7.2, 1.0 Hz, 1H), 4.19–4.08 (m, 4H), 1.34 (t, *J*_HH_ *=* 7.1 Hz, 6H). ^13^C NMR (101 MHz, CD_3_OD) δ 168.11, 145.23, 140.57, 134.02, 133.91, 129.56, 129.40, 127.68, 126.29, 126.14 (d, *J*_CP_ *=* 193.3 Hz), 125.84 (d, *J*_CP_ *=* 15.5 Hz), 63.92 (d, *J*_CP_ *=* 5.7 Hz), 16.62 (d, *J*_CP_ *=* 6.3 Hz). ^31^P NMR (162 MHz, CD_3_OD) δ 19.05. ^77^Se NMR (76 MHz, CD_3_OD) δ 968.38. HRMS (ESI) *m*/*z* calculated for C_17_H_18_NO_4_PSe+H^+^ 412.0217, found 412.0218.

##### Diethyl 3-(3-oxobenzo­[*d*]­[1,2]­selenazol-2­(3*H*)-yl)­benzylphosphonate (**14g**)

Yellow oil, yield 70%. ^1^H NMR (400 MHz, CD_3_OD) δ 8.01–7.97 (m, 2H), 7.68 (ddd, *J*_HH_ *=* 8.3, 7.2, 1.4 Hz, 1H), 7.57–7.48 (m, 3H), 7.44–7.40 (m, 1H), 7.28 (ddd, *J*_HH_ *=* 7.8, 1.3 Hz, *J*_HP_ *=* 2.8 Hz, 1H), 4.13–4.04 (m, 4H), 3.32 (d, *J*_HP_ *=* 21.8 Hz, 2H), 1.29 (t, *J*_HH_ *=* 7.1 Hz, 6H). ^13^C NMR (101 MHz, CD_3_OD) δ 167.97, 140.95, 140.64 (d, *J*_CP_ *=* 3.3 Hz), 134.42 (d, *J*_CP_ *=* 9.3 Hz), 133.65, 130.45 (d, *J*_CP_ *=* 3.1 Hz), 129.58 (d, *J*_CP_ *=* 6.6 Hz), 129.39, 129.29, 128.12 (d, *J*_CP_ *=* 6.5 Hz), 127.53, 126.27, 125.34 (d, *J*_CP_ *=* 3.6 Hz), 63.85 (d, *J*_CP_ *=* 6.9 Hz), 33.48 (d, *J*_CP_ *=* 137.8 Hz), 16.72 (d, *J*_CP_ *=* 6.0 Hz). ^31^P NMR (162 MHz, CD_3_OD) δ 27.61. ^77^Se NMR (76 MHz, CD_3_OD) δ 969.06. HRMS (ESI) *m*/*z* calculated for C_18_H_20_NO_4_PSe+H^+^ 426.0374, found 426.0385.

##### Diethyl 4-(3-oxobenzo­[*d*]­[1,2]­selenazol-2­(3*H*)-yl)­benzylphosphonate (**14h**)

Light yellow crystals, yield 66%, mp 140–142 °C. ^1^H NMR (400 MHz, CD_3_OD) δ 8.01–7.97 (m, 2H), 7.68 (ddd, *J*_HH_ *=* 8.3, 7.2, 1.4 Hz, 1H), 7.57 (dd, *J*_HH_ *=* 8.6 Hz, *J*_HP_ = 1.1 Hz, 2H), 7.50 (ddd, *J*_HH_ *=* 7.5, 7.3, 1.0 Hz, 1H), 7.42 (dd, *J*_HH_ *=* 8.5 Hz, *J*_HP_ = 2.6 Hz, 2H), 4.12–4.04 (m, 4H), 3.30 (d, *J*_HP_ *=* 21.7 Hz, 2H), 1.29 (t, *J*_HH_ *=* 7.1 Hz, 6H). ^13^C NMR (101 MHz, CD_3_OD) δ 167.96, 140.91, 139.35, 133.63, 131.89 (d, *J*_CP_ *=* 6.6 Hz), 131.70 (d, *J*_CP_ *=* 9.5 Hz), 129.38, 129.30, 127.52, 126.67 (d, *J*_CP_ *=* 3.2 Hz), 126.25, 63.78 (d, *J*_CP_ *=* 6.9 Hz), 33.22 (d, *J*_CP_ *=* 137.9 Hz), 16.68 (d, *J*_CP_ *=* 6.0 Hz). ^31^P NMR (162 MHz, CD_3_OD) δ 27.71. ^77^Se NMR (76 MHz, CD_3_OD) δ 968.23. HRMS (ESI) *m*/*z* calculated for C_18_H_20_NO_4_PSe+H^+^ 426.0374, found 426.0365.

##### Diethyl 2-(3-(3-oxobenzo­[*d*]­[1,2]­selenazol-2­(3*H*)-yl)­phenyl)­ethylphosphonate (**14i**)

Yellow oil, yield 56%. ^1^H NMR (400 MHz, CD_3_OD) δ 8.01–7.96 (m, 2H), 7.68 (ddd, *J*_HH_ *=* 8.2, 7.3, 1.4 Hz, 1H), 7.52–7.48 (m, 2H), 7.42–7.38 (m, 2H), 7.25–7.22 (m, 1H), 4.12–4.05 (m, 4H), 2.99–2.91 (m, 2H), 2.22–2.13 (m, 2H), 1.30 (t, *J*_HH_ *=* 7.1 Hz, 6H). ^13^C NMR (101 MHz, CD_3_OD) δ 167.98, 143.64 (d, *J*_CP_ *=* 15.8 Hz), 141.01, 140.56, 133.61, 130.59, 129.39, 129.28, 128.02, 127.51, 126.67, 126.24, 124.84, 63.28 (d, *J*_CP_ *=* 6.7 Hz), 29.34 (d, *J*_CP_ *=* 4.6 Hz), 27.55 (d, *J*_CP_ *=* 139.5 Hz), 16.71 (d, *J*_CP_ *=* 6.1 Hz). ^31^P NMR (162 MHz, CD_3_OD) δ 32.28. ^77^Se NMR (76 MHz, CD_3_OD) δ 969.66. HRMS (ESI) *m*/*z* calculated for C_19_H_22_NO_4_PSe+H^+^ 440.0530, found 440.0538.

##### Diethyl 2-(4-(3-oxobenzo­[*d*]­[1,2]­selenazol-2­(3*H*)-yl)­phenyl)­ethylphosphonate (**14j**)

Light yellow crystals, yield 61%, mp 137–139 °C. ^1^H NMR (400 MHz, CD_3_OD) δ 8.00–7.96 (m, 2H), 7.67 (ddd, *J*_HH_ *=* 8.2, 7.2, 1.4 Hz, 1H), 7.52–7.47 (m, 3H), 7.37–7.34 (m, 2H), 4.12–4.05 (m, 4H), 2.96–1.89 (m, 2H), 2.17–2.11 (m, 2H), 1.29 (t, *J*_HH_ *=* 7.1 Hz, 6H). ^13^C NMR (101 MHz, CD_3_OD) δ 167.99, 141.15 (d, *J*_CP_ *=* 15.8 Hz), 140.99, 138.70, 133.58, 130.20, 129.36, 129.27, 127.51, 126.95, 126.25, 63.27 (d, *J*_CP_ *=* 6.6 Hz), 29.06 (d, *J*_CP_ *=* 4.6 Hz), 27.65 (d, *J*_CP_ *=* 139.2 Hz), 16.72 (d, *J*_CP_ *=* 6.1 Hz). ^31^P NMR (162 MHz, CD_3_OD) δ 32.30. ^77^Se NMR (76 MHz, CD_3_OD) δ 968.14. HRMS (ESI) *m*/*z* calculated for C_19_H_22_NO_4_PSe+H^+^ 440.0530, found 440.0523.

##### Diethyl 3-((3-oxobenzo­[*d*]­[1,2]­selenazol-2­(3*H*)-yl)­methyl)­benzylphosphonate (**14k**)

Yellow oil, yield 52%. ^1^H NMR (400 MHz, CD_3_OD) δ 7.97­(ddd, *J*_HH_ *=* 7.9, 1.4, 0.6 Hz, 1H), 7.89 (ddd, *J*_HH_ *=* 8.1, 0.9, 0.9 Hz, 1H), 7.61 (ddd, *J*_HH_ *=* 8.3, 7.2, 1.4 Hz, 1H), 7.46 (ddd, *J*_HH_ *=* 8.1, 7.2, 1.0 Hz, 1H), 7.39–7.23 (m, 4H), 4.99 (s, 2H), 4.03–3.93 (m, 4H), 3.23 (d, *J*_HP_ *=* 21.8 Hz, 2H), 1.17 (t, *J*_HH_ *=* 7.1 Hz, 6H). ^13^C NMR (101 MHz, CD_3_OD) 169.21, 141.14, 139.37 (d, *J*_CP_ *=* 3.3 Hz), 133.54 (d, *J*_CP_ *=* 9.4 Hz), 133.14, 130.78 (d, *J*_CP_ *=* 6.5 Hz), 129.99 (d, *J*_CP_ *=* 3.2 Hz), 128.98, 128.89, 128.01, 127.97, 127.21, 126.33, 63.75 (d, *J*_CP_ *=* 6.9 Hz), 54.81, 33.61 (d, *J*_CP_ *=* 137.7 Hz), 16.61 (d, *J*_CP_ *=* 6.0 Hz). ^31^P NMR (162 MHz, CD_3_OD) δ 27.84. ^77^Se NMR (76 MHz, CD_3_OD) 903.92. HRMS (ESI) *m*/*z* calculated for C_19_H_22_NO_4_PSe+H^+^ 440.0530, found 440.0538.

##### Diethyl 4-((3-oxobenzo­[*d*]­[1,2]­selenazol-2­(3*H*)-yl)­methyl)­benzylphosphonate (**14l**)

Light yellow crystals, yield 56%, mp 152–153 °C. ^1^H NMR (400 MHz, CD_3_OD) δ 7.96 (ddd, *J*_HH_ *=* 7.8, 1.4, 0.6 Hz, 1H), 7.89 (ddd, *J*_HH_ *=* 8.1, 0.9, 0.9 Hz, 1H), 7.61 (ddd, *J*_HH_ *=* 8.2, 7.2, 1.4 Hz, 1H), 7.45 (ddd, *J*_HH_ *=* 8.1, 7.2, 1.0 Hz, 1H), 7.33–7.31 (m, 4H), 4.98 (d, *J*_HP_ *=* 1.7 Hz, 2H), 4.06–3.97 (m, 4H), 3.24 (d, *J*_HP_ *=* 21.7 Hz, 2H), 1.24 (t, *J*_HH_ *=* 7.1 Hz, 6H). ^13^C NMR (101 MHz, CD_3_OD) δ 169.23, 141.11, 137.76 (d, *J*_CP_ *=* 3.9 Hz), 133.12, 132.72 (d, *J*_CP_ *=* 9.3 Hz), 131.43 (d, *J*_CP_ *=* 6.6 Hz), 129.53 (d, *J*_CP_ *=* 3.2 Hz), 128.94, 128.91, 127.21, 126.31, 63.74 (d, *J*_CP_ *=* 6.9 Hz), 54.81, 33.40 (d, *J*_CP_ *=* 137.9 Hz), 16.65 (d, *J*_CP_ *=* 6.0 Hz). ^31^P NMR (162 MHz, CD_3_OD) δ 27.97. ^77^Se NMR (76 MHz, CD_3_OD) 903.24. HRMS (ESI) *m*/*z* calculated for C_19_H_22_NO_4_PSe+H^+^ 440.0530, found 440.0541.

#### Phosphonic Acids, Derivatives of 2-Substituted 1,2-Benzisoselenazol-3­(*2H*)-ones (**1a**–**h**)

Compounds **1a**–**h** were obtained through the microwave-heated acidic hydrolysis of **14a**–**h**. An ester **14a**–**h** (1.0 mmol) dissolved in acetic acid (1 mL) and 36–38% hydrochloric acid (1 mL) was heated in a microwave oven at 140 °C for 20 min. After the mixture was cooled to room temperature and volatiles evaporated *in vacuo*, the residue was purified by reverse-phase HPLC chromatography (using a gradient of acetonitrile/water, 10/90 → 90/10 vv + 0.05% TFA).

##### 2-(3-Oxobenzo­[*d*]­[1,2]­selenazol-2­(3*H*)-yl)­ethylphosphonic acid (**1a**)

White solid, yield 59%, mp 215–217 °C. ^1^H NMR (400 MHz, D_2_O) δ 7.80–7.77 (m, 2H), 7.60 (ddd, *J*_HH_ *=* 8.4, 7.2, 1.3 Hz, 1H), 7.44 (ddd, *J*_HH_ *=* 8.0, 7.2, 1.1 Hz, 1H), 3.98–3.93 (m, 2H), 1.99–1.89 (m, 2H). ^13^C NMR (101 MHz, D_2_O) δ 167.98, 137.43, 133.05, 127.88, 127.22, 126.12, 125.09, 44.29 (d, *J*_CP_ *=* 7.2 Hz), 27.02 (d, *J*_CP_ *=* 130.7 Hz). ^31^P NMR (162 MHz, D_2_O) δ 17.16. ^77^Se NMR (76 MHz, D_2_O) δ 931.78. HRMS (ESI) *m*/*z* calculated for C_9_H_10_NO_4_PSe+H^+^ 307.9591, found 307.9597.

##### 3-(3-Oxobenzo­[*d*]­[1,2]­selenazol-2­(3*H*)-yl)­propylphosphonic acid (**1b**)

White solid, yield 63%, mp 222–223 °C. ^1^H NMR (400 MHz, D_2_O) δ 7.75–7.73 (m, 2H), 7.57 (ddd, *J*_HH_ *=* 8.5, 7.2, 1.3 Hz, 1H), 7.39 (ddd, *J*_HH_ *=* 8.0, 7.2, 1.0 Hz, 1H), 3.78 (t, *J*_HH_ *=* 7.1 Hz, 2H), 1.94–1.84 (m, 2H), 1.50–1.42 (m, 2H). ^13^C NMR (101 MHz, D_2_O) δ 168.37, 139.43, 132.19, 127.31, 126.76, 126.22, 124.50, 46.08 (d, *J*_CP_ *=* 19.8 Hz), 25.96 (d, *J*_CP_ *=* 131.7 Hz), 25.13 (d, *J*_CP_ *=* 3.6 Hz). ^31^P NMR (162 MHz, D_2_O) δ 22.77. ^77^Se NMR (76 MHz, D_2_O) δ 925.64. HRMS (ESI) *m*/*z* calculated for C_10_H_12_NO_4_PSe+H^+^ 321.9748, found 321.9755.

##### 4-(3-Oxobenzo­[*d*]­[1,2]­selenazol-2­(3*H*)-yl)­butylphosphonic acid (**1c**)

White solid, yield 66%, mp 226–228 °C. ^1^H NMR (400 MHz, D_2_O) δ 7.78–7.73 (m, 2H), 7.55 (ddd, *J*_HH_ *=* 8.2, 7.2, 1.4 Hz, 1H), 7.41–7.37 (m, 1H), 3.70 (t, *J*_HH_ *=* 7.4 Hz, 2H), 1.72 (quin, *J*_HH_ *=* 7.4 Hz, 2H), 1.56–1.50 (m, 2H), 1.44–1.35 (m, 2H). ^13^C NMR (101 MHz, D_2_O) δ 168.51, 138.19, 132.08, 127.89, 127.30, 126.32, 125.49, 44.23, 31.49 (d, *J*_CP_ *=* 17.2 Hz), 29.02 (d, *J*_CP_ *=* 130.5 Hz), 21.56 (d, *J*_CP_ *=* 3.8 Hz). ^31^P NMR (162 MHz, D_2_O) δ 23.38. ^77^Se NMR (76 MHz, D_2_O) δ 923.75. HRMS (ESI) *m*/*z* calculated for C_11_H_14_NO_4_PSe+H^+^ 335.9904, found 335.9909.

##### 5-(3-Oxobenzo­[*d*]­[1,2]­selenazol-2­(3*H*)-yl)­pentylphosphonic acid (**1d**)

White solid, yield 70%, mp 190–192 °C. ^1^H NMR (400 MHz, D_2_O) δ 7.72–7.69 (m, 2H), 7.54 (ddd, *J*_HH_ *=* 8.3, 7.2, 1.3 Hz, 1H), 7.36 (ddd, *J*_HH_ *=* 8.0, 7.2, 1.0 Hz, 1H), 3.70 (t, *J*_HH_ *=* 7.1 Hz, 2H), 1.67 (quin, *J*_HH_ *=* 7.3 Hz, 2H), 1.55–1.29 (m, 6H). ^13^C NMR (101 MHz, D_2_O) δ 168.22, 139.34, 132.11, 127.26, 126.79, 126.18, 124.45, 44.92, 29.42, 28.88 (d, *J*_CP_ *=* 131.4 Hz), 27.73 (d, *J*_CP_ *=* 17.8 Hz), 23.50 (d, *J*_CP_ *=* 4.1 Hz). ^31^P NMR (162 MHz, D_2_O) δ 24.35. ^77^Se NMR (76 MHz, D_2_O) δ 923.22. HRMS (ESI) *m*/*z* calculated for C_12_H_16_NO_4_PSe+H^+^ 350.0061, found 350.0070.

##### 3-(3-Oxobenzo­[*d*]­[1,2]­selenazol-2­(3*H*)-yl)­phenylphosphonic acid (**1e**)

White solid, yield 60%, mp 223–225 °C. ^1^H NMR (400 MHz, D_2_O) δ 7.89–7.84 (m, 2H), 7.78–7.66 (m, 3H), 7.54–7.46 (m, 3H). ^13^C NMR (101 MHz, D_2_O) δ 167.95, 160.49, 142.71 (d, *J*_CP_ *=* 165.6 Hz), 140.08, 136.96 (d, *J*_CP_ *=* 15.7 Hz), 132.88, 130.11 (d, *J*_CP_ *=* 8.2 Hz), 129.02 (d, *J*_CP_ *=* 13.5 Hz), 128.10 (d, *J*_CP_ *=* 9.5 Hz), 128.01, 126.82, 126.66 (t, *J*_CP_ *=* 13.4 Hz), 124.63. ^31^P NMR (162 MHz, D_2_O) δ 10.37. ^77^Se NMR (76 MHz, D_2_O) δ 997.23. HRMS (ESI) *m*/*z* calculated for C_13_H_10_NO_4_PSe+H^+^ 355.9591, found 355.9593.

##### 4-(3-Oxobenzo­[*d*]­[1,2]­selenazol-2­(3*H*)-yl)­phenylphosphonic acid (**1f**)

White solid, yield 65%, mp >260 °C. ^1^H NMR (400 MHz, D_2_O) δ 7.83–7.75 (m, 4H), 7.62 (ddd, *J*_HH_ *=* 8.5, 7.2, 1.3 Hz, 1H), 7.47–7.41 (m, 3H). ^13^C NMR (101 MHz, D_2_O) δ 167.72, 162.00, 140.83 (d, *J*_CP_ = 166.8 Hz), 139.89, 137.78 (d, *J*_CP_ *=* 3.4 Hz), 132.81, 131.44 (d, *J*_CP_ *=* 9.5 Hz), 127.90, 126.57, 126.37, 125.61 (d, *J*_CP_ *=* 13,1 Hz), 124.49. ^31^P NMR (162 MHz, D_2_O) δ 10.84. ^77^Se NMR (76 MHz, D_2_O) δ 997.91. HRMS (ESI) *m*/*z* calculated for C_13_H_10_NO_4_PSe+H^+^ 355.9591, found 355.9673.

##### 3-(3-Oxobenzo­[*d*]­[1,2]­selenazol-2­(3*H*)-yl)­benzylphosphonic acid (**1g**)

White solid, yield 48%, mp 200–205 °C. ^1^H NMR (400 MHz, D_2_O) δ 7.94–7.87 (m, 2H), 7.73–7.68 (m, 1H), 7.55–7.51 (m, 1H), 7.44–7.36 (m, 3H), 7.30 (ddd, *J*_HH_ *=* 7.5, 1.9 Hz, *J*_HP_ = 1.9 Hz, 1H), 2.90 (d, *J*_HP_ *=* 19.6 Hz, 2H). ^13^C NMR (101 MHz, D_2_O) δ 168.04, 162.49, 140.33 (d, *J*_CP_ *=* 7.9 Hz), 137.17, 132.89, 129.66 (d, *J*_CP_ *=* 5.2 Hz), 129.14, 128.04, 127.58 (d, *J*_CP_ *=* 5.6 Hz), 126.67, 126.60, 124.67, 123.41, 37.24 (d, *J*_CP_ *=* 121.7 Hz). ^31^P NMR (162 MHz, D_2_O) δ 17.71. ^77^Se NMR (76 MHz, D_2_O) δ 996.60. HRMS (ESI) *m*/*z* calculated for C_14_H_12_NO_4_PSe+H^+^ 369.9747, found 369.9730.

##### 4-(3-Oxobenzo­[*d*]­[1,2]­selenazol-2­(3*H*)-yl)­benzylphosphonic acid (**1h**)

White solid, yield 53%, mp >260 °C. ^1^H NMR (400 MHz, D_2_O) δ 7.81–7.76 (m, 2H), 7.64–7.60 (m, 1H), 7.45–7.33 (m, 5H), 2.89 (d, *J*_HP_ *=* 19.8 Hz, 2H). ^13^C NMR (101 MHz, D_2_O) δ 167.73, 162.07, 139.93, 138.84, 134.57, 132.71, 130.54 (d, *J*_CP_ *=* 5.5 Hz), 127.87, 126.56, 125.99, 124.50, 37.16 (d, *J*_CP_ *=* 121.9 Hz). ^31^P NMR (162 MHz, D_2_O) δ 17.82. ^77^Se NMR (76 MHz, D_2_O) δ 996.14. HRMS (ESI) *m*/*z* calculated for C_14_H_12_NO_4_PSe+H^+^ 369.9747, found 369.9730.

#### Monoethyl Esters of Phosphonic Acids (**15a and 15b**)

To diethyl ester **14a** or **14d** (1.0 mmol) dissolved in methanol (2 mL) was added 2 M aqueous solution of NaOH (2 mL) was added. The mixture was stirred for 2 h, and volatiles were evaporated *in vacuo*. The residue was purified by reverse-phase HPLC chromatography (using a gradient of acetonitrile/water, 10/90 → 90/10 vv + 0.05% TFA).

##### Monoethyl Ester of 2-(3-oxobenzo­[*d*]­[1,2]­selenazol-2­(3*H*)-yl)­ethylphosphonic acid (**15a**)

White solid, yield 43%, mp 210–211 °C. ^1^H NMR (400 MHz, D_2_O) δ 7.80 (dd, *J*_HH_ *=* 7.9, 1.2 Hz, 1H), 7.60 (dd, *J*_HH_ *=* 7.7, 1.5 Hz, 1H), 7.36 (ddd, *J*_HH_ *=* 8.8, 7.9, 1.5 Hz, 1H), 7.31 (ddd, *J*_HH_ *=* 8.8, 7.7, 1.2 Hz, 1H), 3.86 (quin, *J*_HH_ *∼ J*_HP_ *∼* 7.0 Hz, 2H), 3.66–3.59 (m, 2H), 2.00–1.92 (m, 2H), 1.16 (t, *J*_HH_ *=* 7.1 Hz, 3H). ^13^C NMR (101 MHz, D_2_O) δ 169.54, 135.60, 132.08, 130.84, 128.66, 127.56, 126.58, 60.78 (d, *J*_CP_ *=* 6.0 Hz), 37.95 (d, *J*_CP_ *=* 4.0 Hz), 26.70 (d, *J*_CP_ *=* 131.6 Hz), 15.89 (d, *J*_CP_ *=* 6.6 Hz). ^31^P NMR (162 MHz, D_2_O) δ 24.10. ^77^Se NMR (76 MHz, D_2_O) δ 925.56. HRMS (ESI) *m*/*z* calculated for C_11_H_14_NO_4_PSe+H^+^ 335.9904, found 335.9968.

##### Monoethyl Ester of 5-(3-oxobenzo­[*d*]­[1,2]­selenazol-2­(3*H*)-yl)­pentylphosphonic acid (**15d**)

(P2.12.5) White solid, yield 48%, mp 182–183 °C. ^1^H NMR (400 MHz, D_2_O) δ 7.75 (dd, *J*_HH_ *=* 7.9, 1.2 Hz, 1H), 7.61 (dd, *J*_HH_ *=* 7.6, 1.6 Hz, 1H), 7.33 (ddd, *J*_HH_ *=* 8.9, 7.9, 1.6 Hz, 1H), 7.28 (ddd, *J*_HH_ *=* 8.9, 7.6, 1.2 Hz, 1H), 3.83–3.72 (m, 2H), 3.47 (t, *J*_HH_ *=* 6.9 Hz, 2H), 1.60 (quin, *J*_HH_ *∼* 7.0 Hz, 2H), 1.55–1.42 (m, 4H), 1.42–1.33 (m, 2H), 1.14 (t, *J*_HH_ *=* 7.1 Hz, 3H). ^13^C NMR (101 MHz, D_2_O) δ 169.42, 135.89, 131.99, 130.81, 128.40, 127.53, 126.61, 60.37 (d, *J*_CP_ *=* 5.7 Hz), 42.49, 28.76, 27.41 (d, *J*_CP_ *=* 17.2 Hz), 25,99 (d, *J*_CP_ *=* 134.1 Hz), 22.58 (d, *J*_CP_ *=* 5.2 Hz), 15.97 (d, *J*_CP_ *=* 6.3 Hz). ^31^P NMR (162 MHz, D_2_O) δ 29.26. ^77^Se NMR (76 MHz, D_2_O) δ 917.83. HRMS (ESI) *m*/*z* calculated for C_14_H_20_NO_4_PSe+H^+^ 378.0373, found 378.0499.

## Supplementary Material











## Notes

Experimental methods for the synthesis of key intermediates, diethyl aminophosphonates **5a**–**l**, with detailed characterization; methods for measuring ureolysis inhibition, antimicrobial activity, and cytotoxicity; molecular modeling protocol, with the interaction mode of diethyl phosphonates **14a**, **14f**, and **14l** and phosphonic acid **1a**, **1d**, and **1f** with *H. pylori* urease; and ^1^H, ^13^C, ^31^P, and ^77^Se NMR spectra of the final compounds **14a**–**l**, **1a**–**h**, **15a**, and **15b**, as well as selected analytical HPLC traces (PDF)
Crystallographic data for the structure of compound **1a** (CCDC 2323717) (CIF)
Crystallographic data for the structure of compound **1c** (CCDC 2323716) (CIF)
Molecular formula strings and the associated biochemical and biological data (CSV)
Ligand-bound docking models of diethyl phosphonates **14a**, **14f**, and **14l** and phosphonic acids **1a**, **1d**, and **1f** in the active sites of *S. pasteurii* urease, depicted in Figures 4 and 5, and *H. pylori* urease, depicted in Figures S3 and S4 (ZIP)
