# Supplementary material for: Synthesis of Phosphonate Derivatives of Benzisoselenazolones and Their Remarkable Antiureolytic Activity in Helicobacter pylori Cells
Source: J Med Chem. 2025 Sep 30;68(19):20324–39. doi: 10.1021/acs.jmedchem.5c01385 (PMC12516687; doi:10.1021/acs.jmedchem.5c01385)
Supplement: Supplementary file 1 [file jm5c01385_si_001.pdf]

Supporting Information

Synthesis of Phosphonate Derivatives of Benzisoselenazolones and Their Remarkable Antiureolytic Activity in *Helicobacter pylori* Cells

Marta Grabarek,[1] Wojciech Tabor,[1] Paweł Krzyżek,[2] Julia Bąkowicz,[3] Agnieszka Grabowiecka,[1] Łukasz Berlicki[1] and Artur Mucha[1]*

[1] Department of Bioorganic Chemistry, Faculty of Chemistry, Wrocław University of Science and Technology, Wybrzeże Wyspiańskiego 27, 50-370 Wrocław, Poland

[2] Department of Microbiology, Faculty of Medicine, Wrocław Medical University, Chałubińskiego 4, 50-368 Wrocław, Poland

[3] Institute of Advanced Materials, Faculty of Chemistry, Wrocław University of Science and Technology, Wybrzeże Wyspiańskiego 27, 50-370 Wrocław, Poland

Email: artur.mucha@pwr.edu.pl

Contents:

S1. Chemistry S2

S1.1. Diethyl ω-aminoalkylphosphonates S2

S1.2. Diethyl aminophenylphosphonates S4

S1.3. Diethyl aminophenylalkylphosphonates S5

S1.4. Diethyl (aminomethyl)benzylphosphonates S8

S2. Structural Studies S10

S3. Biological Studies S11

S3.1. Inhibition of *S. pasteurii* urease and *H. pylori* ureolysis S11

S3.2. Antimicrobial activity and combination of drugs against *H. pylori* S12

S3.3. Antiproliferative activity S13

S4. Molecular Modeling S14

S5. References S17

S6. NMR Spectra S20

S7. Representative HPLC Analyses S53

S1. Chemistry

S1.1. Diethyl ω-aminoalkylphosphonates (A)

Diethyl ω-aminoalkylphosphonates **5a-d** (n = 2-5) were obtained from α,ω-dibromoalkanes **2a-d** in a three-step synthetic route that involved Gabriel synthesis and Arbuzov reaction.[S1,S2] First, dibromoalkanes were monosubstituted with phthalimide to provide *N*-(ω-bromoalkyl)phthalimides **3a-d**, then the latter with triethyl phosphite in a microwave-induced reaction. Hydrazinolysis of the resulting diethyl ω-phthalimidoalkylphosphonates **4a-d** completed the reaction sequence.

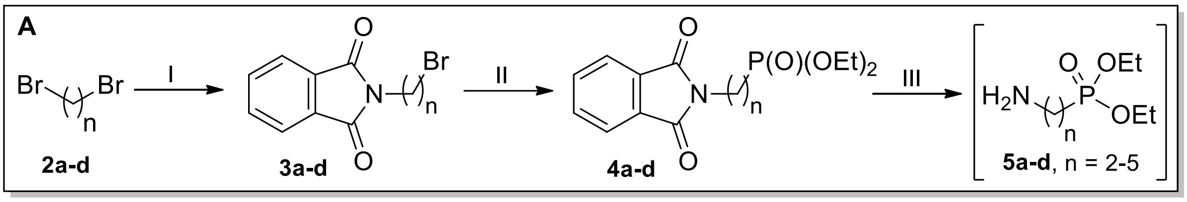

S1.1.1. *N*-(ω-Bromoalkyl)phthalimides (3a-d). General procedure I

A mixture of an α,ω-dibromoalkane **2a-d** (2 eq., typically 0.15 mol) and potassium phthalimide (1 eq.) in DMF (50 mL) was heated at 100°C for 8 h. The reaction mixture was then cooled to room temperature, the separated solid was filtered off, and the volatile components of the filtrate were evaporated *in vacuo*. The residue was dissolved in dichloromethane (200 mL) and the organic phase was washed with water (2 × 50 mL) and brine (50 mL) and dried over anhydrous sodium sulfate. The drying agent was filtered off and the solvent was evaporated *in vacuo*. The residue was purified by flash chromatography (using gradient hexane/ethyl acetate, 90/10 → 0/100 vv).

***N*-(2-Bromoethyl)phthalimide (3a).** Colorless crystals, yield 93%, mp 83-85°C. ^{1}H NMR (400 MHz, CD_3OD) δ 7.91 – 7.86 (m, 2H), 7.85 – 7.80 (m, 2H), 4.07 (t, *J* = 6.6 Hz, 2H), 3.68 (t, *J* = 6.6 Hz, 2H). ^{13}C NMR (101 MHz, CD_3OD) δ 169.30, 135.56, 133.20, 124.30, 40.44, 29.26. HRMS (ESI) *m/z* calculated for $C_{10}H_8BrNO_2+H^+$ 253.9817, found 253.9825. CAS Registry Number: 574-98-1.

***N*-(3-Bromopropyl)phthalimide (3b).** Colorless crystals, yield 92%, mp 70-72°C. ^{1}H NMR (400 MHz, CD_3OD) δ 7.88 – 7.83 (m, 2H), 7.83 – 7.77 (m, 2H), 3,82 (t, *J* = 6.9 Hz, 2H), 3.47 (t, *J* = 6.6 Hz, 2H), 2.22 (quin, *J* = 6.6 Hz, 2H). ^{13}C NMR (101 MHz, CD_3OD) δ 169.78, 135.37, 133.41, 124.13, 37.64, 32.72, 30.91. HRMS (ESI) *m/z* calculated for $C_{11}H_{10}BrNO_2+H^+$ 267.9973, found 267.9966. CAS Registry Number: 5460-29-7.

***N*-(4-Bromobutyl)phthalimide (3c).** Colorless crystals, yield 90%, mp 79-81°C. ^{1}H NMR (400 MHz, CD_3OD) δ 7.88 – 7.83 (m, 2H), 7.83 – 7.77 (m, 2H), 3.70 (t, *J* = 6.6 Hz, 2H), 3,49 (t, *J* = 6.4 Hz, 2H), 1.85 (m, 4H). ^{13}C NMR (101 MHz, CD_3OD) δ 169.85, 135.39, 133.36, 124.12, 37.83, 33.56, 31.14, 28.17. HRMS (ESI) *m/z* calculated for $C_{12}H_{12}BrNO_2$+H^+ 282.0130, found 282.0132. CAS Registry Number: 5394-18-3.

***N*-(5-Bromopentyl)phthalimide (3d).** Colorless crystals, yield 89%, mp 62-63°C. ^{1}H NMR (400 MHz, CD_3OD) δ 7.87 – 7.82 (m, 2H), 7.81 – 7.77 (m, 2H), 3.68 (t, *J* = 7.1 Hz, 2H), 3.44 (t, *J* = 6.7 Hz, 2H), 1.89 (m, 2H), 1.69 (m, 2H), 1.49 (m, 2H). ^{13}C NMR (101 MHz, CD_3OD) δ 169.84, 135.34, 133.37, 124.08, 38.50, 34.03, 33.34, 28.61, 26.35. HRMS (ESI) *m/z* calculated for $C_{13}H_{14}BrNO_2$+H^+ 296.0286, found 296.0265. CAS Registry Number: 954-81-4.

S1.1.2. Diethyl ω-phthalimidoalkylphosphonates (4a-d). General procedure II

A mixture of an *N*-(ω-bromoalkyl)phthalimide **3a-d** (1 eq.) and triethyl phosphite (2 eq.) was heated in a microwave oven at 140 °C for 2 h. After cooling to room temperature and evaporation of volatiles *in vacuo*, the oily residue was purified by flash chromatography (using gradient hexane/ethyl acetate, 20/80 → 0/100 vv).

Diethyl 2-phthalimidoethylphosphonate (4a).[S3,S4] Colorless oil, yield 96%. ^{1}H NMR (400 MHz, CD_3OD) δ 7.89 – 7.84 (m, 2H), 7.84 – 7.77 (m, 2H), 4.08 (m, 4H), 3.94 (dt, *J* = 14.5, 7.3 Hz, 2H), 2.28 (dt, *J* = 18.2, 7.3 Hz, 2H), 1.27 (t, *J* = 7.1 Hz, 6H). ^{13}C NMR (101 MHz, CD_3OD) δ 169.28, 135.43, 133.44, 124.19, 63.51 (d, *J* = 6.6 Hz), 32.86 (d, *J* = 3.7 Hz), 24.74 (d, *J* = 140.8 Hz), 16.57 (d, *J* = 6.0 Hz). ^{31}P NMR (162 MHz, CD_3OD) δ 29.83. HRMS (ESI) *m/z* calculated for $C_{14}H_{18}NO_5P$+H^+ 312.1001, found 312.0964. CAS Registry Number: 62514-90-3.

Diethyl 3-phthalimidopropylphosphonate (4b).[S3-S5] Colorless oil, yield 96%. ^{1}H NMR (400 MHz, CD_3OD) δ 7.88 – 7.82 (m, 2H), 7.82 – 7.77 (m, 2H), 4.08 (m, 4H), 3.72 (t, *J* = 7.1 Hz, 2H), 2.02 – 1.78 (m, 4H), 1.31 (t, *J* = 7.1 Hz, 6H). ^{13}C NMR (101 MHz, CD_3OD) δ 169.79, 135.40, 133.33, 124.15, 63.33 (d, *J* = 6.6 Hz), 39.06 (d, *J* = 19.0 Hz), 23.48 (d, *J* = 139.3 Hz), 22.75 (d, *J* = 1.7 Hz), 16.69 (d, *J* = 6.0 Hz). ^{31}P NMR (162 MHz, CD_3OD) δ 32.88. HRMS (ESI) *m/z* calculated for $C_{15}H_{20}NO_5P$+H^+ 326.1158, found 326.1157. CAS Registry Number: 107257-50-1.

Diethyl 4-phthalimidobutylphosphonate (4c).[S3,S5,S6] Colorless oil, yield 89%. ^{1}H NMR (400 MHz, CD_3OD) δ 7.87 – 7.82 (m, 2H), 7.82 – 7.78 (m, 2H), 4.07 (m, 4H), 3.70 (t, *J* = 6.7 Hz, 2H), 1.93 – 1.75 (m, 4H), 1.61 (m, 2H), 1.29 (t, *J* = 7.0 Hz, 6H). ^{13}C NMR (101 MHz, CD_3OD)

δ 169.84, 135.39, 133.34, 124.11, 63.16 (d, J = 6.6 Hz), 37.95, 30.04 (d, J = 16.1 Hz), 25.11 (d, J = 140.5 Hz), 20.71 (d, J = 5.2 Hz), 16.69 (d, J = 6.0 Hz). ^{31}P NMR (162 MHz, CD_3OD) δ 33.60. HRMS (ESI) *m/z* calculated for $C_{16}H_{22}NO_5P+H^+$ 340.1314, found 340.1321. CAS Registry Number: 86791-02-8.

Diethyl 5-phthalimidopentylphosphonate (4d).[S5,S6] Colorless oil, yield 95%. ^{1}H NMR (400 MHz, CD_3OD) δ 7.86 – 7.81 (m, 2H), 7.81 – 7.76 (m, 2H), 4.07 (m, 4H), 3.67 (t, J = 7.0 Hz, 2H), 1.85 – 1.56 (m, 6H), 1.45 (m, 2H), 1.30 (t, J = 7.0 Hz, 6H). ^{13}C NMR (101 MHz, CD_3OD) δ 169.82, 135.34, 133.35, 124.07, 63.10 (d, J = 6.6 Hz), 38.44, 28.97, 28.52 (d, J = 16.1 Hz), 25.54 (d, J = 140.2 Hz), 22.95 (d, J = 5.2 Hz), 16.71 (d, J = 6.0 Hz). ^{31}P NMR (162 MHz, CD_3OD) δ 33.93. HRMS (ESI) *m/z* calculated for $C_{17}H_{24}NO_5P+H^+$ 354.1470, found 354.1458. CAS Registry Number: 145119-11-5.

S1.1.3. Diethyl ω-aminoalkylphosphonates (5a-d). General procedure III

Hydrazine monohydrate (3 eq.) was added dropwise to a solution of a phthalimide **4a-d** (1 eq., typically 5 mmol) in ethanol (100 mL) at room temperature. After stirring for 12 h, the resulting precipitate of phthalic hydrazide was filtered off and the volatile components of the filtrate evaporated *in vacuo*. The residue was dissolved in ethyl acetate (100 mL), the hydrazide was filtered off again and the solvent evaporated *in vacuo*. The crude residues of diethyl ω-aminoalkylphosphonates **5a-d** were used directly in the aminolysis of 2-(chloroseleno)benzoyl chloride (**12**) without further purification. CAS Registry Numbers: **5a**,[S4,S5] 41468-36-4; **5b**,[S4,S5] 4402-24-8; **5c**,[S5] 53253-54-6; **5d**,[S5] 53253-55-7.

S1.2. Diethyl aminophenylphosphonates (B)

Diethyl aminophenylphosphonates **5e** and **5f** were obtained by the Hirao reaction, palladium-catalyzed coupling of a bromo/iodoaniline (**6a** or **6b**) with a phosphorus component (diethyl phosphite[S7] or triethyl phosphite[S8]).

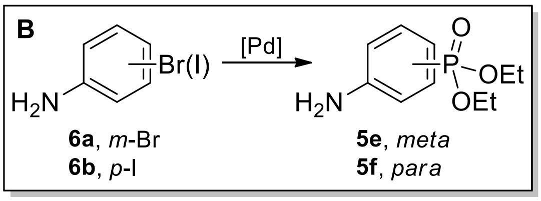

Diethyl 3-aminophenylphosphonates (5e).[S9] 3-Bromoaniline (**6a**, 35 mmol, 6.0 g), diethyl phosphite (44 mmol, 6.0 g), triphenylphosphine (5.9 mmol, 1.6 g), palladium(II) acetate (2.4 mmol, 0.55 g) and triethylamine (87 mmol, 8.8 g) were refluxed in ethanol (25 mL) under argon

atmosphere for 24 h. The reaction mixture was then cooled to room temperature, the separated solid was filtered off, and the volatile components of the filtrate were evaporated *in vacuo*. The oily residue was purified by flash chromatography (using gradient hexane/ethyl acetate, 20/80 → 0/100 vv). Orange oil, yield 80%. ^{1}H NMR (400 MHz, CD_3OD) δ 7.23 (ddd, *J* = 8.1, 7.3, 5.3 Hz, 1H), 7.08 (ddd, *J* = 14.4, 1.7, 1.6 Hz, 1H), 7.02 (dddd, *J* = 13.2, 7.4, 1.2, 1.2 Hz, 1H), 6.91 (dddd, *J* = 8.1, 2.3, 1.1, 1.1 Hz, 1H), 4.07 (m, 4H), 1.31 (t, *J* = 7.1 Hz, 6H). ^{13}C NMR (101 MHz, CD_3OD) δ 149.67 (d, *J* = 18.1 Hz), 130.55 (d, *J* = 17.5 Hz), 128.84 (d, *J* = 187.3 Hz), 121.27 (d, *J* = 9.8 Hz), 120.31 (d, *J* = 3.4 Hz), 118.38 (d, *J* = 11.5 Hz), 63.59 (d, *J* = 5.7 Hz), 16.57 (d, *J* = 6.3 Hz). ^{31}P NMR (162 MHz, CD_3OD) δ 21.26. HRMS (ESI) *m/z* calculated for $C_{10}H_{16}NO_3P+H^+$ 230.0946, found 230.0862. CAS Registry Number: 89277-85-0.

Diethyl 4-aminophenylphosphonates (5f).[S8] 4-Iodoaniline (**6b**, 40 mmol, 8.7 g), triethyl phosphite (80 mmol, 13.3 g), tri-*n*-propylamine (80 mmol, 11.5 g), tetra-*n*-butylammonium bromide (6.0 mmol, 1.9 g) and palladium(II) chloride (2.0 mmol, 0.35 g) were refluxed in water (100 mL) for 3 h. After cooling to room temperature, the product was extracted with ethyl acetate (3 × 50 mL). The organic phase was then washed with brine (20 mL) and dried over anhydrous sodium sulfate. The drying agent was filtered off and the solvent was evaporated *in vacuo*. The residue was crystallized from ethyl acetate. Cream crystals, yield 53%, mp 117-118°C. ^{1}H NMR (400 MHz, CD_3OD) δ 7.44 (dd, *J* = 12.8, 8.6 Hz, 2H), 6.71 (dd, *J* = 8.6, 3.9 Hz, 2H), 4.02 (m, 4H), 1.29 (t, *J* = 7.1 Hz, 6H). ^{13}C NMR (101 MHz, CD_3OD) δ 154.39 (d, *J* = 2.9 Hz), 134.40 (d, *J* = 11.8 Hz), 114.77 (d, *J* = 16.1 Hz), 112.96 (d, *J* = 200.8 Hz), 63.18 (d, *J* = 5.5 Hz), 16.58 (d, *J* = 6.6 Hz). ^{31}P NMR (162 MHz, CD_3OD) δ 23.49. HRMS (ESI) *m/z* calculated for $C_{10}H_{16}NO_3P+H^+$ 230.0946, found 230.0918. CAS Registry Number: 42822-57-1.

S1.3. Diethyl aminophenylalkylphosphonates (C)

Diethyl aminophenylalkylphosphonates **5g-j** were obtained from nitrophenylalkyl bromides **7a-d** in a microwave-induced Arbuzov reaction (according to General procedure II) followed by a classical reduction of the nitro group of intermediate diethyl nitrophenylalkylphosphonates **8a-d** with tin(II) chloride.[S10]

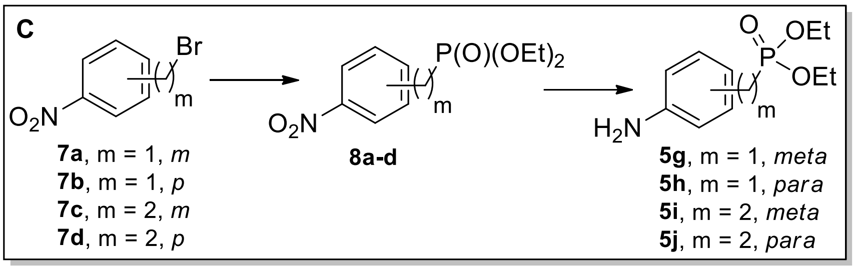

S1.3.1. Diethyl nitrophenylalkylphosphonates (8a-d)

Diethyl 3-nitrobenzylphosphonate (8a).[S9] Orange oil, yield 91%. ^{1}H NMR (400 MHz, CD_3OD) δ 8.20 (dd, *J* = 2.3, 2.3 Hz, 1H), 8.12 (dddd, *J* = 8.3, 2.3, 2.3 1.1 Hz, 1H), 7.70 (dddd, *J* = 7.6, 2.6, 1.8, 1.1 Hz, 1H), 7.55 (td, *J* = 8.3, 1.2 Hz, 1H), 4.06 (m, 4H), 3.40 (d, *J* = 21.9 Hz, 2H), 1.25 (t, *J* = 7.1 Hz, 6H). ^{13}C NMR (101 MHz, CD_3OD) δ 149.63, 137.36 (d, *J* = 6.3 Hz), 135.56 (d, *J* = 9.5 Hz), 130.72 (d, *J* = 2.9 Hz), 125.68 (d, *J* = 6.6 Hz), 122.94 (d, *J* = 3.4 Hz), 63.92 (d, *J* = 6.9 Hz), 33.18 (d, *J* = 137.9 Hz), 16.63 (d, *J* = 6.0 Hz). ^{31}P NMR (162 MHz, CD_3OD) δ 26.73. HRMS (ESI) *m/z* calculated for $C_{11}H_{16}NO_5P+H^+$ 274.0844, found 274.0818. CAS Registry Number: 104097-04-3.

Diethyl 4-nitrobenzylphosphonate (8b).[S11] Orange oil, yield 84%. ^{1}H NMR (400 MHz, CD_3OD) δ 8.20 (dd, *J* = 8.9, 1.1 Hz, 2H), 7.56 (dd, *J* = 8.9, 2.6 Hz, 2H), 4.08 (m, 4H), 3.43 (d, *J* = 22.5 Hz, 2H), 1.27 (t, *J* = 7.0 Hz, 6H). ^{13}C NMR (101 MHz, CD_3OD) δ 148.45 (d, *J* = 4.0 Hz), 141.16 (d, *J* = 9.5 Hz), 132.12 (d, *J* = 6.3 Hz), 124.51 (d, *J* = 3.2 Hz), 63.95 (d, *J* = 6.9 Hz), 33.65 (d, *J* = 137.0 Hz), 16.63 (d, *J* = 6.0 Hz). ^{31}P NMR (162 MHz, CD_3OD) δ 26.34. HRMS (ESI) *m/z* calculated for $C_{11}H_{16}NO_5P+H^+$ 274.0844, found 274.0846. CAS Registry Number: 2609-49-6.

Diethyl 2-(3-nitrophenyl)ethylphosphonate (8c). Orange oil, yield 73%. ^{1}H NMR (400 MHz, CD_3OD) δ 8.16 (dd, *J* = 2.1, 2.1 Hz, 1H), 8.09 (ddd, *J* = 8.1, 2.3, 1.1 Hz, 1H), 7.69 (m, 1H), 7.55 (t, *J* = 7.9 Hz, 1H), 4.08 (m, 4H), 3.03 (m, 2H), 2.20 (m, 2H), 1.29 (t, *J* = 7.1 Hz, 6H). ^{13}C NMR (101 MHz, CD_3OD) δ 149.78, 144.35 (d, *J* = 14.9 Hz), 135.92, 130.77, 124.18, 122.46, 63.32 (d, *J* = 6.6 Hz), 29.15 (d, *J* = 4.9 Hz), 27.19 (d, *J* = 140.5 Hz), 16.66 (d, *J* = 6.3 Hz). ^{31}P NMR (162 MHz, CD_3OD) δ 31.77. HRMS (ESI) *m/z* calculated for $C_{12}H_{18}NO_5P+H^+$ 288.1001, found 288.1004.

Diethyl 2-(4-nitrophenyl)ethylphosphonate (8d).[S12] Orange oil, yield 71%. ^{1}H NMR (400 MHz, CD_3OD) δ 8.15 (d, *J* = 8.7 Hz, 2H), 7.55 – 7.48 (m, 2H), 4.09 (m, 4H), 3.00 (m, 2H), 2.16 (m, 2H), 1.30 (t, *J* = 7.0 Hz, 6H). ^{13}C NMR (101 MHz, CD_3OD) δ 150.03 (d, *J* = 15.5 Hz), 148.14, 130.52, 124.65, 63.36 (d, *J* = 6.3 Hz), 29.36 (d, *J* = 4.3 Hz), 27.06 (d, *J* = 140.5

Hz), 16.67 (d, J = 6.0 Hz). ^{31}P NMR (162 MHz, CD_3OD) δ 31,67. HRMS (ESI) *m/z* calculated for $C_{12}H_{18}NO_5P+H^+$ 288.1001, found 288.1009. CAS Registry Number: 100316-51-6.

S1.3.2. Diethyl aminophenylalkylphosphonates (5g-j)

A diethyl nitrophenylalkylphosphonate **8a-d** (1 eq., typically 10 mmol), tin(II) chloride (5 eq.) and concentrated hydrochloric acid (1 mL) were refluxed in ethanol (50 mL) under argon for 2 h. After cooling to room temperature, the volatile components were evaporated *in vacuo* and the residue was worked up with ethyl acetate (100 mL). The organic phase was then washed with 1.0 M aqueous NaOH (40 mL) and brine (20 mL), and dried over anhydrous sodium sulfate. The drying agent was filtered off and the solvent was evaporated *in vacuo*. The residue was purified by flash chromatography (using gradient ethyl acetate/methanol, 100/0 → 90/10 vv).

Diethyl 3-aminobenzylphosphonate (5g).[S9,S13] Orange oil, yield 82%. ^{1}H NMR (400 MHz, CD_3OD) δ 7.04 (dt, J = 7.7, 1.1 Hz, 1H), 6.70 – 6.58 (m, 3H), 4.02 (m, 4H), 3.10 (d, J = 21.7 Hz, 2H), 1.25 (t, J = 7.0 Hz, 6H). ^{13}C NMR (101 MHz, CD_3OD) δ 147.66 (d, J = 2.9 Hz), 131.81 (d, J = 9.2 Hz), 128.88 (d, J = 3.2 Hz), 119.37 (d, J = 6.6 Hz), 116.63 (d, J = 6.6 Hz), 113.92 (d, J = 3.7 Hz), 62.36 (d, J = 6.9 Hz), 32.47 (d, J = 137.6 Hz), 15.32 (d, J = 6.0 Hz). ^{31}P NMR (162 MHz, CD_3OD) δ 28.65. HRMS (ESI) *m/z* calculated for $C_{11}H_{18}NO_3P+H^+$ 244.1103, found 244.1073. CAS Registry Number: 104139-11-9.

Diethyl 4-aminobenzylphosphonate (5h).[S13] Cream crystals, yield 87%, mp 92-95°C. ^{1}H NMR (400 MHz, CD_3OD) δ 7.04 (dd, J = 8.5, 2.6 Hz, 2H), 6.68 (d, J = 8.2 Hz, 2H), 3.99 (m, 4H), 3.08 (d, J = 20.9 Hz, 2H), 1.24 (t, J = 7.1 Hz, 6H). ^{13}C NMR (101 MHz, CD_3OD) δ 147.83 (d, J = 3.4 Hz), 131.60 (d, J = 6.6 Hz), 121.22 (d, J = 9.8 Hz), 116.67 (d, J = 3.2 Hz), 63.58 (d, J = 7.2 Hz), 32.77 (d, J = 138.7 Hz), 16.66 (d, J = 6.0 Hz). ^{31}P NMR (162 MHz, CD_3OD) δ 29.12. HRMS (ESI) *m/z* calculated for $C_{11}H_{18}NO_3P+H^+$ 244.1103, found 244.1073. CAS Registry Number: 20074-79-7.

Diethyl 2-(3-aminophenyl)ethylphosphonate (5i).[S12] Orange oil, yield 76%. ^{1}H NMR (400 MHz, CD_3OD) δ 7.02 (t, J = 7.7 Hz, 1H), 6.63 – 6.53 (m, 3H), 4.07 (m, 4H), 2.76 (m, 2H), 2.06 (m, 2H), 1.31 (t, J = 7.0 Hz, 6H). ^{13}C NMR (101 MHz, CD_3OD) δ 147.63, 141.60 (d, J = 16.4 Hz), 128.99, 117.61, 114.91, 113.44, 61.87 (d, J = 6.6 Hz), 28.14 (d, J = 4.9 Hz), 26.56 (d, J = 138.5 Hz), 15.37 (d, J = 6.0 Hz). ^{31}P NMR (162 MHz, CD_3OD) δ 32.80. HRMS (ESI) *m/z* calculated for $C_{12}H_{20}NO_3P+H^+$ 258.1259, found 258.1269. CAS Registry Number: 1632285-28-9.

Diethyl 2-(4-aminophenyl)ethylphosphonate (5j). Orange oil, yield 72%. ^{1}H NMR (400 MHz, CD_3OD) δ 6.97 (d, *J* = 8.4 Hz, 2H), 6.68 (d, *J* = 8.4 Hz, 2H), 4.05 (m, 4H), 2.75 (m, 2H), 2.02 (m, 2H), 1.29 (t, *J* = 7.1 Hz, 6H). ^{13}C NMR (101 MHz, CD_3OD) δ 146.88, 131.64 (d, *J* = 16.4 Hz), 129.76, 116.92, 63.12 (d, *J* = 6.6 Hz), 28.62 (d, *J* = 4.6 Hz), 28.22 (d, *J* = 137.6 Hz), 16.70 (d, *J* = 6.0 Hz). ^{31}P NMR (162 MHz, CD_3OD) δ 32.96. HRMS (ESI) *m/z* calculated for $C_{12}H_{20}NO_3P+H^+$ 258.1259, found 258.1265. CAS Registry Number: 100525-35-7.

S1.4. Diethyl (aminomethyl)benzylphosphonates (D)

Diethyl (aminomethyl)benzylphosphonates **5k** and **5l** were obtained from 1,3-bis(chloromethyl)benzene (**9a**) or 1,4-bis(bromomethyl)benzene (**9b**), respectively, in a three-step synthetic route leading *via* phthalimido halides (**10a** and **10b**) and phthalimidophosphonates (**11a** and **11b**), as described for diethyl ω-aminoalkylphosphonates (General procedures I-III).

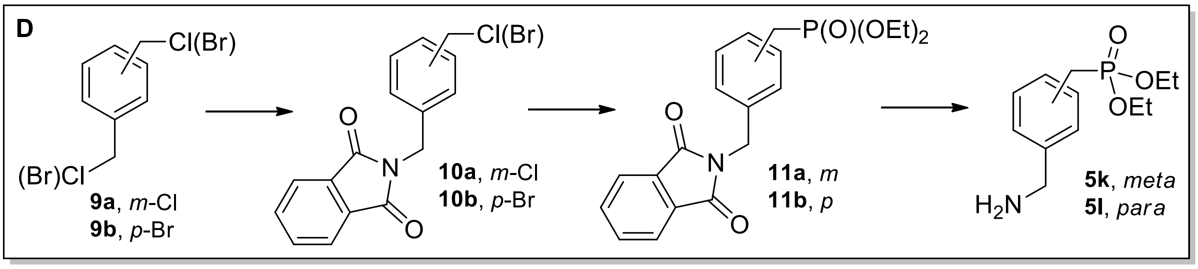

S1.4.1. *N*-(Halogenomethyl)benzylphthalimides (10a and 10b)

***N*-(3-Chloromethyl)benzylphthalimide (10a).**[S14] Colorless crystals, yield 65%, mp 128-130°C. ^{1}H NMR (400 MHz, $CDCl_3$) δ 7.85 (dd, *J* = 5.4, 3.1 Hz, 2H), 7.71 (dd, *J* = 5.4, 3.1 Hz, 2H), 7.44 (s, 1H), 7.41 – 7.37 (m, 1H), 7.33 – 7.30 (m, 2H), 4.84 (s, 2H), 4.55 (s, 2H). ^{13}C NMR (101 MHz, $CDCl_3$) δ 168.13, 138.09, 137.02, 134.20, 132.18, 129.31, 128.92, 128.86, 128.29, 123.55, 46.06, 41.46. HRMS (ESI) *m/z* calculated for $C_{16}H_{12}BrNO_2+H^+$ 330.0130, found 330.0115. CAS Registry Number: 221623-69-4.

***N*-(4-Bromomethyl)benzylphthalimide (10b).**[S15] Colorless crystals, yield 65%, mp 143-144°C. ^{1}H NMR (400 MHz, $CDCl_3$) δ 7.84 (dd, *J* = 5.3, 3.1 Hz, 2H), 7.71 (dd, *J* = 5.5, 3.1 Hz, 2H), 7.41 (d, *J* = 8.2 Hz, 2H), 7.34 (d, *J* = 8.2 Hz, 2H), 4.83 (s, 2H), 4.45 (s, 2H). ^{13}C NMR (101 MHz, $CDCl_3$) δ 168.11, 137.52, 136.73, 134.20, 132.18, 129.52, 129.23, 123.52, 41.31, 33.15. HRMS (ESI) *m/z* calculated for $C_{16}H_{12}BrNO_2+H^+$ 330.0130, found 330.0159. CAS Registry Number: 101367-16-2.

S1.4.2. Diethyl (phthalimidomethyl)benzylphosphonates (11a and 11b)

Diethyl 3-(phthalimidomethyl)benzylphosphonate (11a). Colorless crystals, yield 66%, mp 97-98°C. ^{1}H NMR (400 MHz, $CDCl_3$) δ 7.83 (dd, *J* = 5.4, 3.0 Hz, 2H), 7.70 (dd, *J* = 5.4, 3.0 Hz, 2H), 7.35 – 7.18 (m, 4H), 4.82 (s, 2H), 3.98 (m, 4H), 3.12 (d, *J* = 21.5 Hz, 2H), 1.20 (t, *J* = 7.1 Hz, 6H). ^{13}C NMR (101 MHz, $CDCl_3$) δ 168.11, 136.75 (d, *J* = 3.2 Hz), 134.16, 132.22 (d, *J* = 9.2 Hz), 132.20, 129.99 (d, *J* = 6.9 Hz), 129.43 (d, *J* = 6.3 Hz), 129.06 (d, *J* = 2.9 Hz), 127.25 (d, *J* = 3.4 Hz), 123.48, 62.37 (d, *J* = 6.9 Hz), 41.55, 33.74 (d, *J* = 137.9 Hz), 16.44 (d, *J* = 6.0 Hz). ^{31}P NMR (162 MHz, $CDCl_3$) δ 26.71. HRMS (ESI) *m/z* calculated for $C_{20}H_{22}NO_5P+H^+$ 388.1314, found 388.1317. CAS Registry Number: 689251-98-7.

Diethyl 4-(phthalimidomethyl)benzylphosphonate (11b). Colorless crystals, yield 69%, mp 107-109°C. ^{1}H NMR (400 MHz, $CDCl_3$) δ 7.83 (dd, *J* = 5.5, 3.1 Hz, 2H), 7.70 (dd, *J* = 5.5, 3.1 Hz, 2H), 7.36 (d, *J* = 8.1 Hz, 2H), 7.23 (dd, *J* = 8.3, 2.4 Hz, 2H), 4.81 (s, 2H), 3.99 (m, 4H), 3.10 (d, *J* = 21.7 Hz, 2H), 1.22 (t, *J* = 7.1 Hz, 6H). ^{13}C NMR (101 MHz, $CDCl_3$) δ 168.15, 135.13 (d, *J* = 4.0 Hz), 134.14, 132.22, 131.32 (d, *J* = 9.5 Hz), 130.16 (d, *J* = 6.6 Hz), 128.96 (d, *J* = 3.2 Hz), 123.47, 62.33 (d, *J* = 6.9 Hz), 41.35, 33.50 (d, *J* = 138.2 Hz), 16.47 (d, *J* = 6.0 Hz). ^{31}P NMR (162 MHz, $CDCl_3$) δ 26.85. HRMS (ESI) *m/z* calculated for $C_{20}H_{22}NO_5P+H^+$ 388.1314, found 388.1315.

S1.4.3. Diethyl (aminomethyl)benzylphosphonates (5l and 5k)

Diethyl 3-(aminomethyl)benzylphosphonate (5l). Light yellow oil, yield 77%. ^{1}H NMR (400 MHz, CD_3OD) δ 7.37 – 7.21 (m, 4H), 4.03 (m, 4H), 3.86 (s, 2H), 3.25 (d, *J* = 21.7 Hz, 2H), 1.26 (t, *J* = 7.1 Hz, 6H). ^{13}C NMR (101 MHz, CD_3OD) δ 141.66 (d, *J* = 3.3 Hz), 133.17 (d, *J* = 9.4 Hz), 130.48 (d, *J* = 6.6 Hz), 130.11 (d, *J* = 6.6 Hz), 129.93 (d, *J* = 3.2 Hz), 127.51 (d, *J* = 3.7 Hz), 63.73 (d, *J* = 6.9 Hz), 45.93, 33.56 (d, *J* = 137.8 Hz), 16.66 (d, *J* = 6.0 Hz). ^{31}P NMR (162 MHz, CD_3OD) δ 28.23. HRMS (ESI) *m/z* calculated for $C_{12}H_{20}NO_3P+H^+$ 258.1259, found 258.1249. CAS Registry Number: 689252-30-0.

Diethyl 4-(aminomethyl)benzylphosphonate (5k). Light yellow oil, yield 81%. ^{1}H NMR (400 MHz, CD_3OD) δ 7.40 – 7.30 (m, 4H), 4.03 (m, 4H), 3.92 (d, *J* = 1.8 Hz, 2H), 3.25 (d, *J* = 21.7 Hz, 2H), 1.26 (t, *J* = 7.1 Hz, 6H). ^{13}C NMR (101 MHz, CD_3OD) δ 138.23 (d, *J* = 4.0 Hz), 132.57 (d, *J* = 9.4 Hz), 131.43 (d, *J* = 6.6 Hz), 129.35 (d, *J* = 3.2 Hz), 63.70 (d, *J* = 6.9 Hz), 45.23, 33.30 (d, *J* = 137.8 Hz), 16.65 (d, *J* = 6.0 Hz). ^{31}P NMR (162 MHz, CD_3OD) δ 28.02. HRMS (ESI) *m/z* calculated for $C_{12}H_{20}NO_3P+H^+$ 258.1259, found 258.1265. CAS Registry Number: 93041-76-0.

S2. Structural Studies

X-ray diffraction data for compounds **1a** and **1c** were collected using a 4-circle diffractometer equipped with a CCD detector (graphite monochromatic, MoK*α* radiation, λ = 0.71073 Å) at room temperature. Data were processed using the CrysAlisPro software.[S16] The structures were solved by direct methods using SHELXS[S17] and refined by means of SHELXL.[S17,S18] All non-hydrogen atoms were located on a difference Fourier map and treated anisotropically. The hydrogen atoms at O3 and O4 were located on a ΔF map and refined as part of a rigid rotating group with O–H = 0.82 Å and U_{iso}(H) = 1.5 U_{eq}(O). The remaining H atoms were positioned geometrically and treated as riding with C–H = 0.93 - 0.97 Å and U_{iso}(H) = 1.2 U_{eq}(C). The most important experimental and crystallographic data for the reported structures are given in Table S1. Full details are included in the supplementary cif files. Crystallographic data for the structures reported in this paper have been deposited with the Cambridge Crystallographic Data Centre as supplementary publications nos. CCDC 2323717 and CCDC 2323716, respectively, for the crystals of **1a** and **1c**.

Table S1. Selected crystal data for compounds **1a** and **1c**.

	**1a**	**1c**
Chemical formula	$C_9H_{10}NO_4PSe$	$C_{11}H_{14}NO_4PSe$
Formula weight	306.11	334.16
T (K)	298(2)	298(2)
Wavelength (Å)	0.71073	0.71073
Crystal size (mm)	0.50 × 0.20 × 0.12	0.50 × 0.09 × 0.07
Crystal system	Triclinic	Monoclinic
Space group	$P\bar{1}$	$P2_1$/n
a (Å)	11.5980(4)	10.5804(2)
b (Å)	11.7781(4)	8.47751(18)
c (Å)	12.6266(5)	15.1112(3)
α (°)	83.664(3)	90.0
β (°)	80.214(3)	102.587(2)
γ (°)	80.653(3)	90.0
V (Å^3)	1671.07(11)	1322.83(5)
Z	6	4
D_x (Mg m^{-3})	1.825	1.678
μ (mm^{-1})	3.511	2.965
F (000)	912	672
Θ_{max} (°)	26.0	26.0
Relections collected	11554	4512
Reflections independent	6576	2603
Reflections observed	5597	2153
R_{int}	0.019	0.017
R [$F^2 > 2\sigma(F^2)$], wR [F^2], S	0.032, 0.077, 1.04	0.031, 0.070, 1.07
$\Delta\rho_{max}$, $\Delta\rho_{min}$ (e Å^{-3})	0.65, –0.35	0.52, –0.35

S3. Biological Studies

S3.1. Inhibition of *S. pasteurii* urease and *H. pylori* ureolysis

The native urease was induced in a commercially available strain of *Sporosarcina pasteurii* CCM 2056 and purified in a five-step chromatographic procedure,[S19] yielding the following kinetic parameters: K_M = 14.09 ± 1.03 mM, v_{max} = 1.49 ± 0.08 μM/s. The ureolytic activity was measured using cresol red as an indicator of pH changes caused by ammonia release.[S20] The reaction was carried out in 3 mM phosphate buffer containing urease, organoselenium inhibitors at various concentrations, 30 mM urea, and 0.01 % w/v cresol red, and was followed by the use of a TECAN Sunrise spectrometer at 570 nm in a 1.5 hour-long assay. The enzyme was the last component added to initiate the kinetic assays to ensure that there was no preincubation with the inhibitors beforehand.

As the progress curves of the inhibited reactions with no enzyme-inhibitor preincubation were nonlinear (Fig. S2 and S3), and both the initial and the steady-state velocity were inversely proportional to the concentration of the studied compounds, they were determined to be slow-binding inhibitors that work in accordance with the two-step binding mechanism, where the relatively quick formation of the enzyme-inhibition complex is followed by the limiting step of its slow conformational changes.

$$E + I \leftrightarrow EI \leftrightarrow EI^*$$

The progress curves were fitted to the following equation to calculate the apparent reaction rate constants (k_{app}):

$$P = v_s * t + \frac{v_0 - v_s}{k_{app}} * \left(1 - e^{-k_{app}*t}\right)$$

P – product concentration; t – time; v_0 – initial state velocity; v_s – steady state velocity.

k_{app} was used to calculate the k_{-4} value (reaction rate constant of $EI^* \rightarrow EI$ conformational change):

$$k_{-4} = \frac{v_s}{v_0} * k_{app}$$

$1/(k_{app}-k_{-4})$ values were then plotted against 1/[I] in order to conduct a linear fitting. k_4 (reaction rate constant of the $EI \rightarrow EI^*$ conformational change) and K_i values for the inhibitor were then calculated from the equation:

$$\frac{1}{k_{app} - k_{-4}} = \frac{K_i}{k_4} * \left(1 + \frac{[S]}{K_M}\right) * \frac{1}{[I]} + \frac{1}{k_4}$$

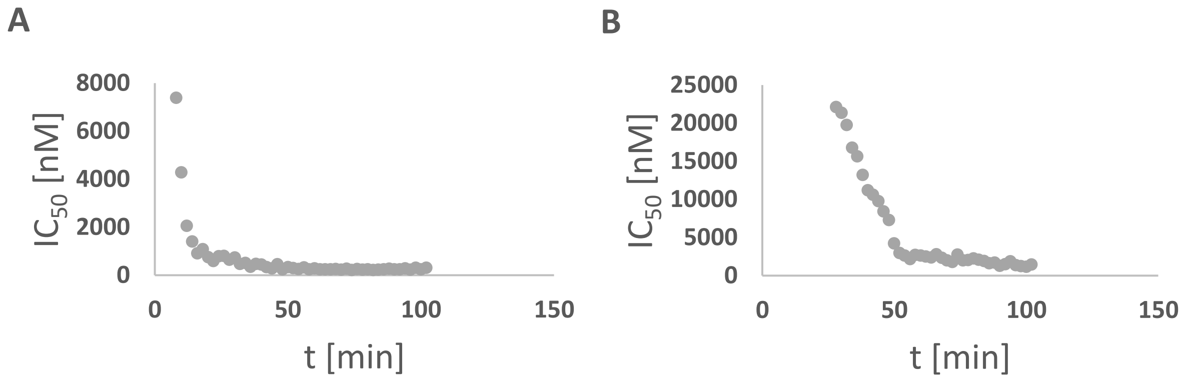

Figure S1. Shift of the IC_{50} value in time for 2-alkyl derivatives of 1,2-benzisoselenazol-3(2*H*)-one: diethyl phosphonate **14d** (panel **A**) and phosphonic acid **1d** (panel **B**).

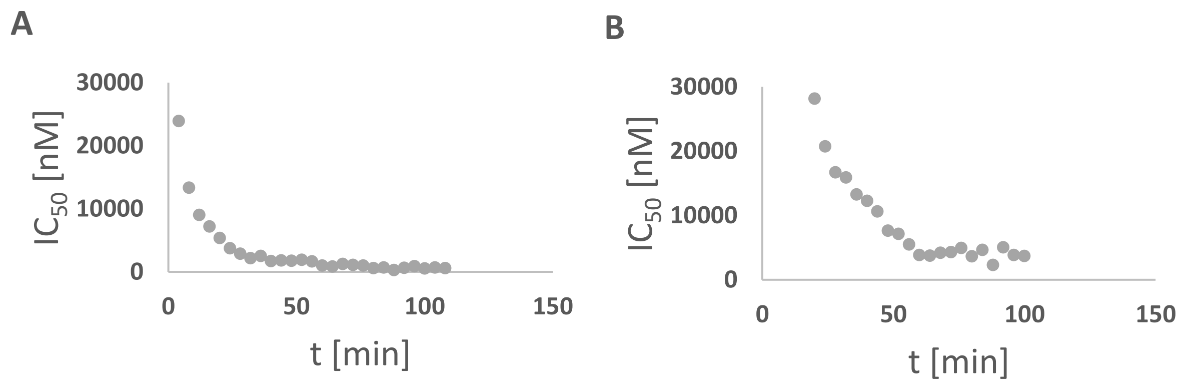

Figure S2. Shift of the IC_{50} value in time for 2-aryl derivatives of 1,2-benzisoselenazol-3(2*H*)-one: diethyl phosphonate **14g** (panel **A**) and phosphonic acid **1g** (panel **B**).

Cells of the commercially available strain of *Helicobacter pylori* Tx30a (51932, American Type Culture Collection, Manassas, VA, USA) were grown and used in the cresol red reaction to measure the activity of organoselenium inhibitors against ureolysis as previously described.[S20] Cells were washed twice with 10 mM PBS and applied as a biocatalyst in the cresol red assay similarly to the studies on purified enzyme but carried out in 10 mM PBS. The velocity in the steady state phase of the progress curves was used to calculate the IC_{50} values.

S3.2. Antimicrobial activity and combination of drugs against *H. pylori*

H. pylori Tx30a (ATCC 51932) was also used to assess the antimicrobial activity of compounds **14e-h**, **14k** and **1h** alone, and compound **14f** in combination with Amoxicillin (Merck, St. Louis, MO, USA) using the checkerboard assay as previously described.[S21] The experiments were carried out on flat-bottom 12-well microtiter plates (Bionovo, Legnica, Poland), arranged

in a 48-well panel to use the checkboard method. Concentration gradients of benzisoselenazolone **14f** and Amoxicillin ranged from 2 × MIC to 1/16 × MIC, and were applied in external wells along the x- and y-axes, respectively. The remaining wells contained varying concentrations of both compounds. Each well consisted of 1 mL of Brain Heart Infusion (Oxoid, Le Pont de Claix, France) broth with 5% foetal calf serum (Gibco, Paisley, Scotland, UK), a bacterial suspension of 10^7 CFU/mL and the desired concentration of the antimicrobial(s) tested. Following the setup, the plates were incubated for 3 days at 37 °C under microaerophilic conditions (GENbox microaer kits, BioMerrieux, Marcy l'Etoile, France), shaking at 100 rpm.

The minimum inhibitory concentration (MIC) was determined as the lowest concentration in which no microbial growth was observed.

The interactions between antimicrobials were interpreted by calculation of the fractional inhibitory concentration (FIC) index (FICI), defined as FICI = FIC_A + FIC_B (FIC_A = MIC of substance A in combination / MIC of substance A alone, FIC_B = MIC of substance B in combination / MIC of substance B alone). FICI ≤0.5 was considered as synergism; >0.5 but ≤1 as additivity, while >1 and as neutral.

S3.3. Antiproliferative activity

Antiproliferation assays were performed at the Hirszfeld Institute of Immunology and Experimental Therapy, Polish Academy of Sciences, Wrocław, Poland, similarly as previously described.[S22] Balb/3T3-L1 murine embryonic fibroblasts (ATCC) were cultured in Dulbecco's Modified Eagle Medium (High Glucose, 4.5 g/L) while HEK-293 human embryonic kidney cells (ATCC) in Modified Eagle Medium, both supplemented with fetal bovine serum (10%, v/v), L-glutamine (2 mM) and anibiotics (streptomycin, 100 μg/mL; and penicillin, 100 U/mL). Cell lines were kept in a humid atmosphere containing 5% CO_2 (v/v) at 37 °C.

Cell suspensions in their corresponding culture medium were placed in 96-well plate wells with a cell density of 10^5 cells/well (100 μL per well). After 24 hours of incubation, the compounds tested were applied at eight concentrations (ranging from 100 μM to 0.0316 μM), *cis*-platin at four concentrations (ranging from 10 μM to 0.01 μM). After 72 hours of incubation, cells were fixed with cold trichloroacetic acid (25%, w/v). After 1 h at rt, the plates were washed 5 times with tap water. The cell material was then stained with sulforhodamine B (0.4%, w/v) in acetic acid (1%, v/v), incubated for 30 minutes and washed five times with acetic acid (1%, v/v) to remove unbound dye. The dye bound to the fixed cells was dissolved with an unbuffered Tris

base (10 mM). The optical density (λ = 540 nm) was determined on a computer-interfaced BioTek Synergy H4 Hybrid Microplate Reader.

The proliferation inhibition was calculated on the basis of the crude absorbance results using the following formula:

$$Inhibition[\%] = \left(\left(\frac{A_p - A_m}{A_k - A_m} \right) \times 100 \right) - 100$$

A_p – absorbance for cells treated with compounds,

A_k – absorbance for control of cells,

A_m – absorbance for control media (cell-free).

The results were applied to the IC_{50} calculations (if possible) performed using GraphPad Prism 7.05 and the '[Inhibitor] vs. response—Variable slope (four parameters)' model. Each test was carried out in triplicate.

S4. Molecular Modeling

The crystal structures of *S. pasteurii* urease with catechol bound to Cys322 (1.50 Å resolution, PDB id 5G4H)[S23] or *H. pylori* urease complexed with acetohydroxamic acid (3.00 Å resolution, PDB id 1E9Y)[S24] were used as the starting point for the calculations. Calculations were performed using CHARMM (v. 45b2).[S25] The structure was prepared using Discovery Studio Viewer 2022 (Dassault Systemes Biovia Corp) in the following steps: (a) hydrogen atoms were automatically added assuming a pH of 7.0, (b) the protonation of amino acid residues that form active sites was manually checked and adjusted, and (c) the partial charges of all atoms were assigned using the Momany-Rone algorithm. Minimization of the inhibitor-enzyme covalent complex was performed using the CHARMM force field with the conjugate gradient minimization. Minimization was performed up to a total energy change of 0.0 or an average gradient of 0.1. Residues that did not form the active site cleft were frozen. No implicit solvent model was applied. The nonbond radius was set to 14 Å. Modeled covalent complexes of diethyl phoshononates **14a**, **14f** and **14l**, and phosphonic acids **1a**, **1d** and **1f**, with *H. pylori* urease are presented in Fig. S3 and S4.

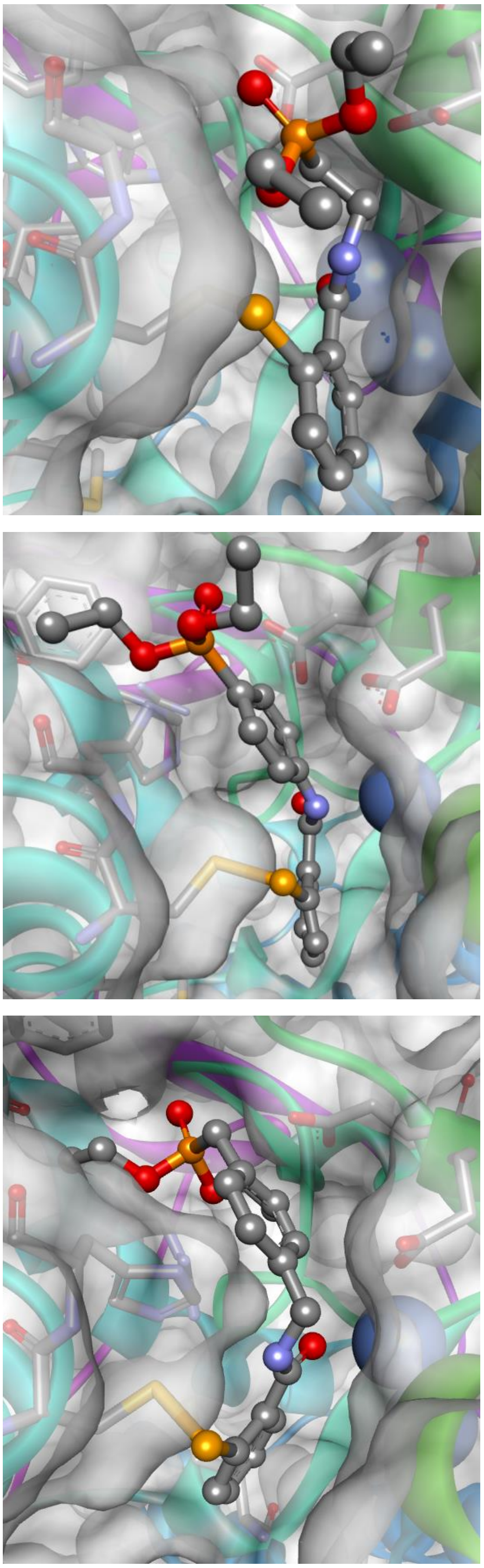

Figure S3. Modeled covalent complexes of inhibitors **14a** (panel **A**), **14f** (panel **B**) and **14l** (panel **C**) with *H. pylori* urease (PDB id 1E9Y).[S24] The enzyme is shown as a solid ribbon with a solvent-accessible surface. Nickel ions are shown as blue spheres.

A

B

C

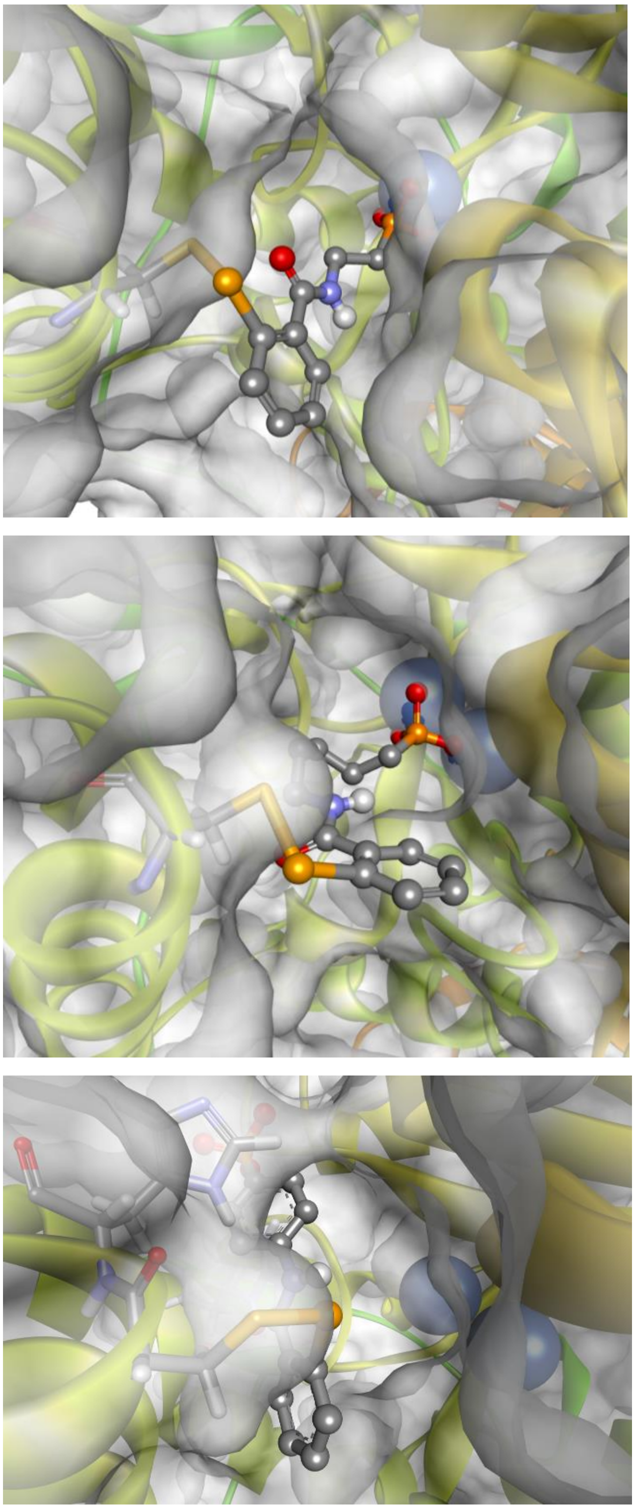

Figure S4. Modeled covalent complexes of inhibitors **1a** (panel **A**), **1d** (panel **B**) and **1f** (panel **C**) with *H. pylori* urease (PDB id 1E9Y).[S24] The enzyme is shown as a solid ribbon with a solvent-accessible surface. Nickel ions are shown as blue spheres.

S5. References

(S1) Kosolapoff, G. M. The synthesis of amino-substituted phosphonic Acids. I. J. Am. Chem. Soc. 1947, 69 (9), 2112–2113.

(S2) Yamauchi, K.; Ohtsuki, S.; Kinoshita. M. Synthesis of peptide analogs containing (2-aminoethyl)phosphonic acid (ciliatine). J. Org. Chem. 1984, 49 (7) 1158–1163.

(S3) Chun, Y. J.; Park, J. N.; Oh, G. M.; Hong, S. I.; Kim, Y. J. Synthesis of ω-phthalimidoalkylphosphonates. Synthesis 1994, (9), 909–910.

(S4) Gali, H.; Prabhu, K. R.; Karra, S. R.; Katti, K. V. Facile ring-opening reactions of phthalimides as a new strategy to synthesize amide-functionalized phosphonates, primary phosphines, and bisphosphines. J. Org. Chem. 2000, 65 (3), 676–680.

(S5) Bakó, P.; Novák, T.; Ludányi, K.; Pete, B.; Tõke, L.; Keglevich, G. D-Glucose-based azacrown ethers with a phosphonoalkyl side chain: application as enantioselective phase transfer catalysts. Tetrahedron: Asymmetry, 1999, 10 (12), 2373–2380.

(S6) Talukdar, A.; Morgunova, E.; Duan, J.; Meining, W.; Foloppe, N.; Nilsson, L.; Bacher, A.; Illarionov, B.; Fischer, M.; Ladenstein, R.; Cushman, M. Virtual screening, selection and development of a benzindolone structural scaffold for inhibition of lumazine synthase. Bioorg Med. Chem. 2010, 18 (10), 3518–3534.

(S7) Bessmertnykh, A.; Morkos Douaihy, C.; Guilard, R. Direct synthesis of amino-substituted aromatic phosphonates via palladium-catalyzed coupling of aromatic mono- and dibromides with diethyl phosphite. Chem. Lett. 2009, 38 (7), 738–739.

(S8) Iranpoor, N.; Firouzabadi, H.; Moghadam, K. R.; Motavalli, S. First reusable ligand-free palladium catalyzed C–P bond formation of aryl halides with trialkylphosphites in neat water. RSC Adv. 2014, 4 (99), 55732–55737.

(S9) Urlam, M. K.; Pireddu, R.; Ge, Y.; Zhang, X.; Sun, Y.; Lawrence, H. R.; Guida, W. C.; Sebti, S. M.; Lawrence, N. J. Development of new *N*-arylbenzamides as STAT3 dimerization inhibitors. MedChemComm. 2013, 4 (6), 932–941.

(S10) Bellamy, F.D.; Ou, K. Selective reduction of aromatic nitro compounds with stannous chloride in non acidic and non aqueous medium. Tetrahedron Lett. 1984, 25 (8), 839–842.

(S11) Mohan Naidu, K. R.; Dadapeer, E.; Reddy, C. B.; Rao, A. J.; Reddy, C. S.; Raju, C. N. Polyethylene glycol–promoted dialkyl, aryl/heteroaryl phosphonates. Synth. Commun. 2011, 41 (23), 3462–3468.

(S12) Kim, S.; Kim, C. E.; Seo, B.; Lee, P. H. In situ generation of phosphoryl alkylindiums and their synthetic application to arylalkyl phosphonates via palladium-catalyzed cross-coupling reactions. Org. Lett. 2014, 16 (21), 5552–5555.
(S13) Wydysh, E. A.; Medghalchi, S. M.; Vadlamudi, A.; Townsend, C. A. Design and synthesis of small molecule glycerol 3-phosphate acyltransferase inhibitors. J. Med. Chem. 2009, 52 (10), 3317–3327.
(S14) Kowalski, P.; Mitka, K.; Jaśkowska, J.; Duszyńska, B.; Bojarski, A. J. New arylpiperazines with flexible versus partly constrained linker as serotonin 5-HT_{1A}/5-HT_7 receptor ligands. Arch. Pharm. 2013, 346 (5), 339–348.
(S15) Briš, A.; Đud, M.; Margetić, D. Mechanochemical *N*-alkylation of imides. Beilstein. J. Org. Chem. 2017, 13, 1745–1752.
(S16) Rigaku Oxford Diffraction. CrysAlis PRO. Rigaku Oxford Diffraction Ltd, Yarnton, Oxfordshire, England, 2015.
(S17) Sheldrick, G. M. A short history of SHELX. Acta Cryst. A 2008, 64, 112–122.
(S18) Sheldrick, G. M. Crystal structure refinement with SHELXL, Acta Cryst. C 2015, 71, 3–8.
(S19) Macegoniuk, K.; Dziełak, A.; Mucha, A.; Berlicki, Ł. Bis(aminomethyl)phosphinic acid, a highly promising scaffold for the development of bacterial urease inhibitors. ACS Med. Chem. Lett. 2014, 6 (2), 146–150.
(S20) Maślanka, M.; Tabor, W.; Krzyżek, P.; Grabowiecka, A.; Berlicki, Ł.; Mucha, A. Inhibitory activity of catecholic phosphonic and phosphinic acids against *Helicobacter pylori* ureolysis. Eur. J. Med. Chem. 2023, 257, 115528.
(S21) Grabarek, M.; Tabor, W.; Krzyżek, P.; Grabowiecka, A.; Berlicki, Ł.; Mucha, A. Halogenated *N*-benzylbenzisoselenazolones efficiently inhibit helicobacter pylori ureolysis *in vitro*. ACS Med. Chem. Lett. 2025, 16 (4), 675–680.
(S22) Lenartowicz, P.; Psurski, M.; Kotynia, A.; Pieniężna, A.; Cuprych, M.; Poniatowska, K.; Brasuń, J.;. Kafarski, P. Dipeptides of *S*-substituted dehydrocysteine as artzyme building blocks: synthesis, complexing abilities and antiproliferative properties. Int. J. Mol. Sci. 2021, 22 (4), 2168.
(S23) Mazzei, L.; Cianci, M.; Musiani, F.; Lente, G.; Palombo, M.; Ciurli, S. Inactivation of urease by catechol: kinetics and structure. J. Inorg. Biochem. 2017, 166, 182–189.
(S24) Ha, N. C.; Oh, S. T.; Sung, J.; Cha, K. A.; Lee, M. H.; Oh, B. H. Supramolecular assembly and acid resistance of helicobacter pylori urease. Nat. Struct. Mol. Biol. 2001, 8 (6), 505–509.

(S25) Hwang, W.; Austin, S. L.; Blondel, A.; Boittier, E. D.; Boresch, S.; Buck, M.; Buckner, J.; Caflisch, A.; Chang, H. T.; Cheng, X.; Choi, Y. K.; Chu, J. W.; Crowley, M. F.; Cui, Q.; Damjanovic, A.; Deng, Y.; Devereux, M.; Ding, X.; Feig, M. F.; Gao, J.; Glowacki, D. R.; Gonzales, 2nd, J. E.; Hamaneh, M. B.; Harder, E. D.; Hayes, R. L.; Huang, J.; Huang, Y.; Hudson, P. S.; Im, W.; Islam, S. M.; Jiang, W.; Jones, M. R.; Käser, S.; Kearns, F. L.; Kern, N. R.; Klauda, J. B.; Lazaridis, T.; Lee, J.; Lemkul, J. A.; Liu, X.; Luo, Y.; MacKerell, Jr, A. D.; Major, D. T.; Meuwly, M.; Nam, K.; Nilsson, L.; Ovchinnikov, V.; Paci, E.; Park, S.; Pastor, R. W.; Pittman, A. R.; Post, C. B.; Prasad, S.; Pu, J.; Qi, Y.; Rathinavelan, T.; Roe, D. R.; Roux, B.; Rowley, C. N.; Shen, J.; Simmonett, A. C.; Sodt, A. J.; Töpfer, K.; Upadhyay, M.; van der Vaart, A.; Vazquez-Salazar, L. I.; Venable, R. M.; Warrensford, L. C.; Woodcock, H. L.; Wu, Y.; Brooks, 3rd, C. L.; Brooks, B. R.; Karplus, M. CHARMM at 45: Enhancements in accessibility, functionality, and speed. J. Phys. Chem. B. 2024, 128 (41), 9976–10042.

S6. NMR Spectra

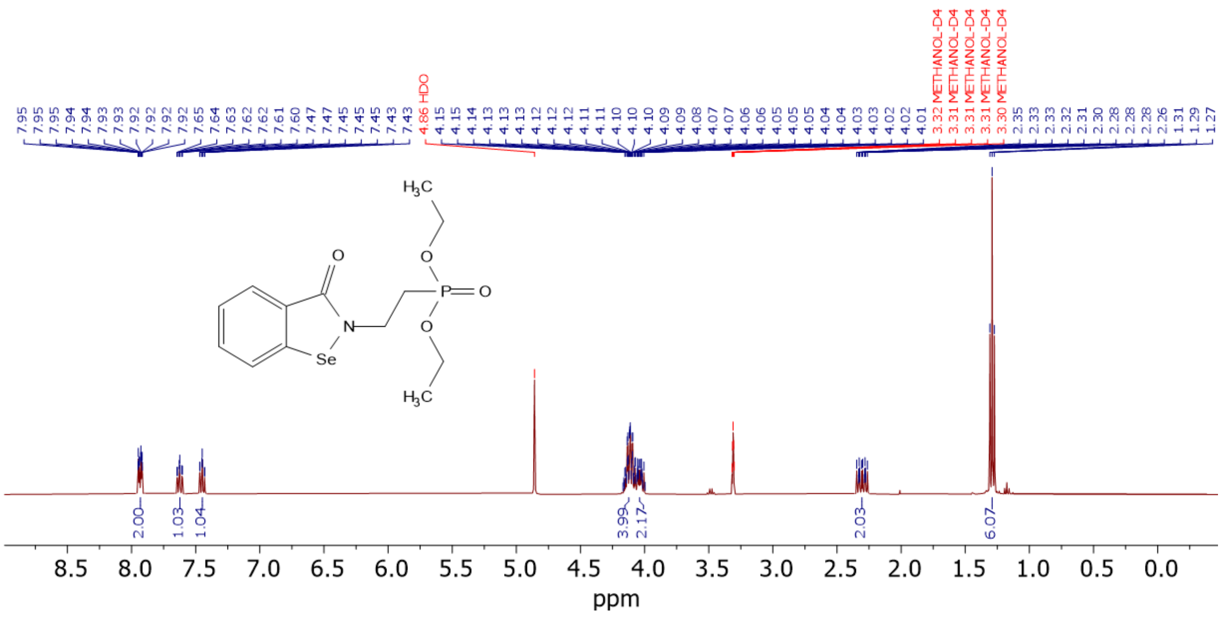

Figure S5. ^{1}H NMR spectrum of compound **14a**.

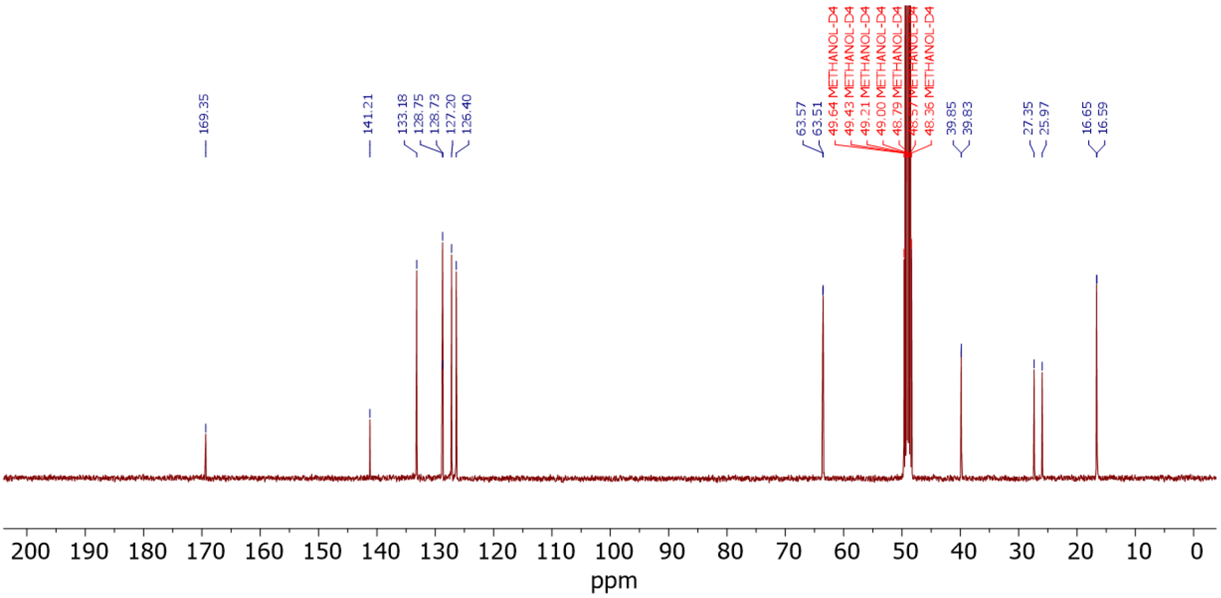

Figure S6. ^{13}C NMR spectrum of compound **14a**.

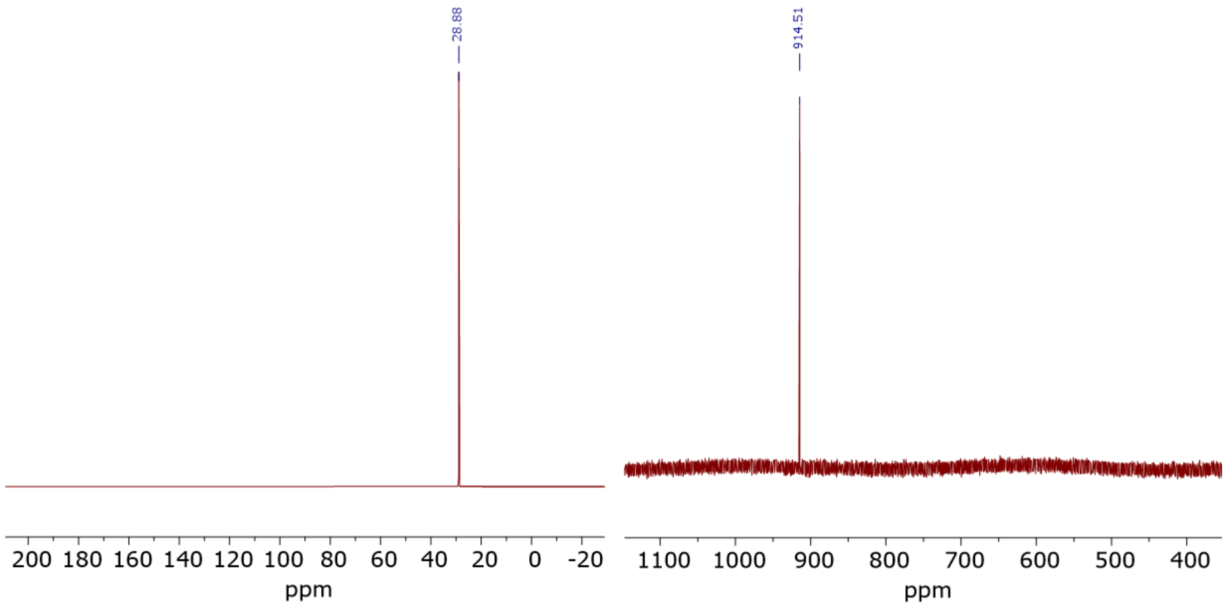

Figure S7. ^{31}P NMR (left panel) and ^{77}Se NMR (right panel) spectra of compound **14a**.

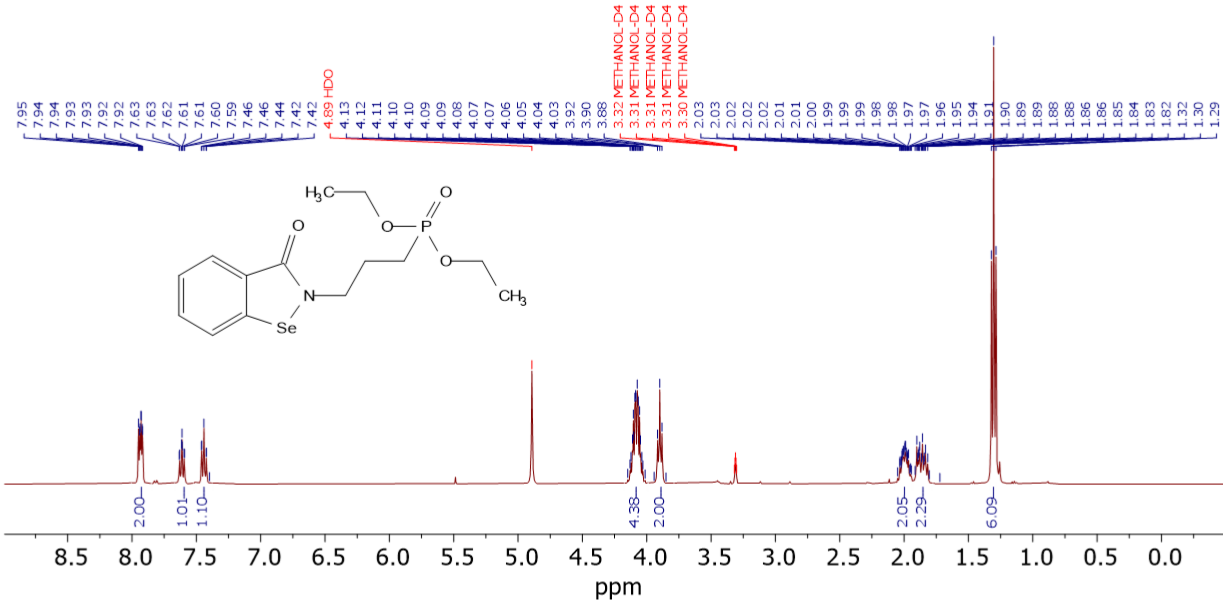

Figure S8. ^{1}H NMR spectrum of compound **14b**.

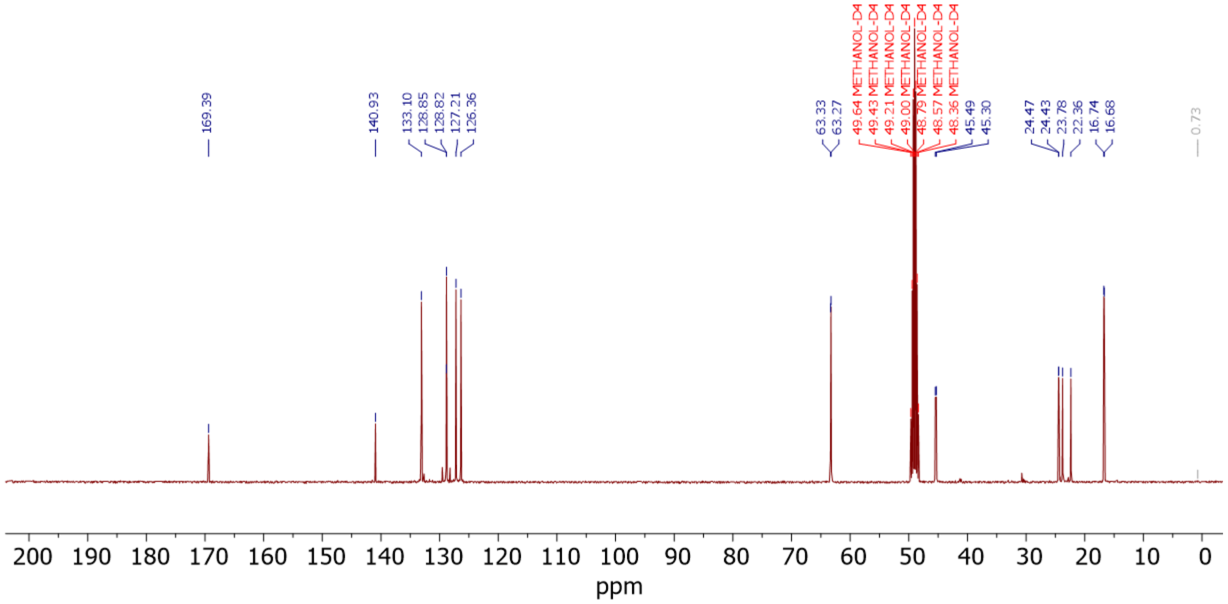

Figure S9. ^{13}C NMR spectrum of compound **14b**.

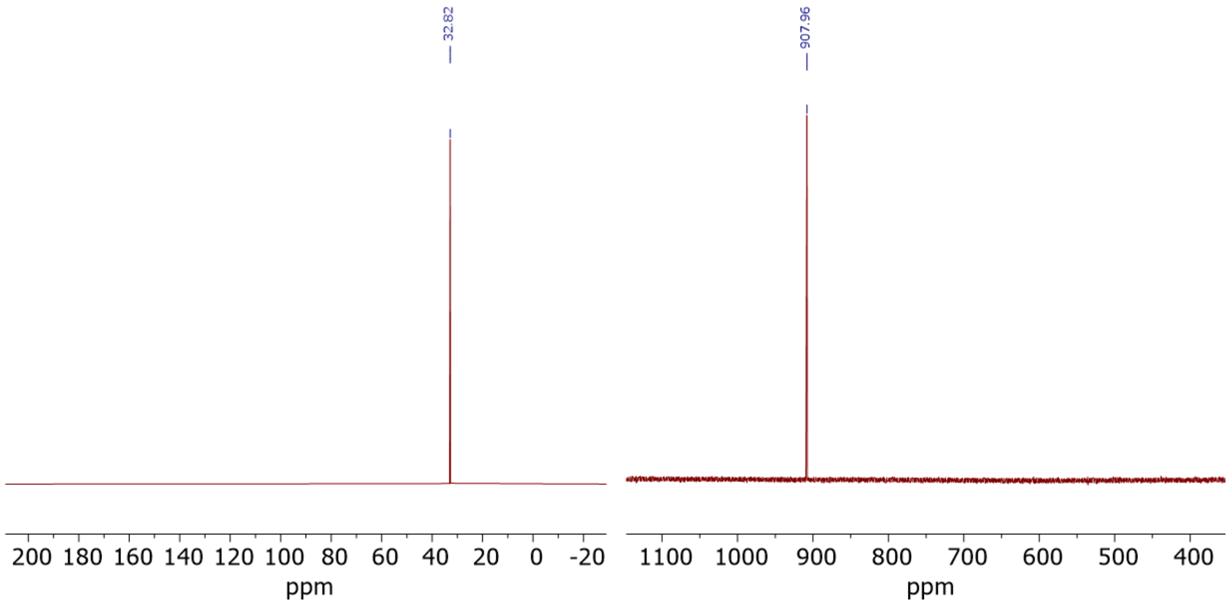

Figure S10. ^{31}P NMR (left panel) and ^{77}Se NMR (right panel) spectra of compound **14b**.

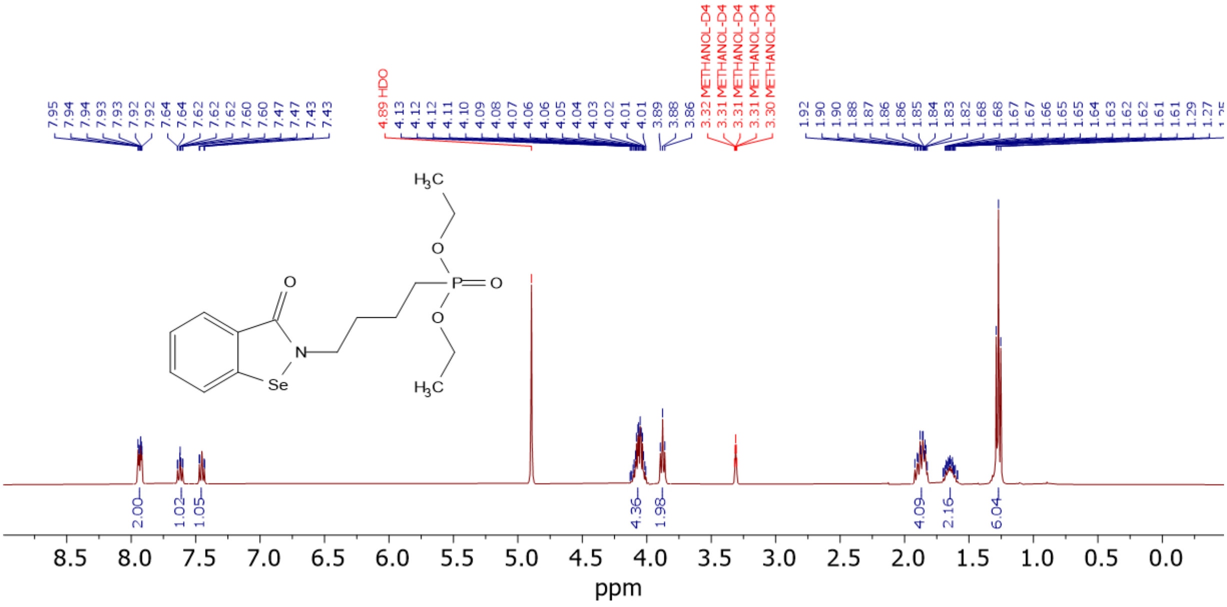

Figure S11. ^{1}H NMR spectrum of compound **14c**.

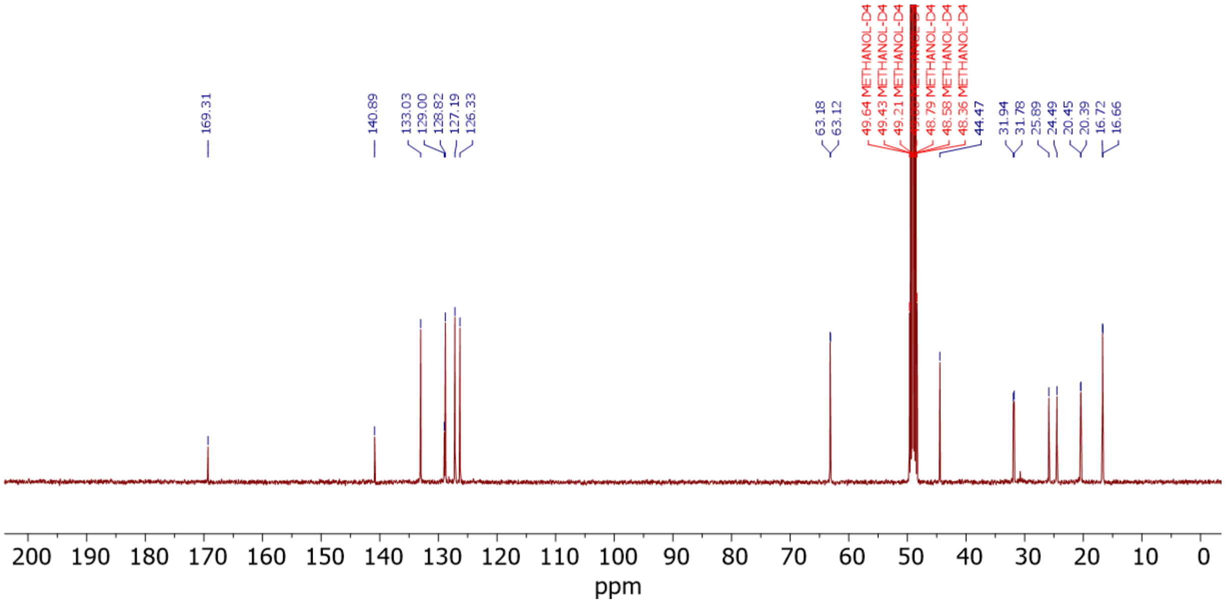

Figure S12. ^{13}C NMR spectrum of compound **14c**.

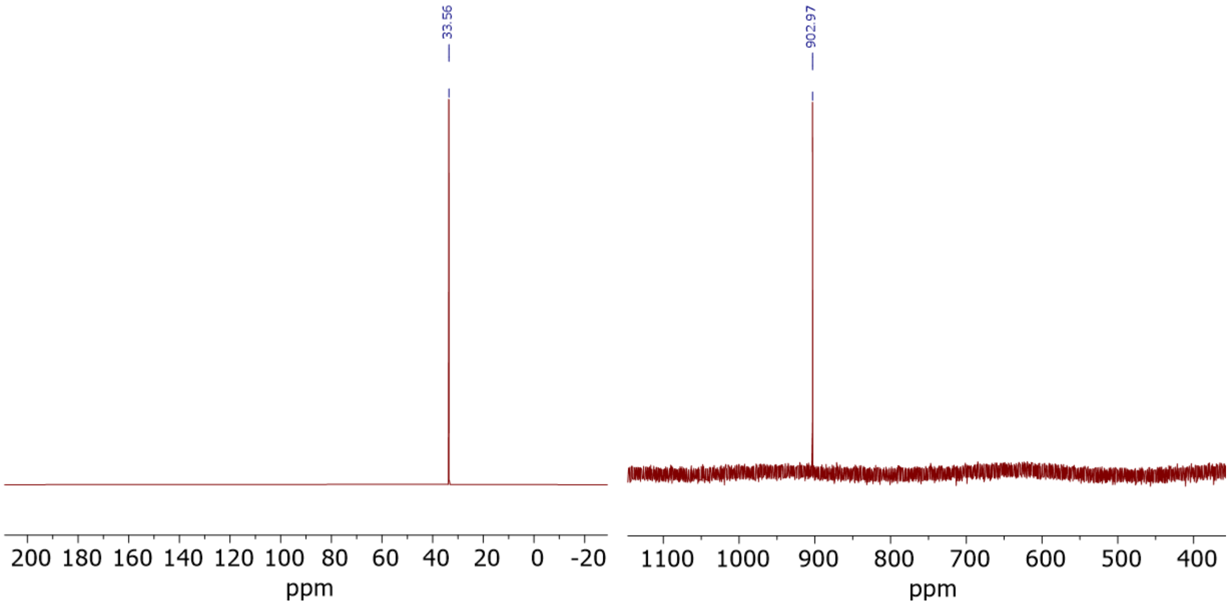

Figure S13. ^{31}P NMR (left panel) and ^{77}Se NMR (right panel) spectra of compound **14c**.

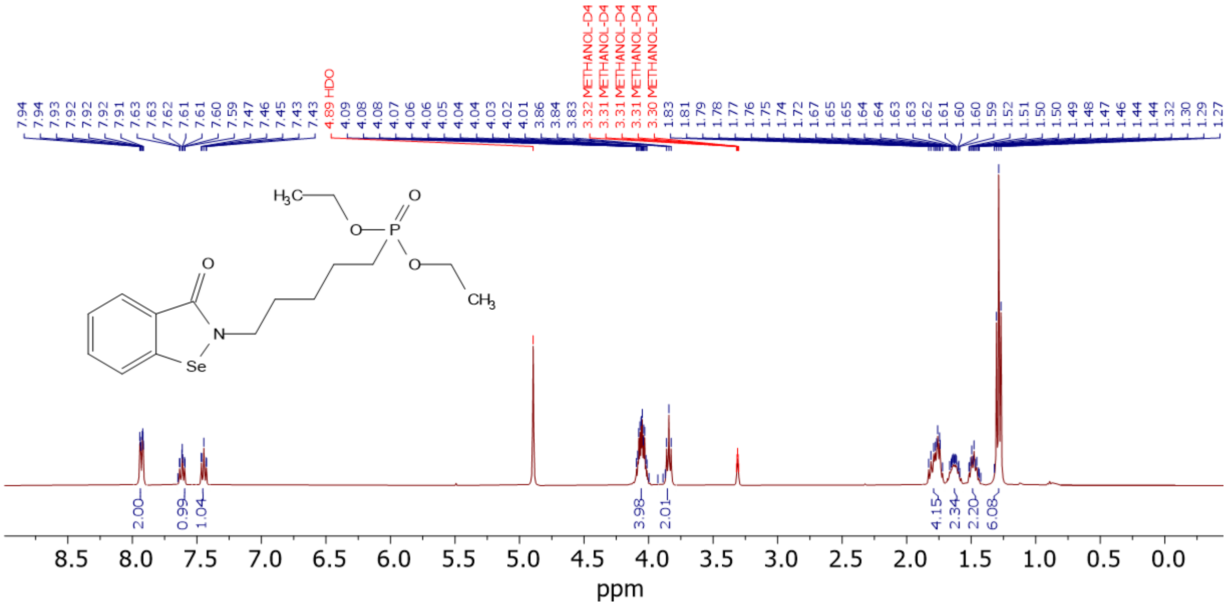

Figure S14. ^{1}H NMR spectrum of compound **14d**.

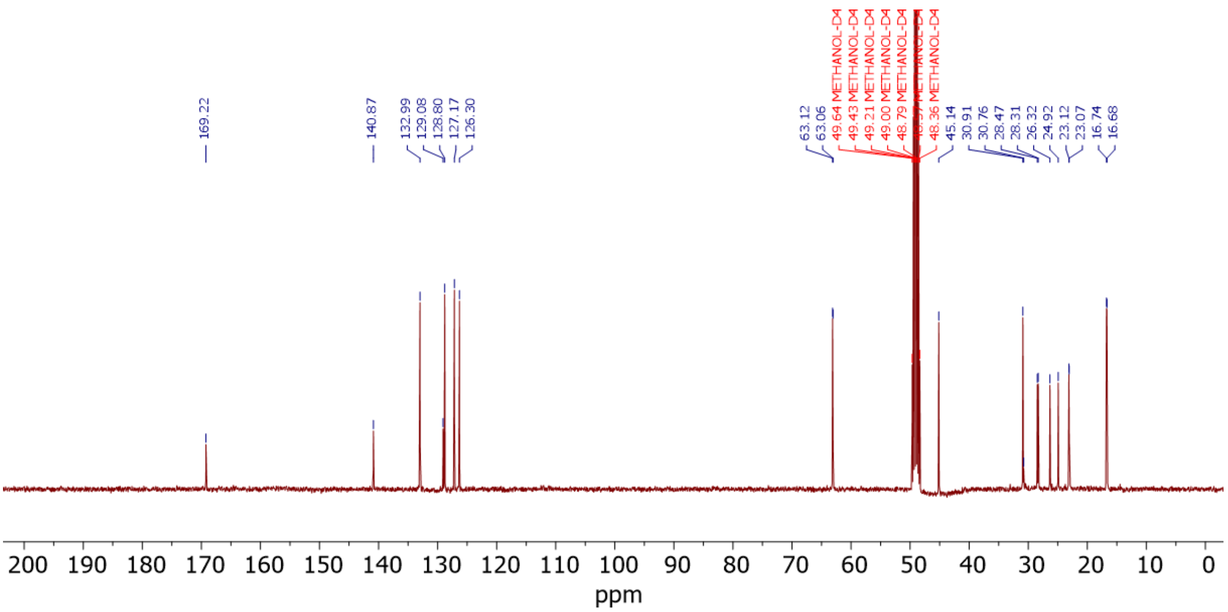

Figure S15. ^{13}C NMR spectrum of compound **14d**.

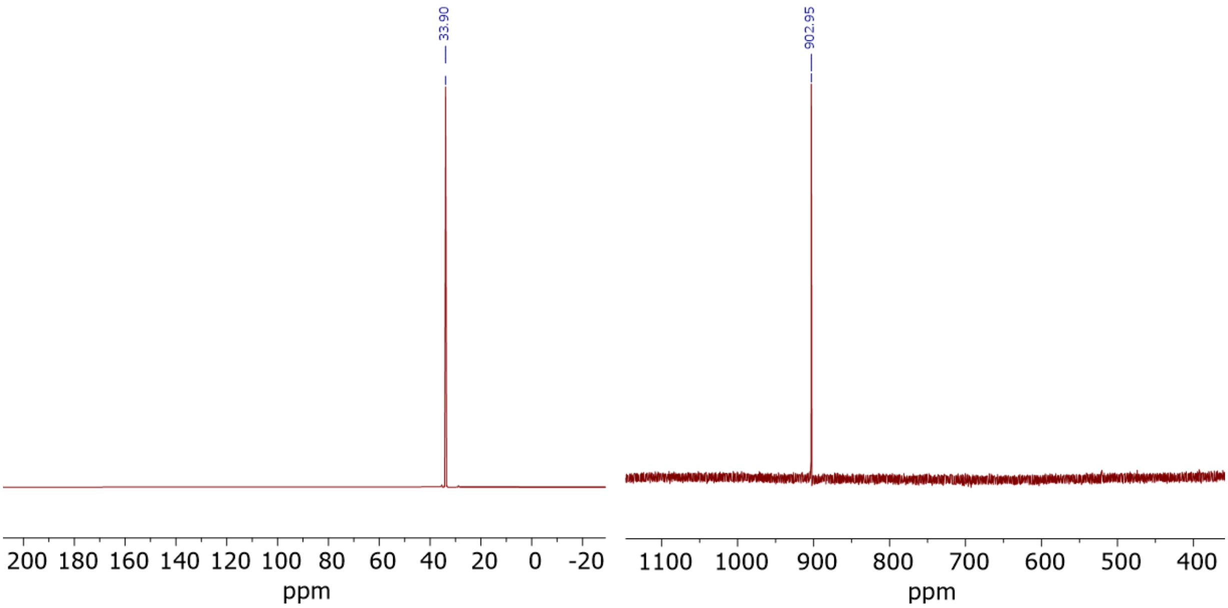

Figure S16. ^{31}P NMR (left panel) and ^{77}Se NMR (right panel) spectra of compound **14d**.

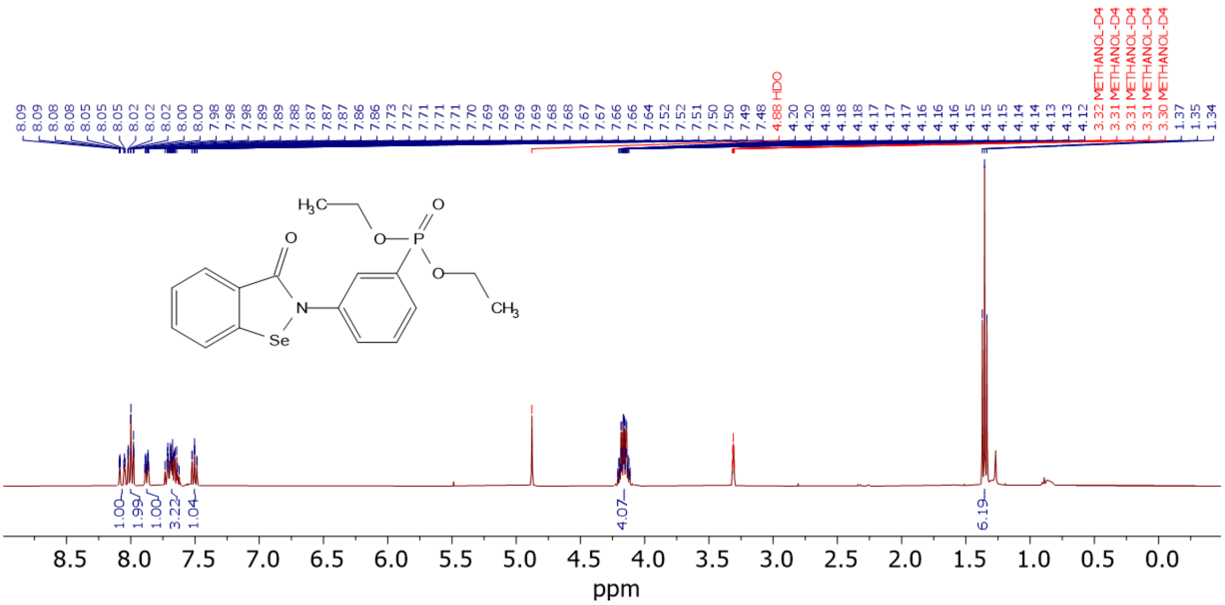

Figure S17. ^{1}H NMR spectrum of compound **14e**.

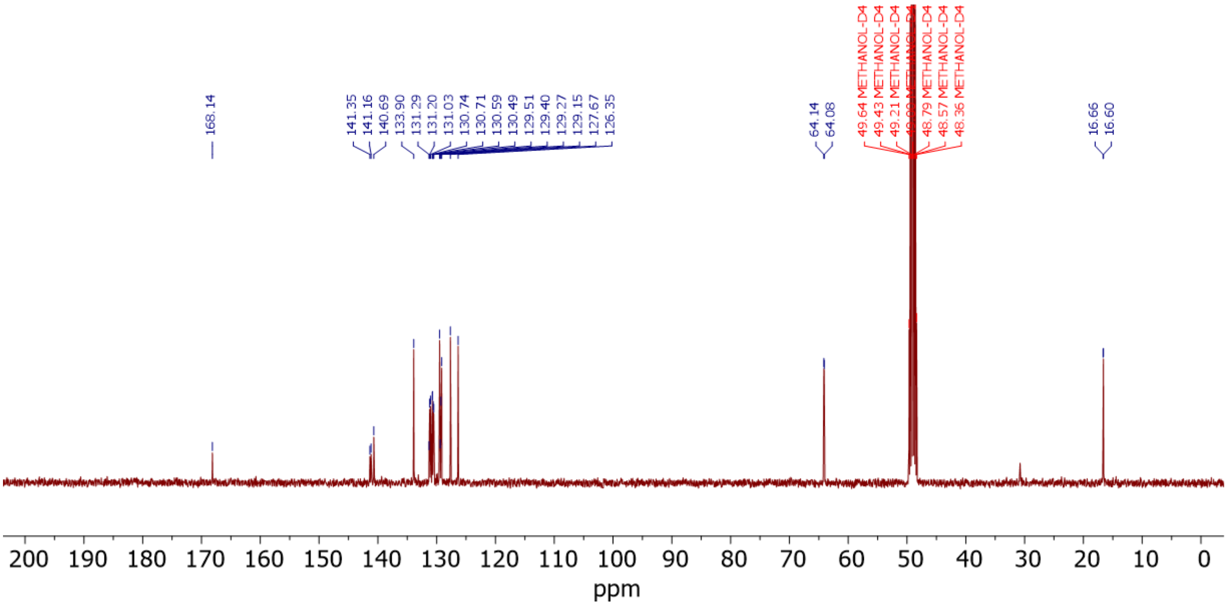

Figure S18. ^{13}C NMR spectrum of compound **14e**.

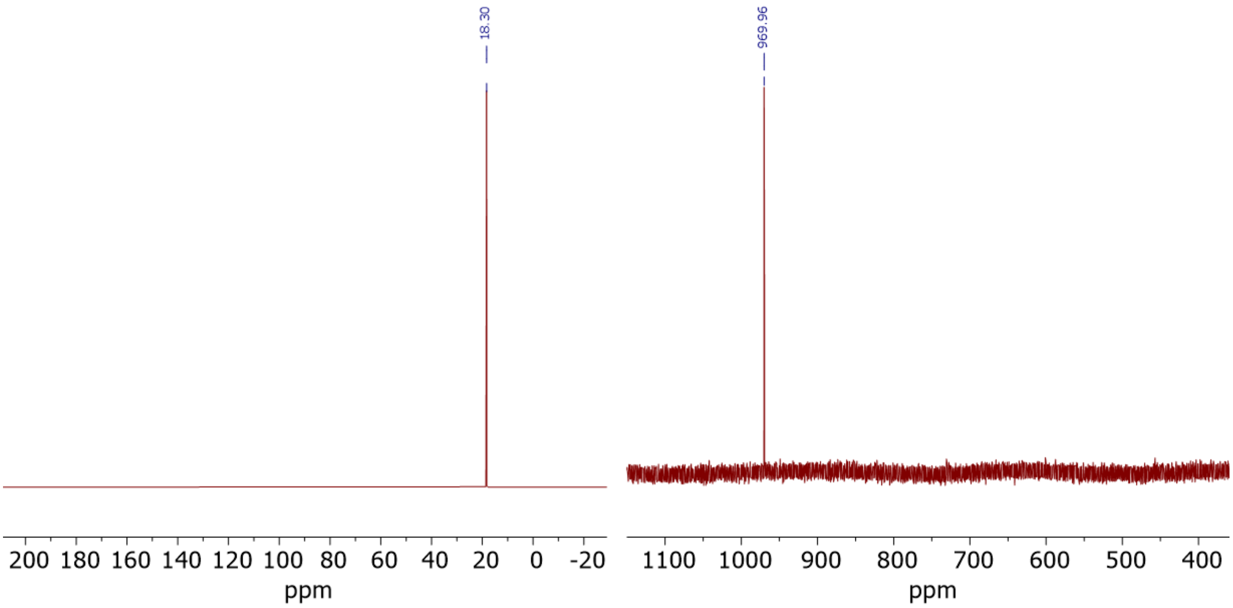

Figure S19. ^{31}P NMR (left panel) and ^{77}Se NMR (right panel) spectra of compound **14e**.

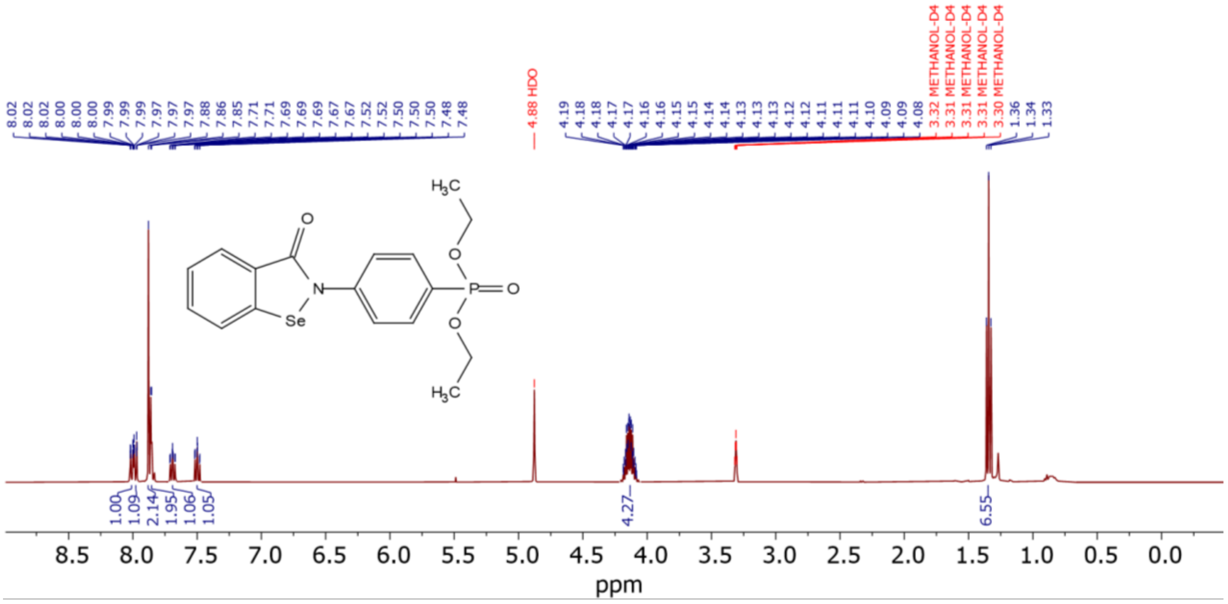

Figure S20. ^{1}H NMR spectrum of compound **14f**.

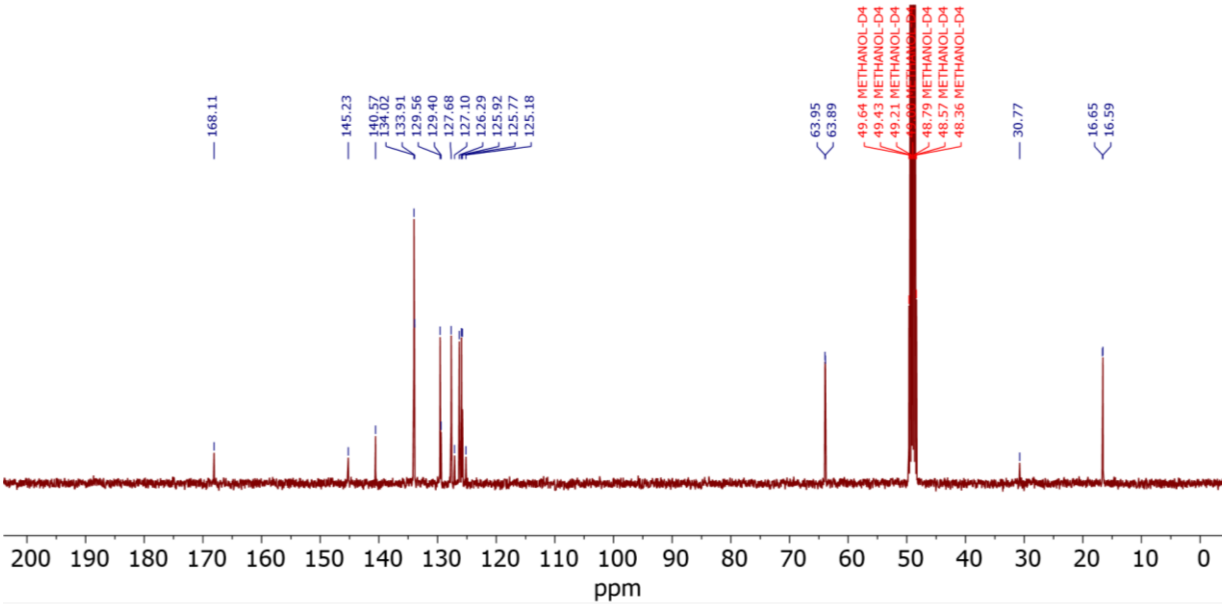

Figure S21. ^{13}C NMR spectrum of compound **14f**.

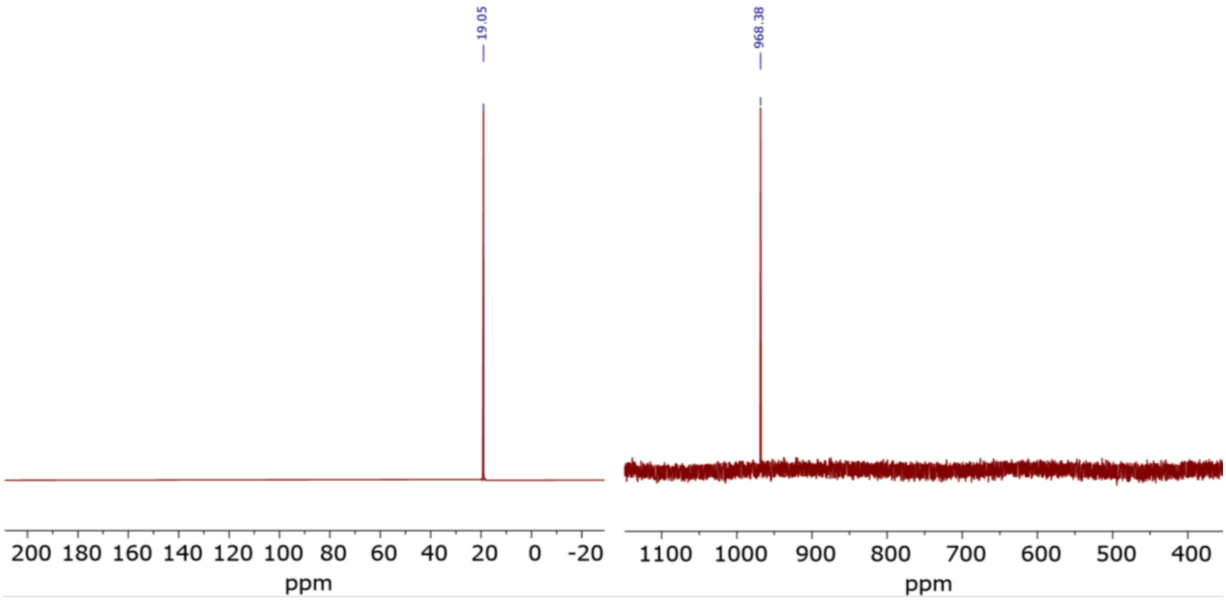

Figure S22. ^{31}P NMR (left panel) and ^{77}Se NMR (right panel) spectra of compound **14f**.

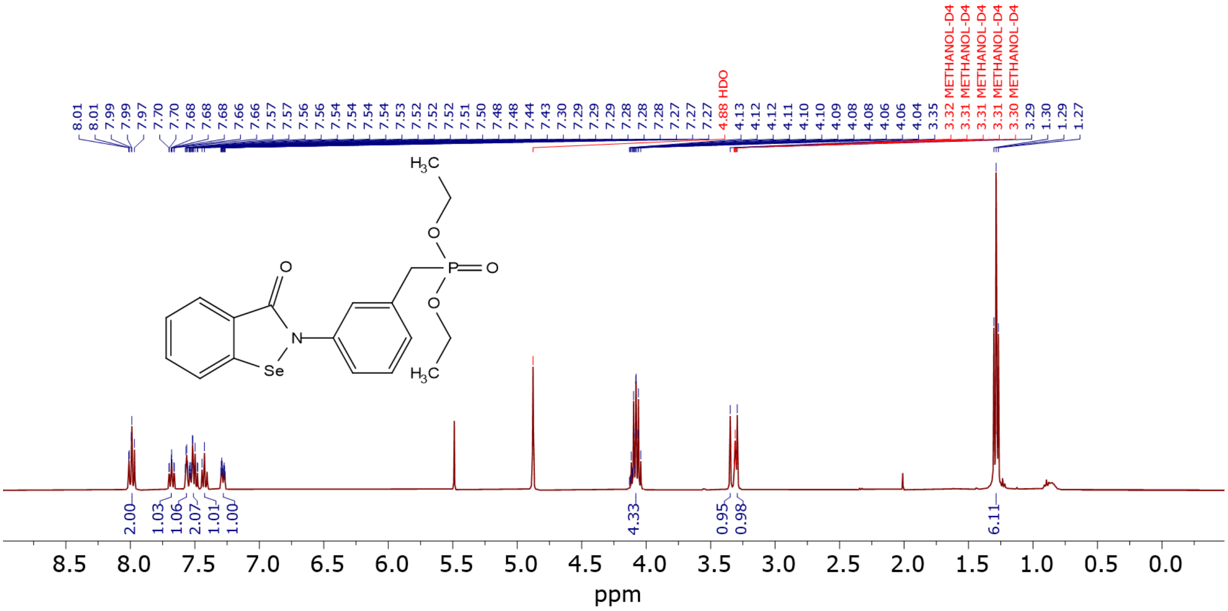

Figure S23. ^{1}H NMR spectrum of compound **14g**.

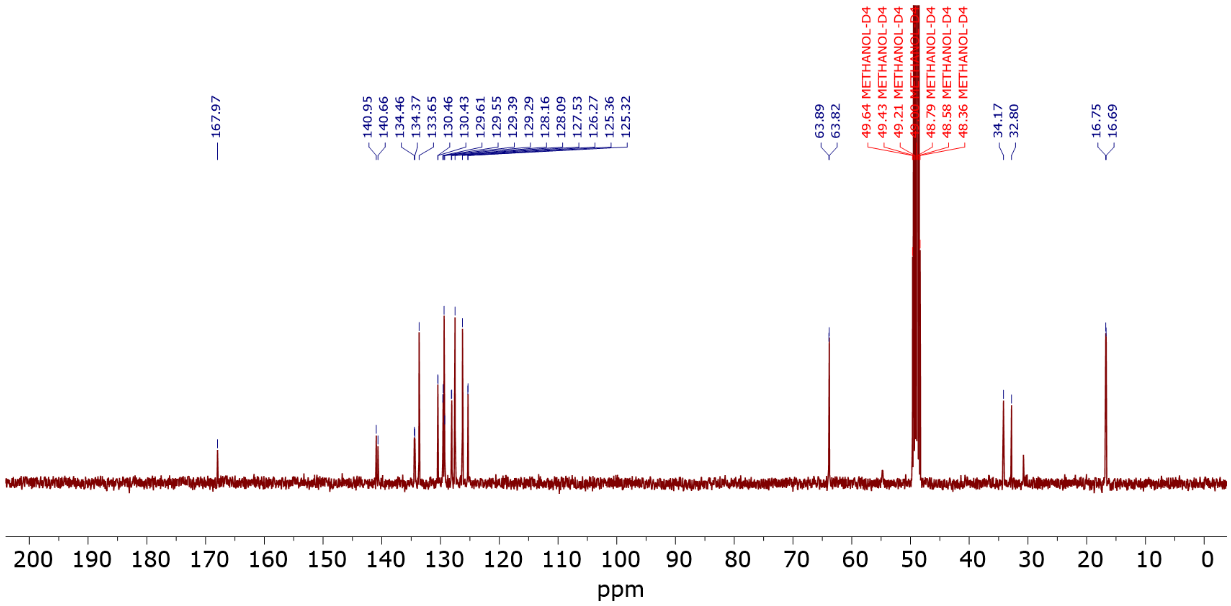

Figure S24. ^{13}C NMR spectrum of compound **14g**.

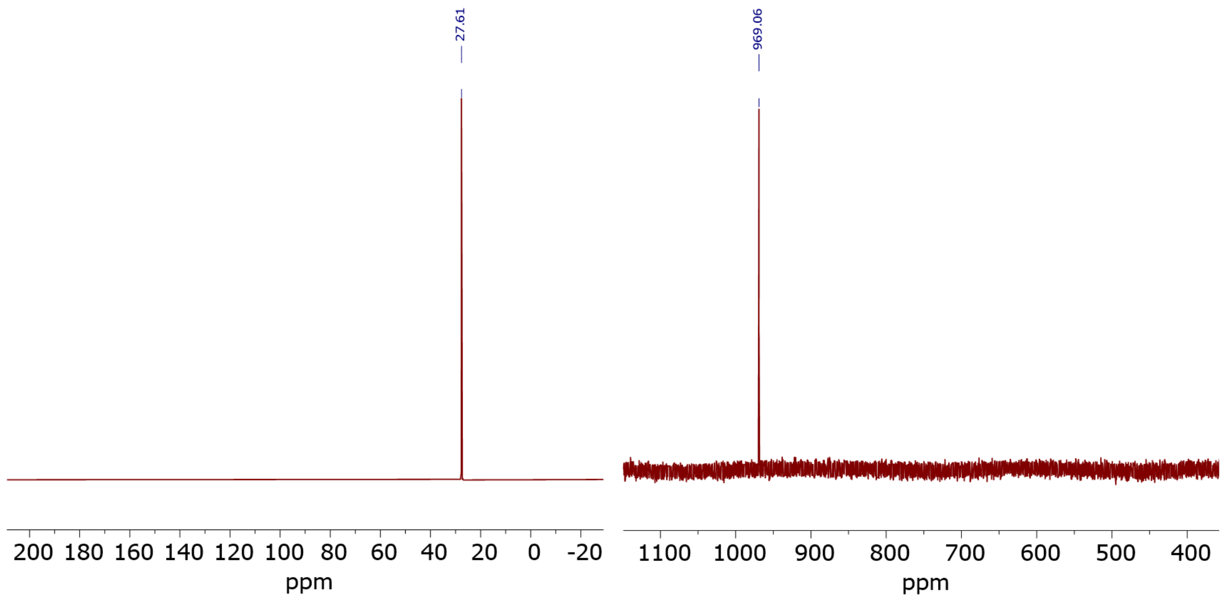

Figure S25. ^{31}P NMR (left panel) and ^{77}Se NMR (right panel) spectra of compound **14g**.

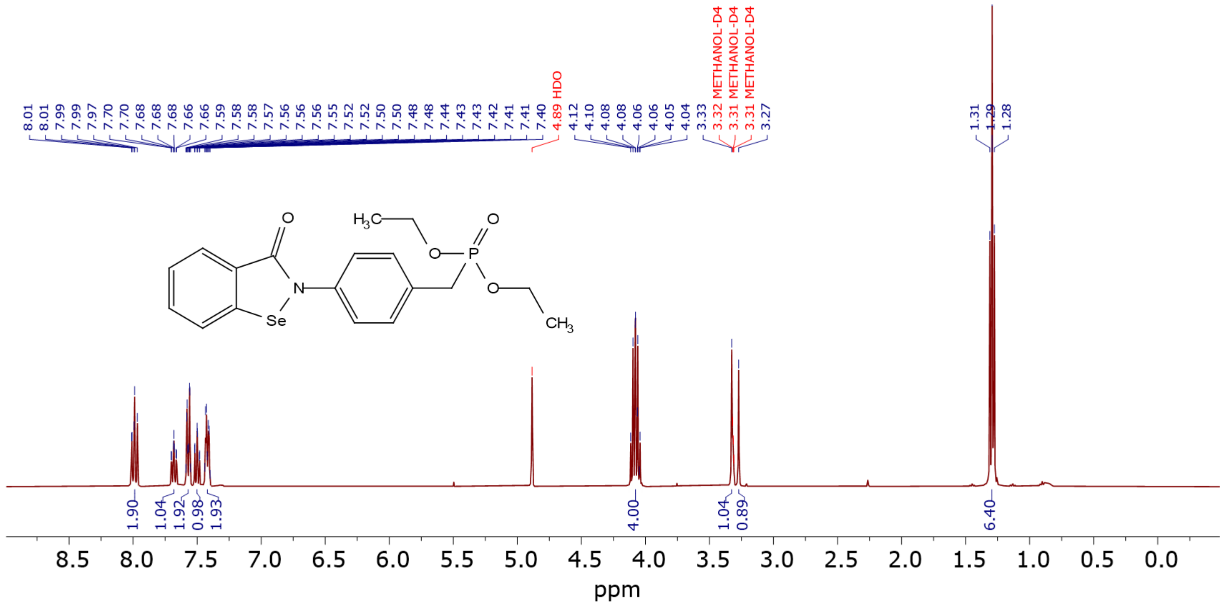

Figure S26. ^{1}H NMR spectrum of compound **14h**.

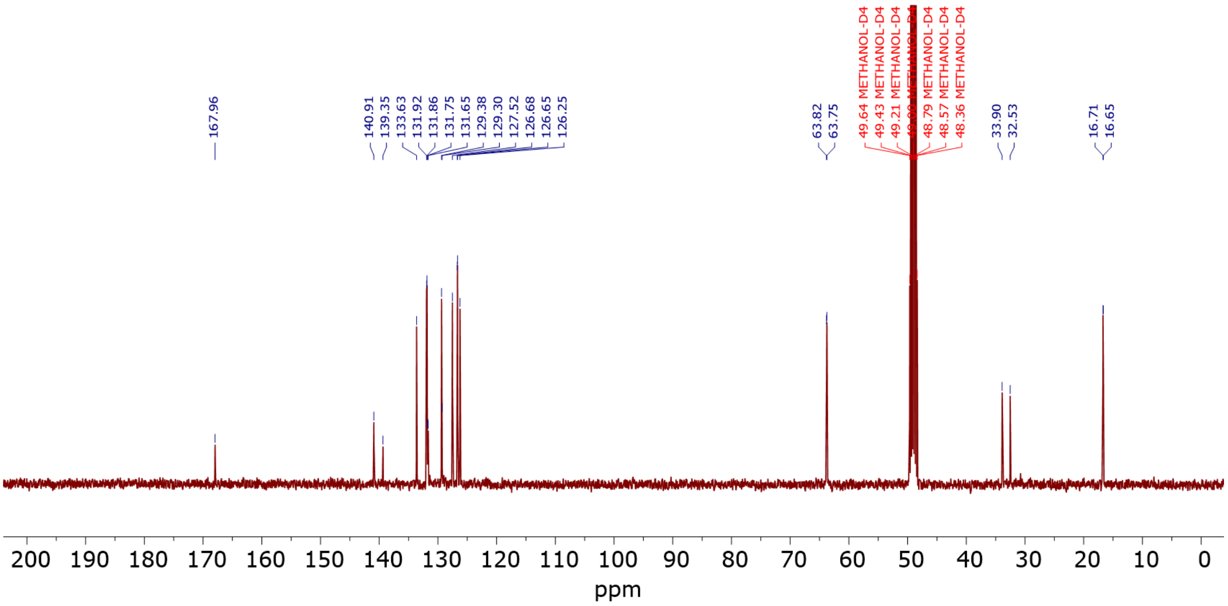

Figure S27. ^{13}C NMR spectrum of compound **14h**.

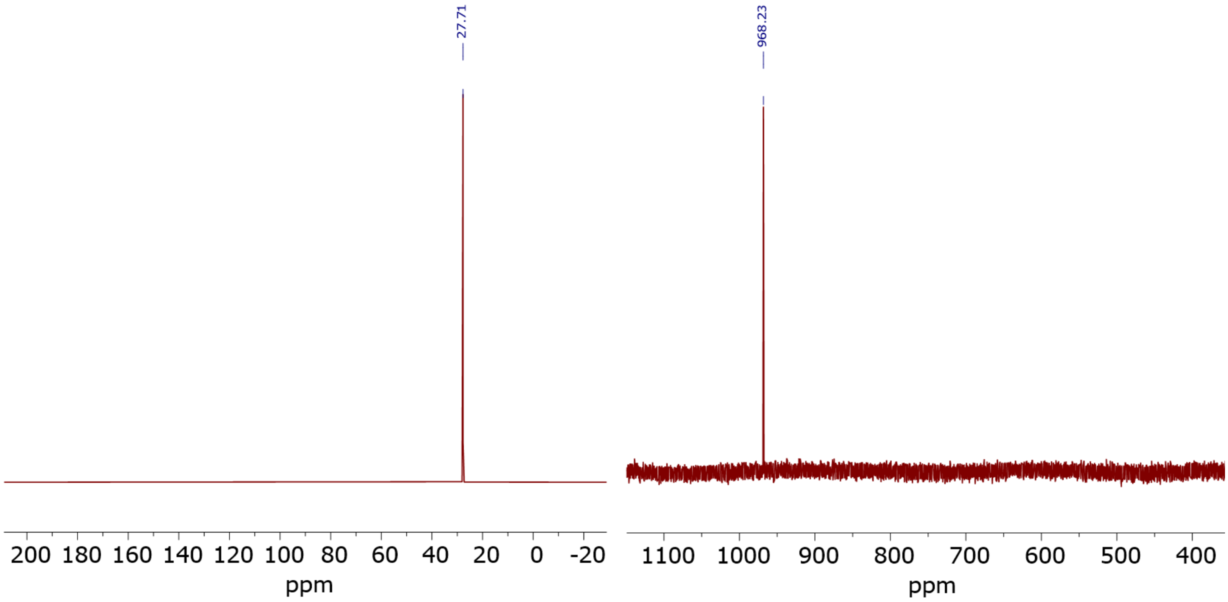

Figure S28. ^{31}P NMR (left panel) and ^{77}Se NMR (right panel) spectra of compound **14h**.

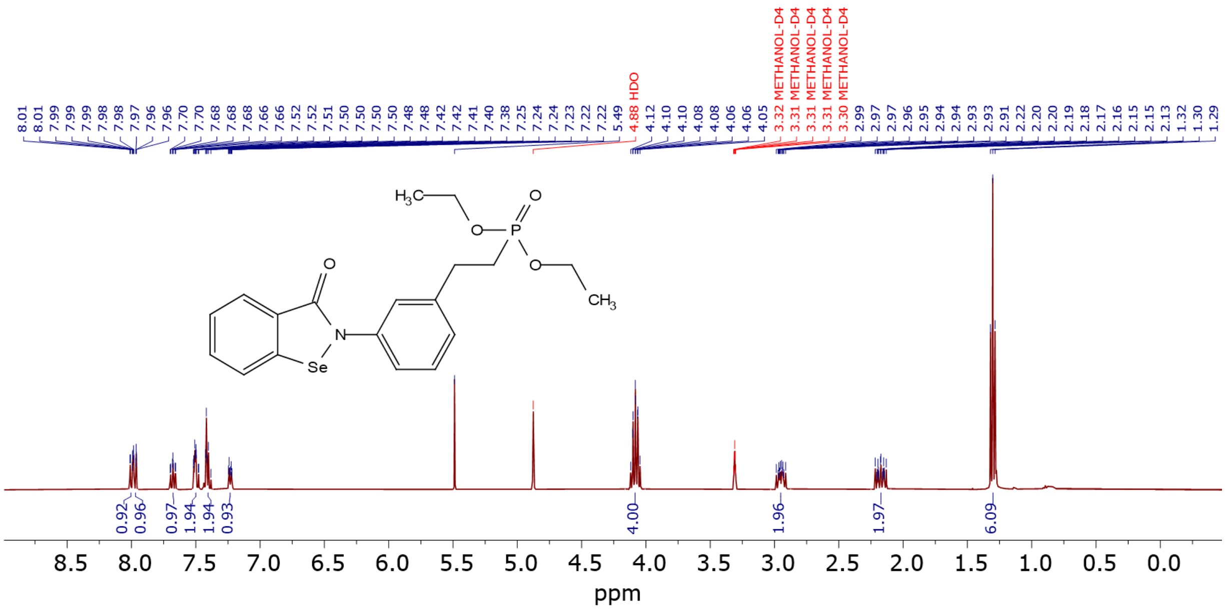

Figure S29. ^{1}H NMR spectrum of compound **14i**.

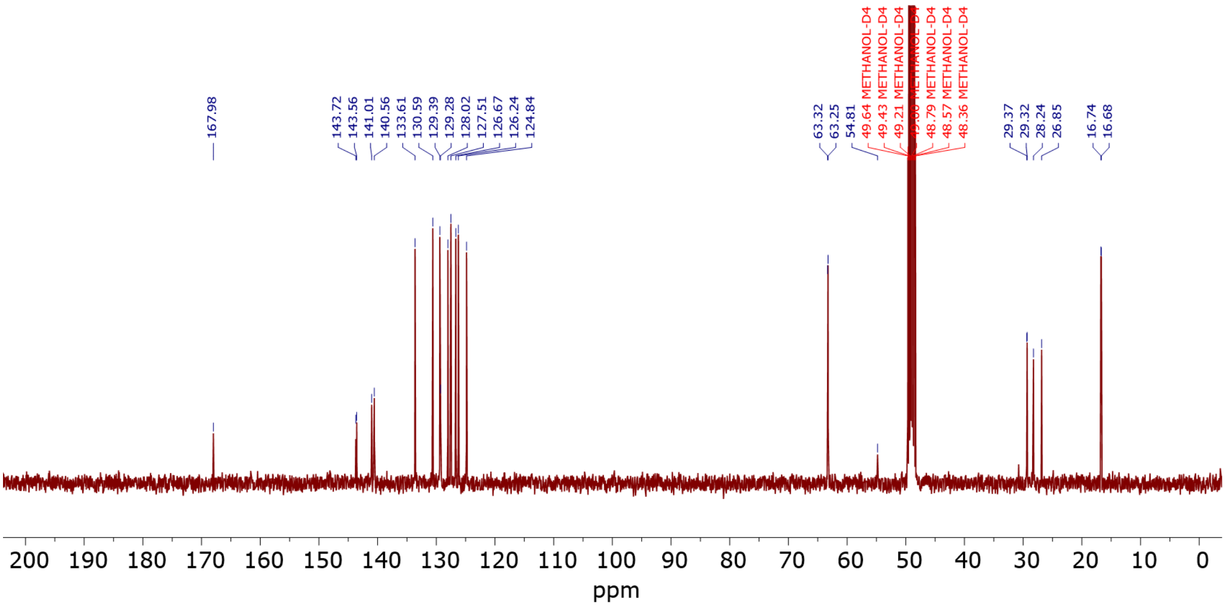

Figure S30. ^{13}C NMR spectrum of compound **14i**.

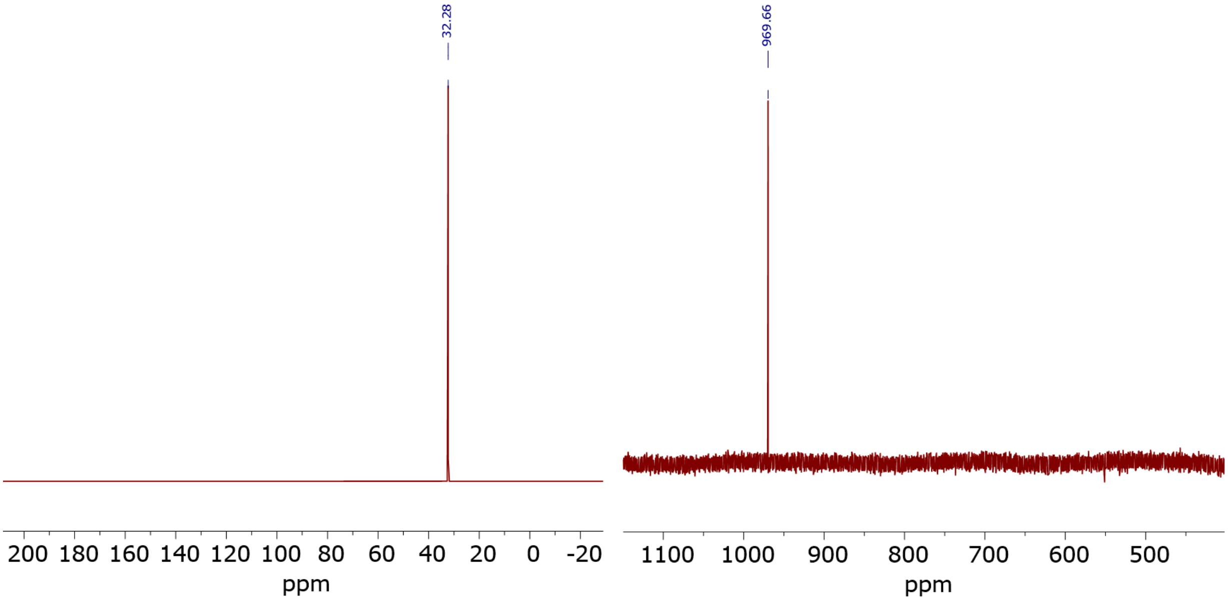

Figure S31. ^{31}P NMR (left panel) and ^{77}Se NMR (right panel) spectra of compound **14i**.

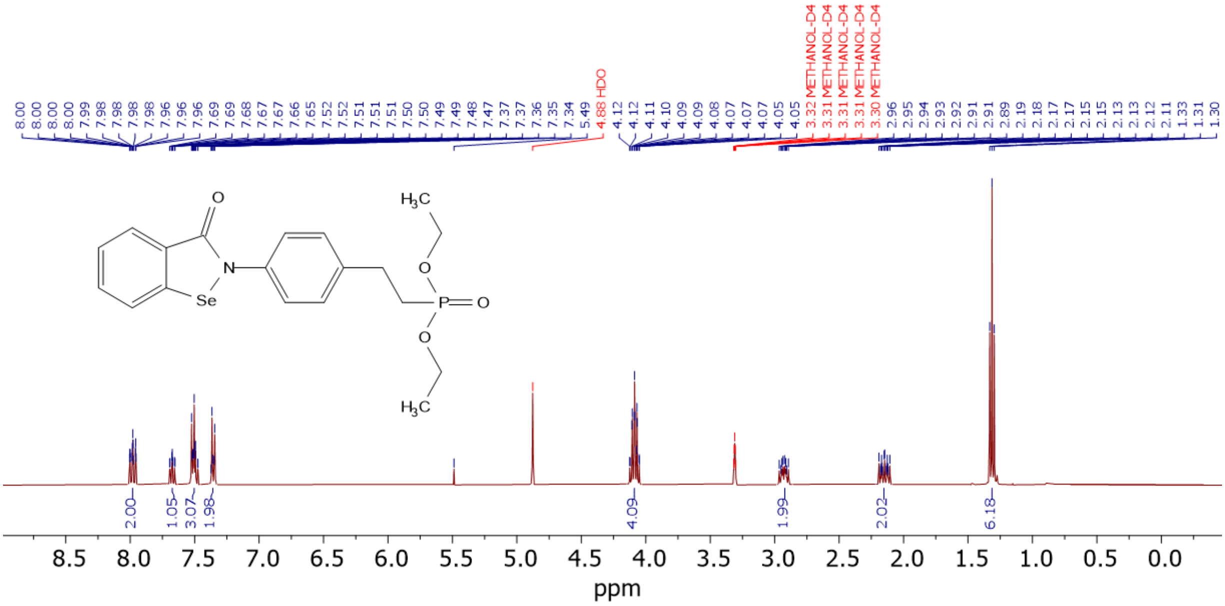

Figure S32. ^{1}H NMR spectrum of compound **14j**.

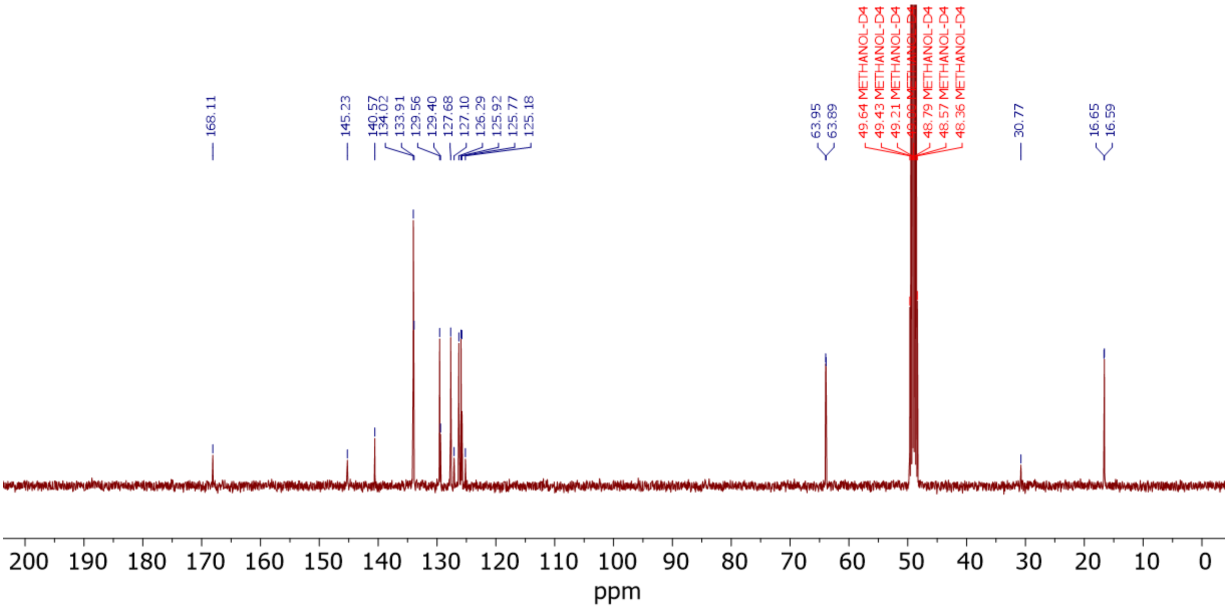

Figure S33. ^{13}C NMR spectrum of compound **14j**.

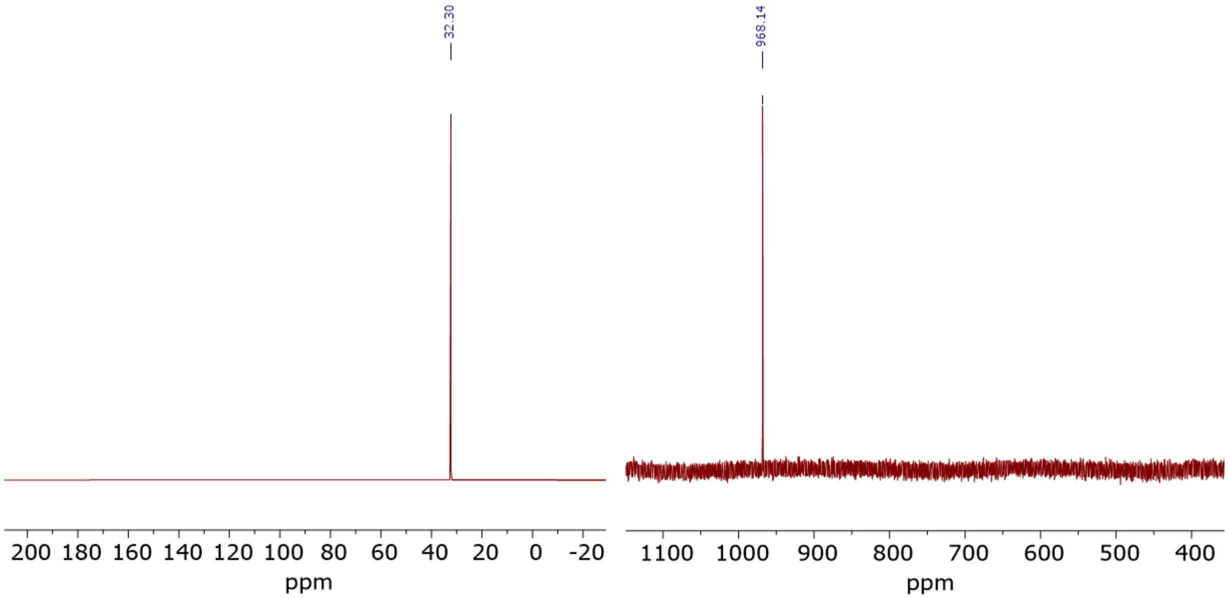

Figure S34. ^{31}P NMR (left panel) and ^{77}Se NMR (right panel) spectra of compound **14j**.

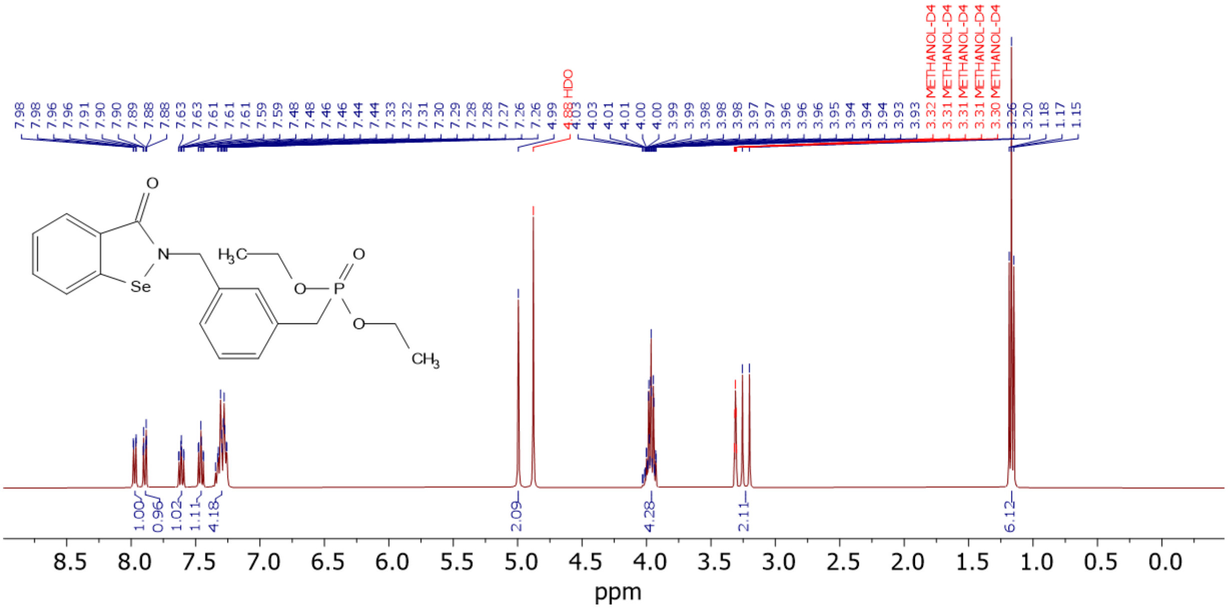

Figure S35. ^{1}H NMR spectrum of compound **14k**.

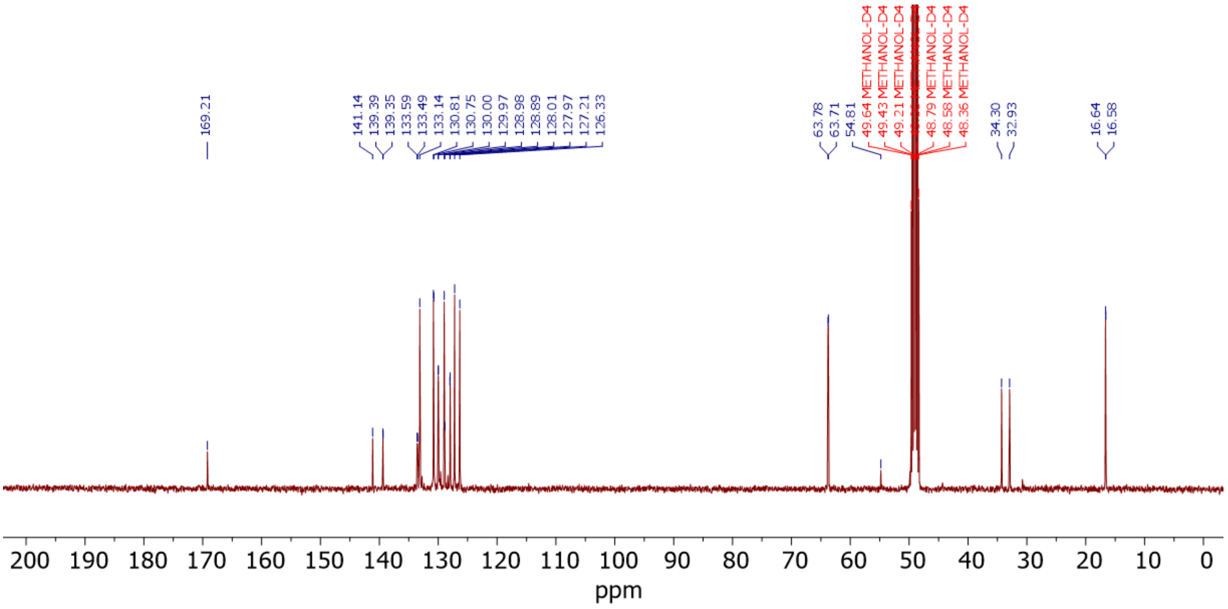

Figure S36. ^{13}C NMR spectrum of compound **14k**.

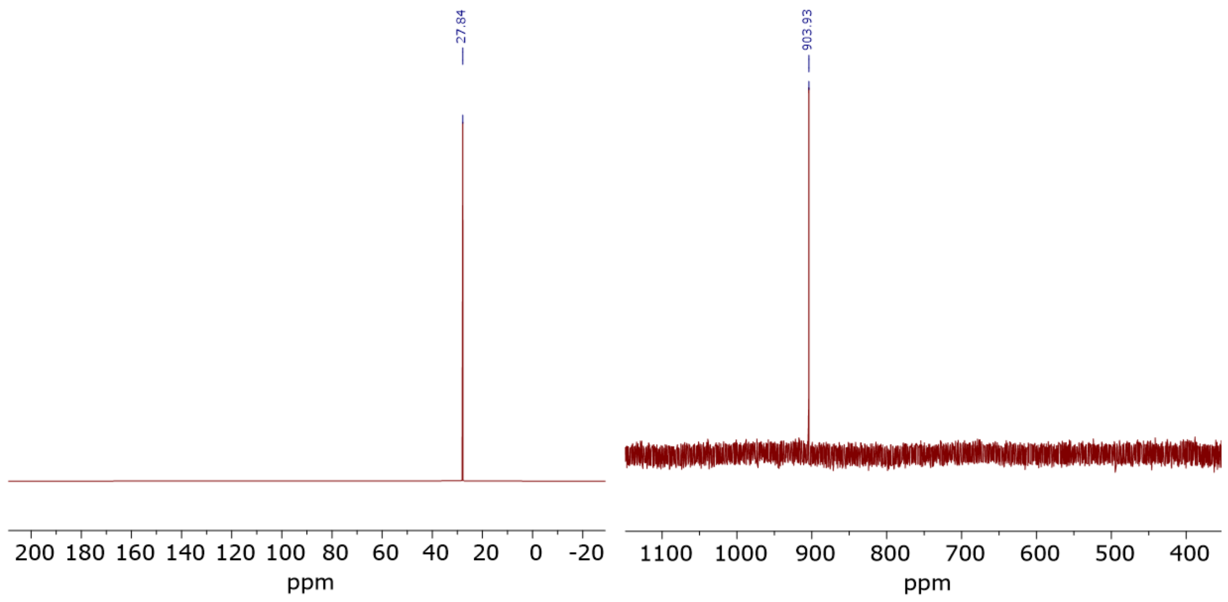

Figure S37. ^{31}P NMR (left panel) and ^{77}Se NMR (right panel) spectra of compound **14k**.

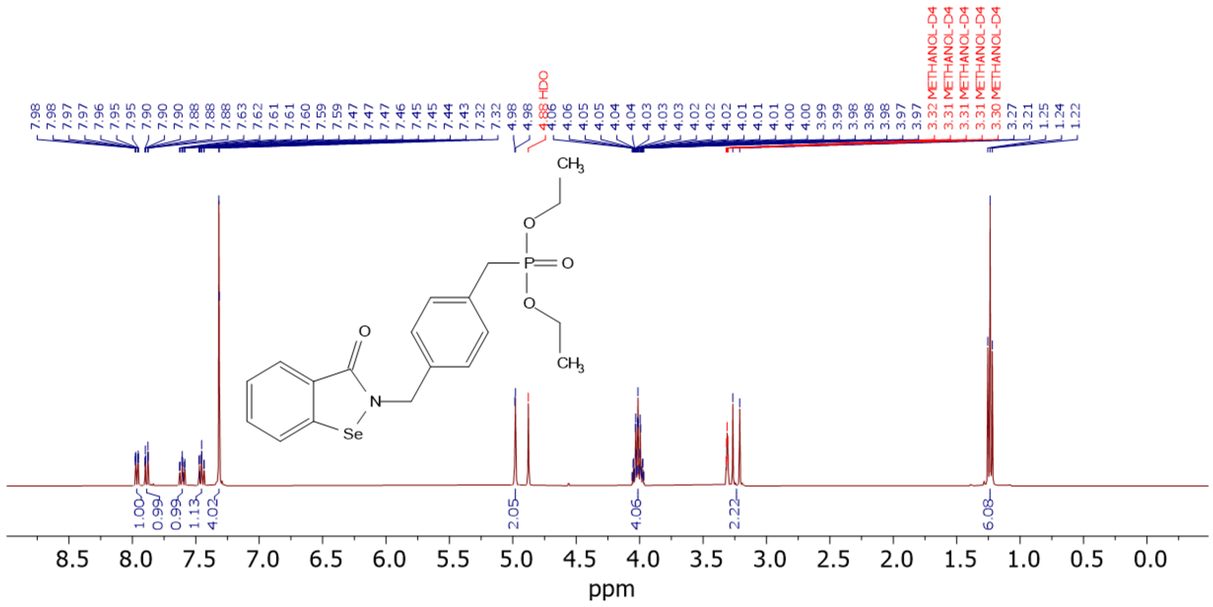

Figure S38. ^{1}H NMR spectrum of compound **14l**.

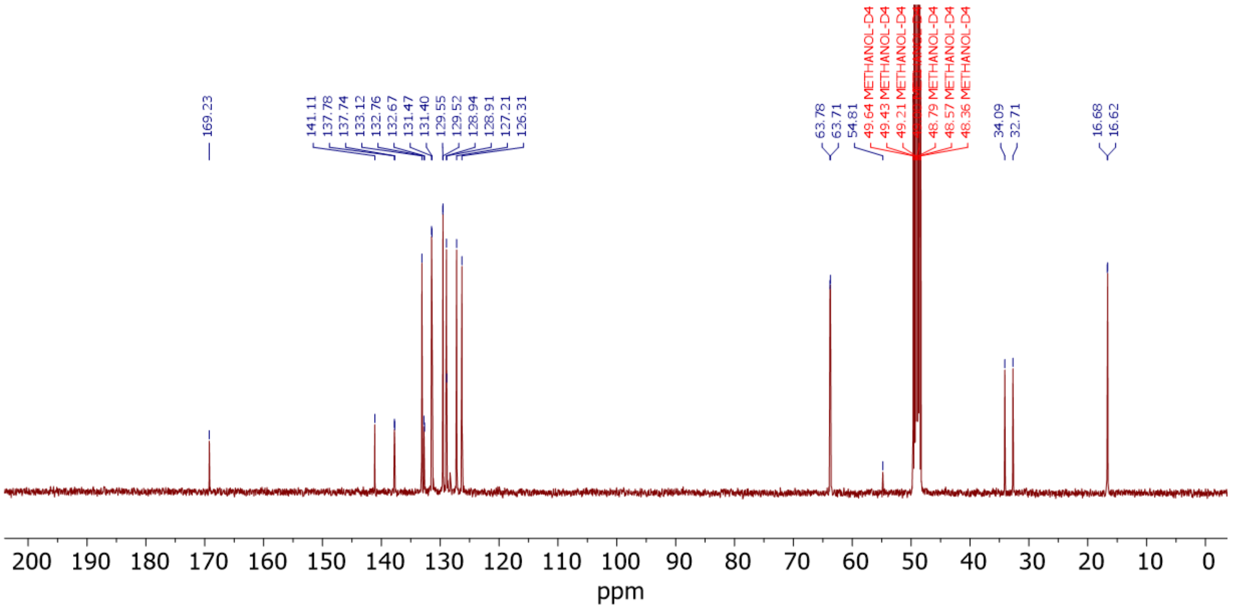

Figure S39. ^{13}C NMR spectrum of compound **14l**.

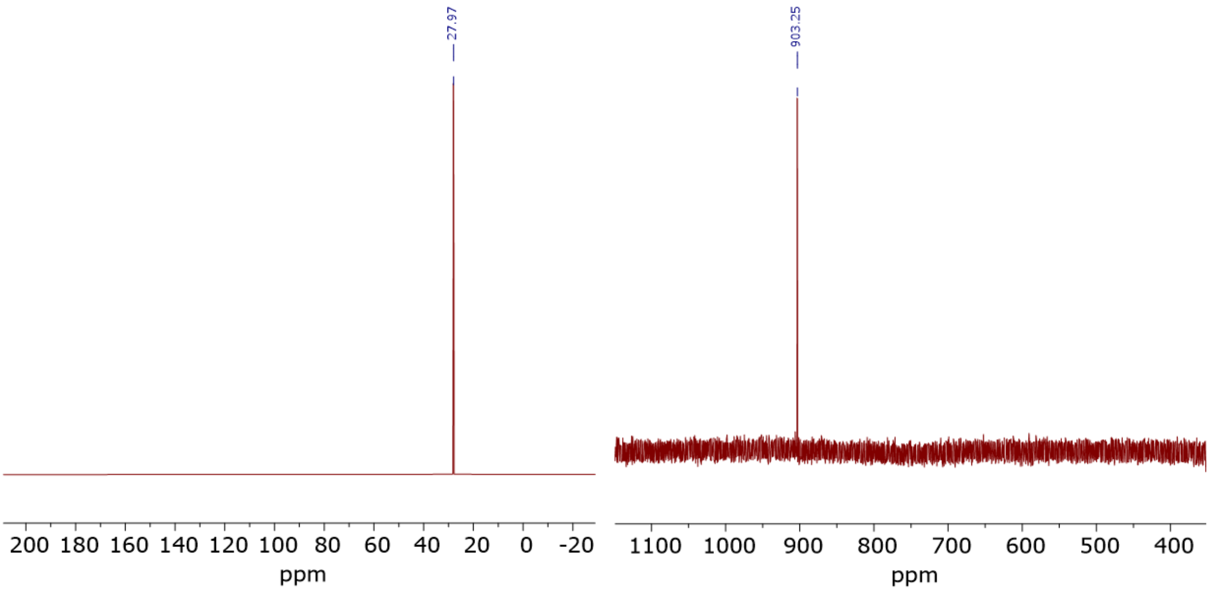

Figure S40. ^{31}P NMR (left panel) and ^{77}Se NMR (right panel) spectra of compound **14l**.

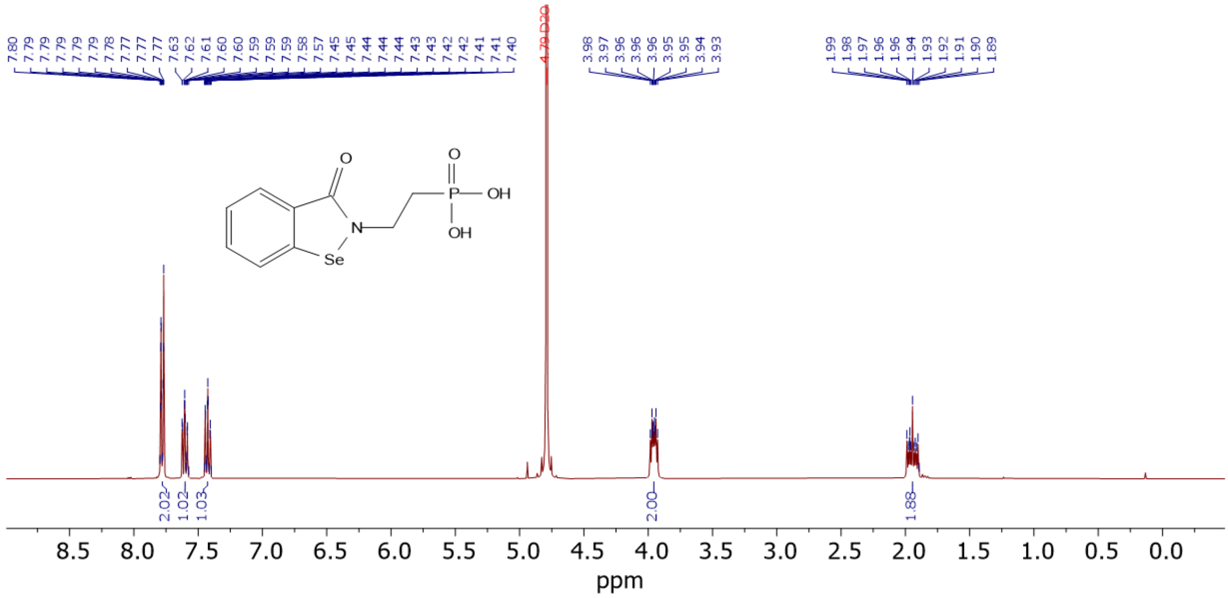

Figure S41. ^{1}H NMR spectrum of compound **1a**.

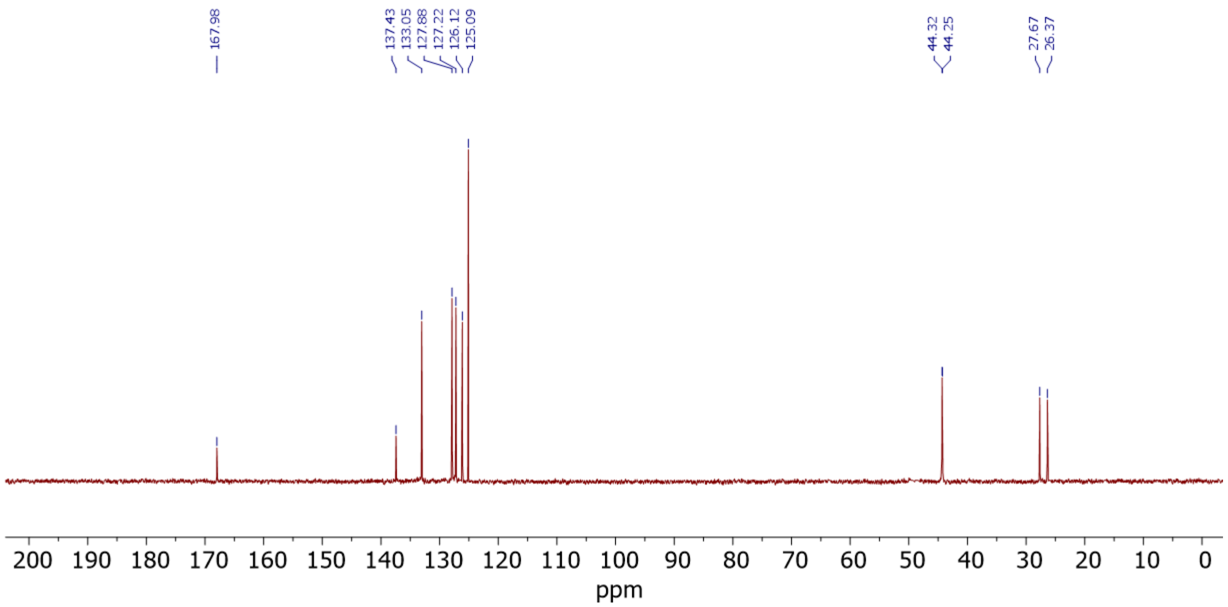

Figure S42. ^{13}C NMR spectrum of compound **1a**.

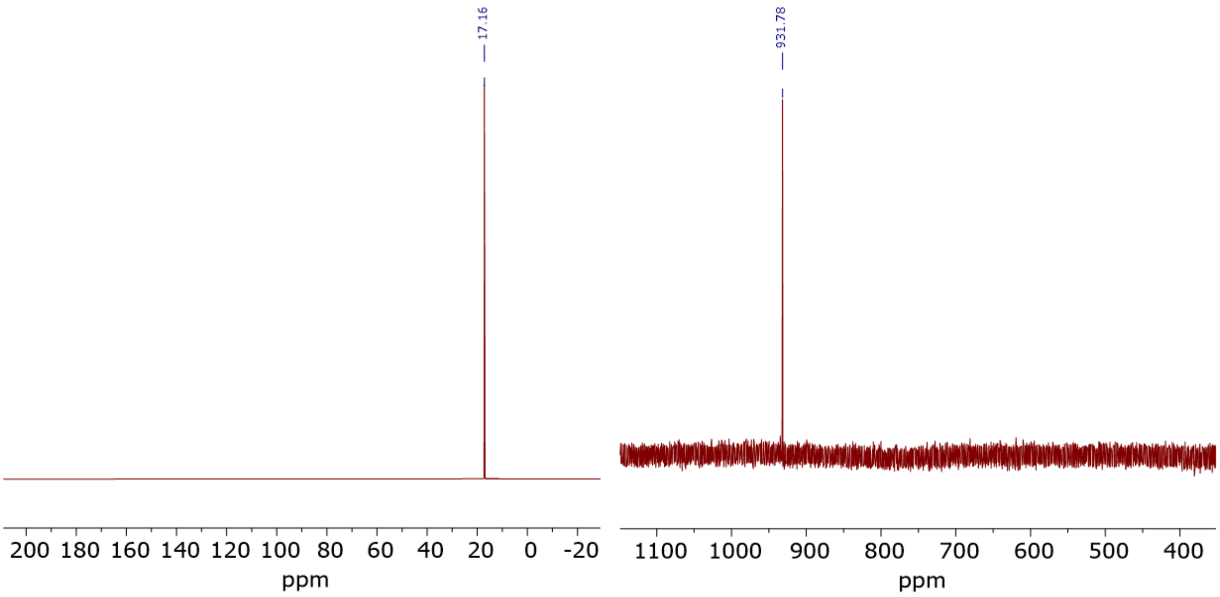

Figure S43. ^{31}P NMR (left panel) and ^{77}Se NMR (right panel) spectra of compound **1a**.

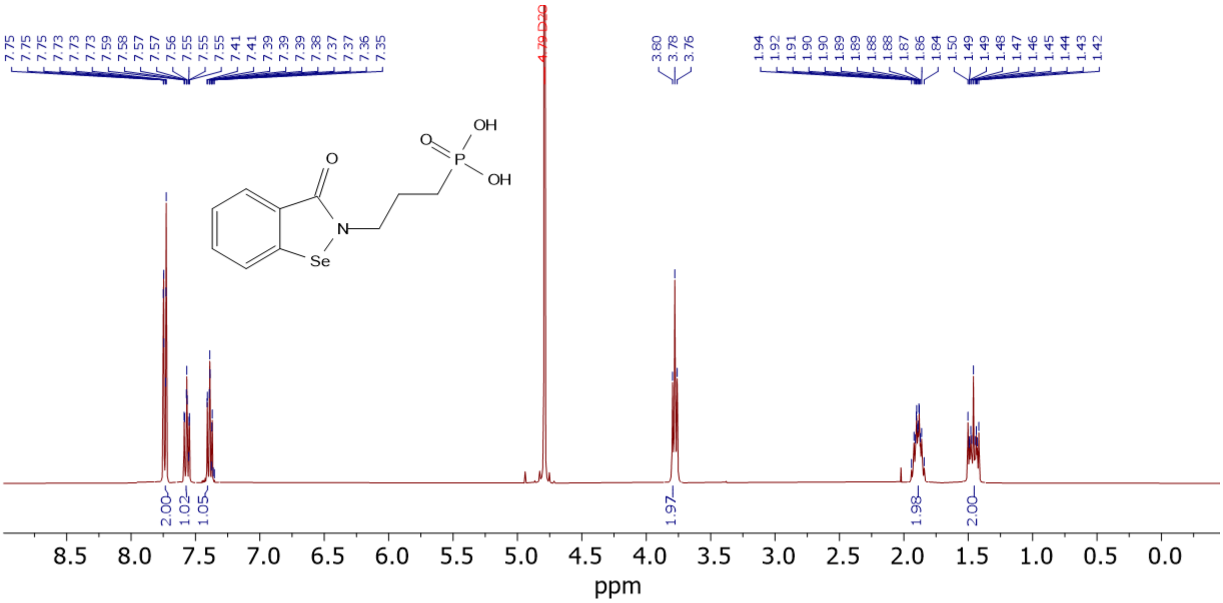

Figure S44. ^{1}H NMR spectrum of compound **1b**.

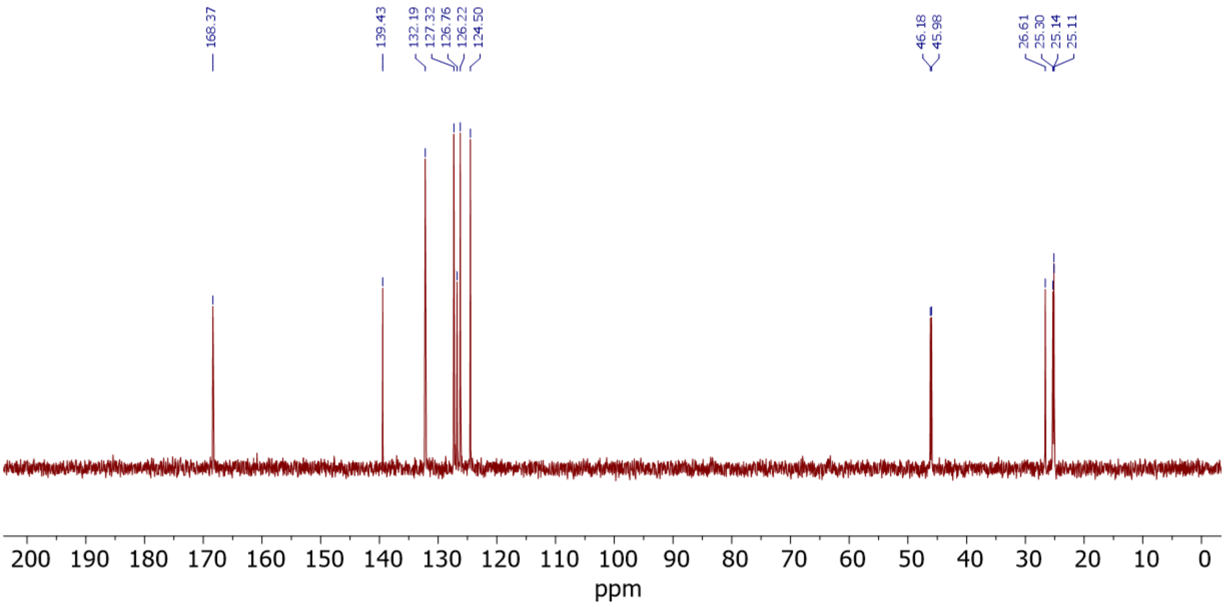

Figure S45. ^{13}C NMR spectrum of compound **1b**.

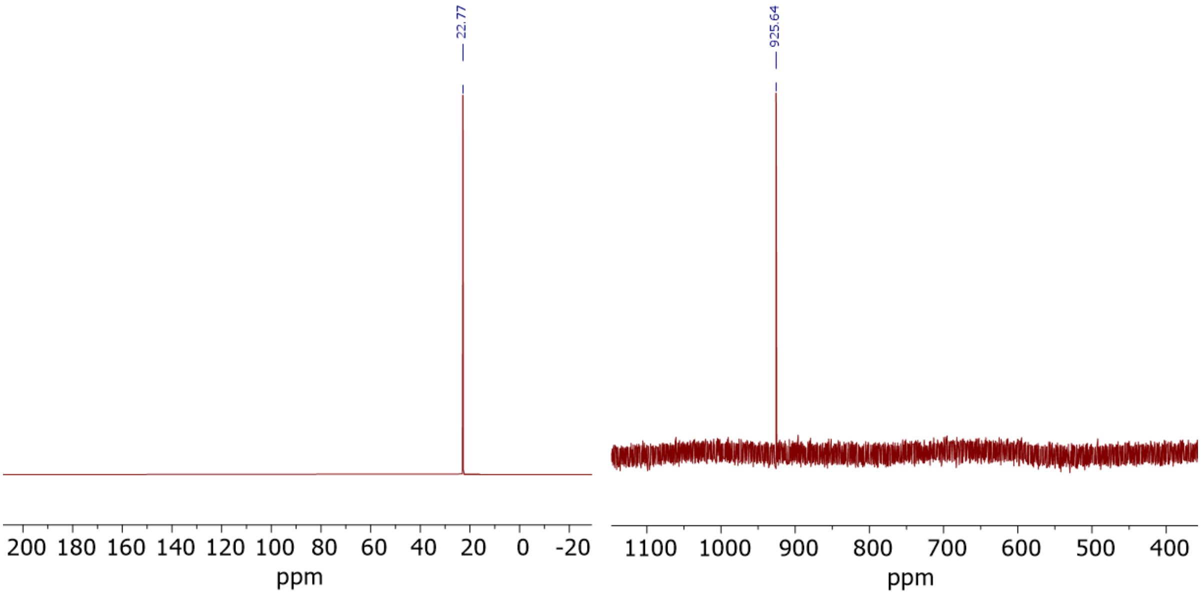

Figure S46. ^{31}P NMR (left panel) and ^{77}Se NMR (right panel) spectra of compound **1b**.

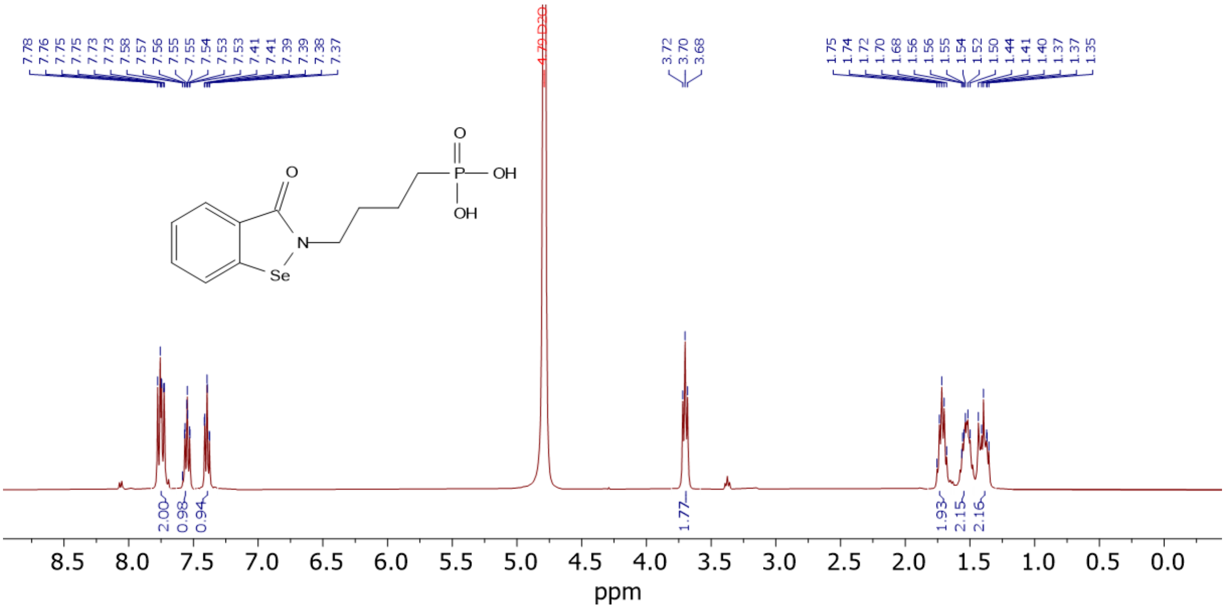

Figure S47. ^{1}H NMR spectrum of compound **1c**.

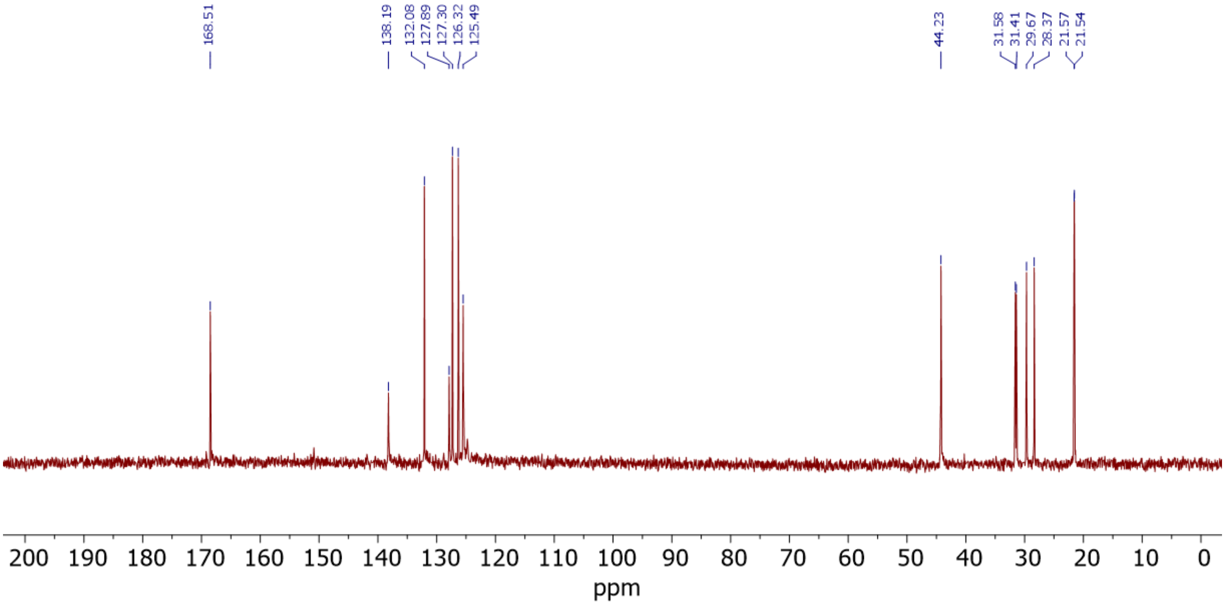

Figure S48. ^{13}C NMR spectrum of compound **1c**.

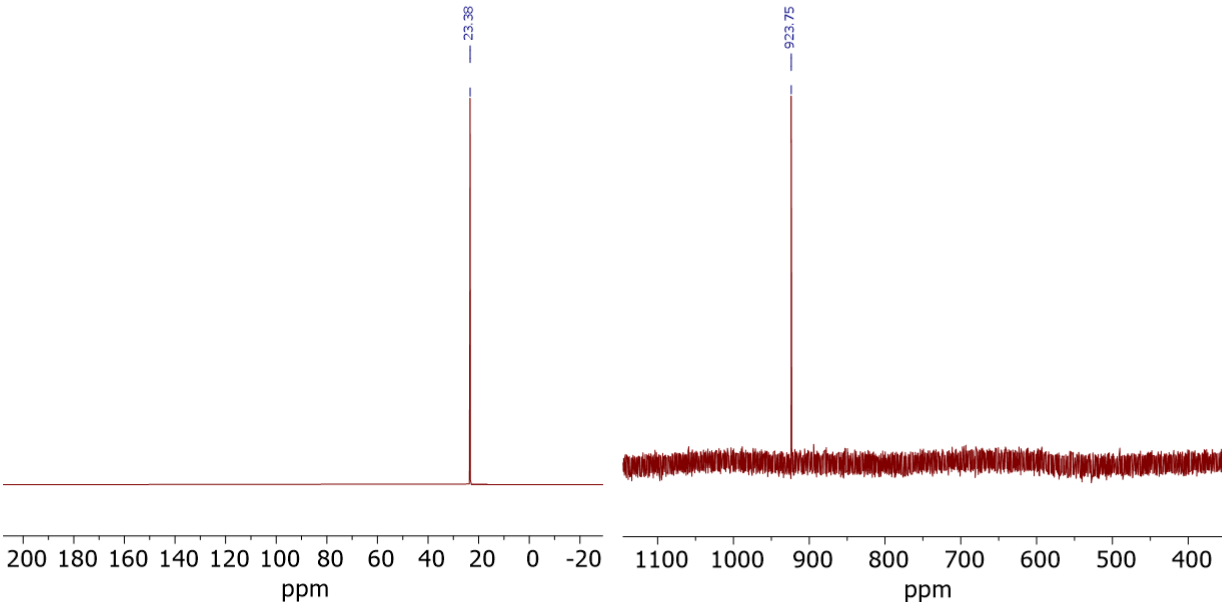

Figure S49. ^{31}P NMR (left panel) and ^{77}Se NMR (right panel) spectra of compound **1c**.

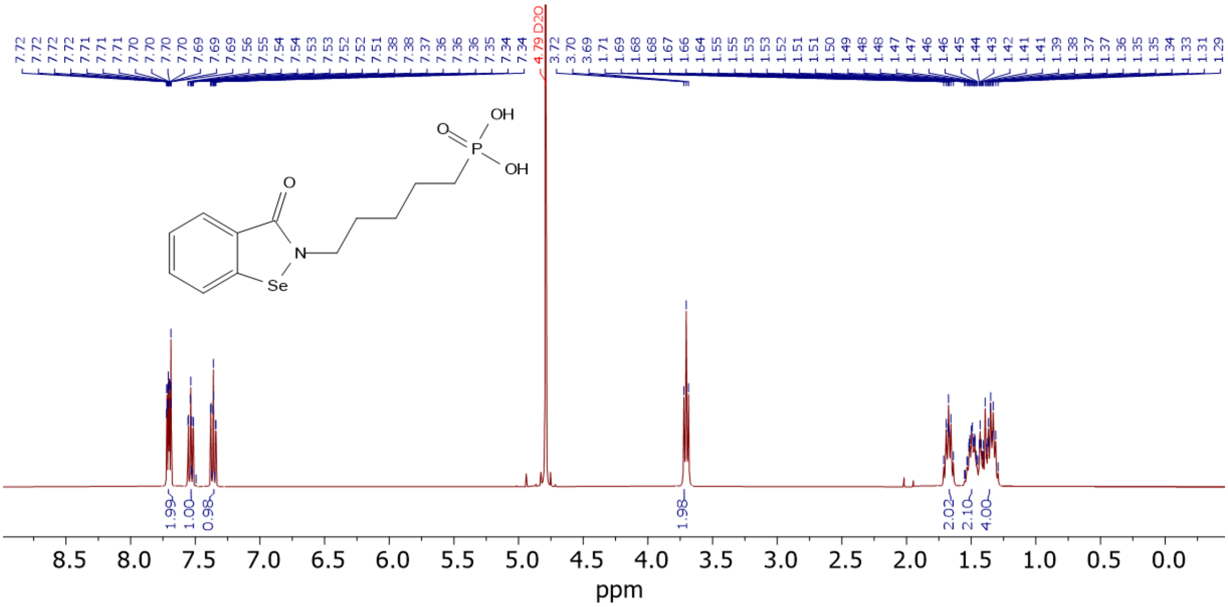

Figure S50. ^{1}H NMR spectrum of compound **1d**.

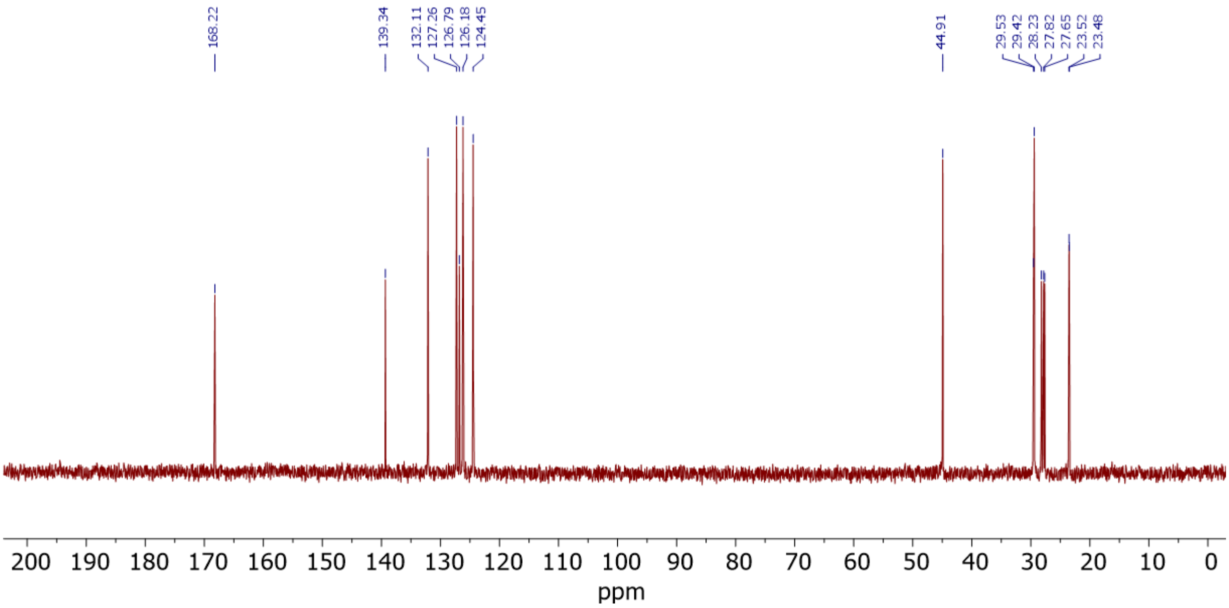

Figure S51. ^{13}C NMR spectrum of compound **1d**.

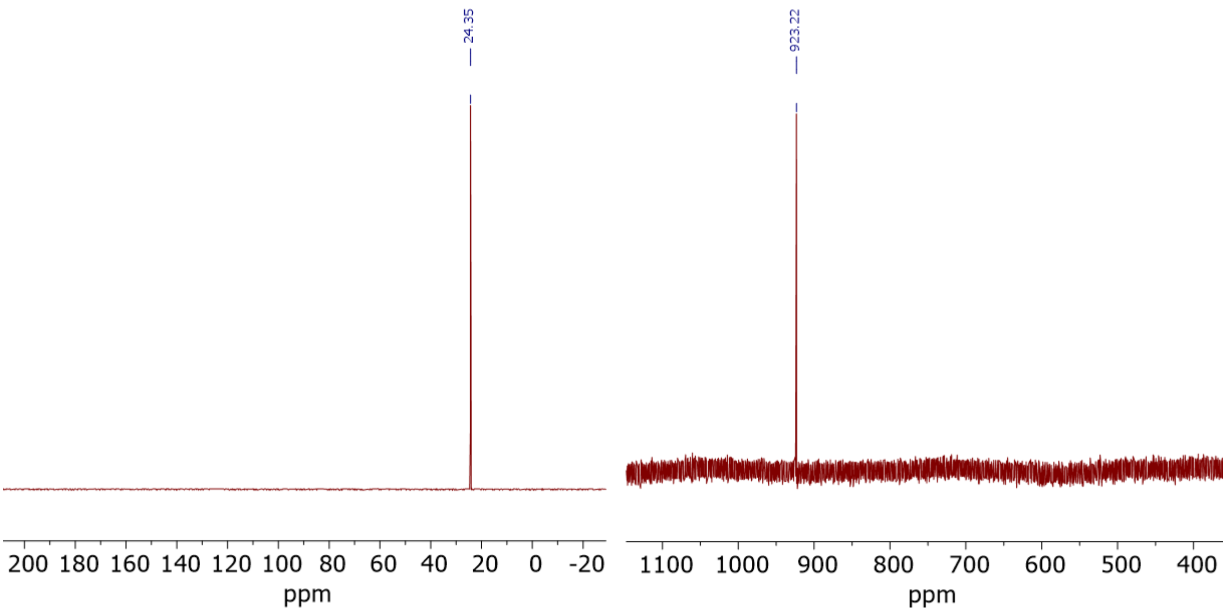

Figure S52. ^{31}P NMR (left panel) and ^{77}Se NMR (right panel) spectra of compound **1d**.

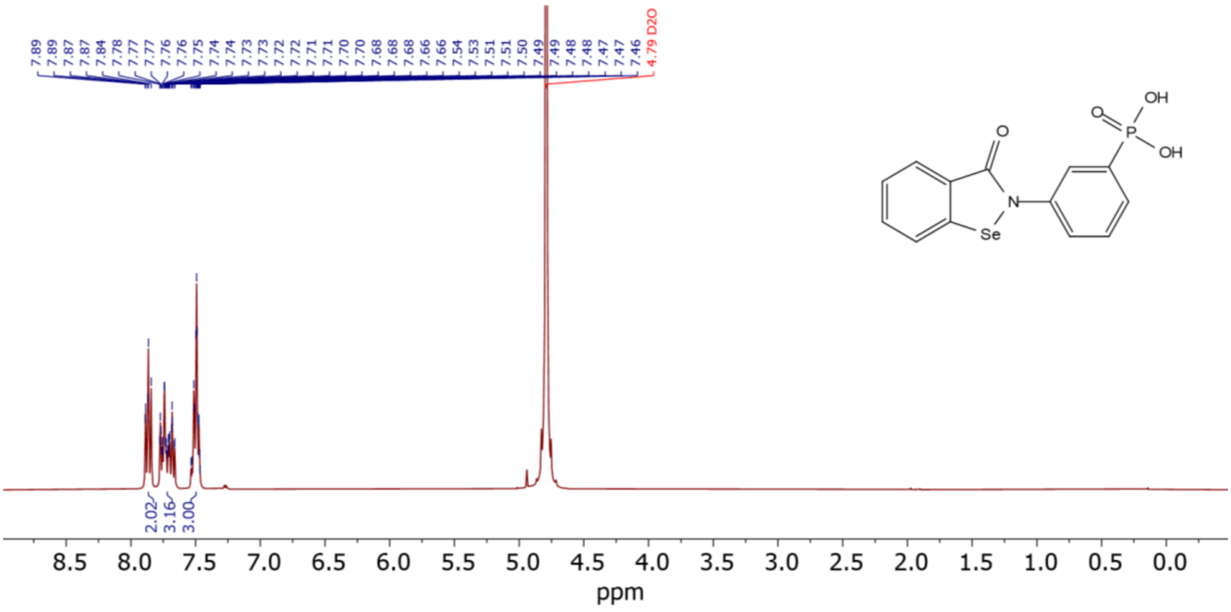

Figure S53. ^{1}H NMR spectrum of compound **1e**.

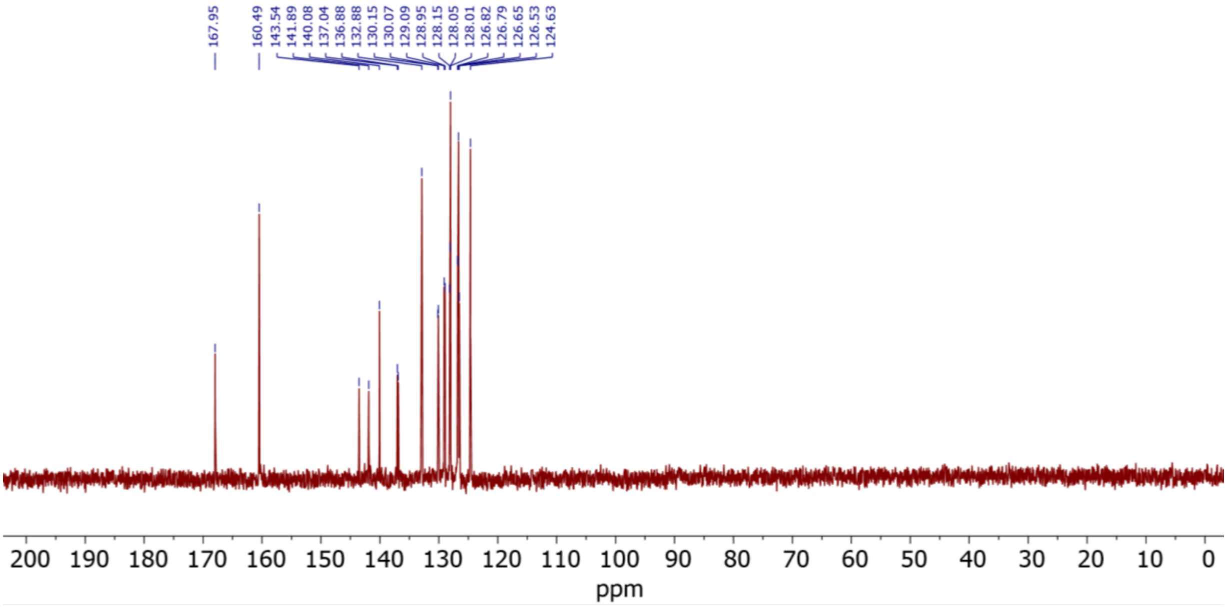

Figure S54. ^{13}C NMR spectrum of compound **1e**.

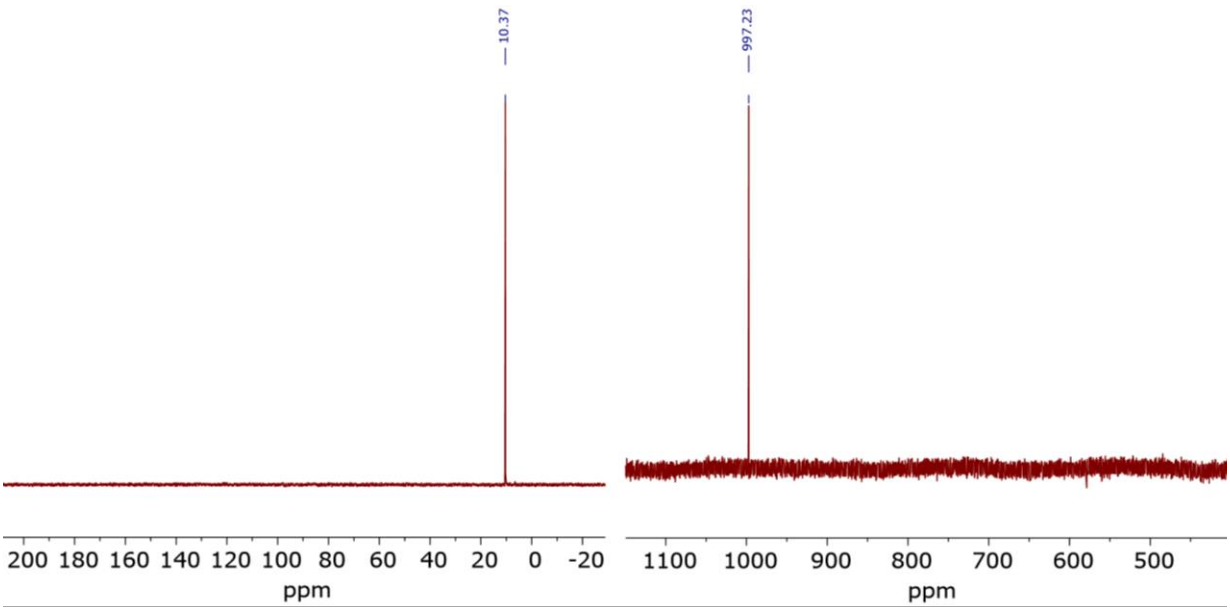

Figure S55. ^{31}P NMR (left panel) and ^{77}Se NMR (right panel) spectra of compound **1e**.

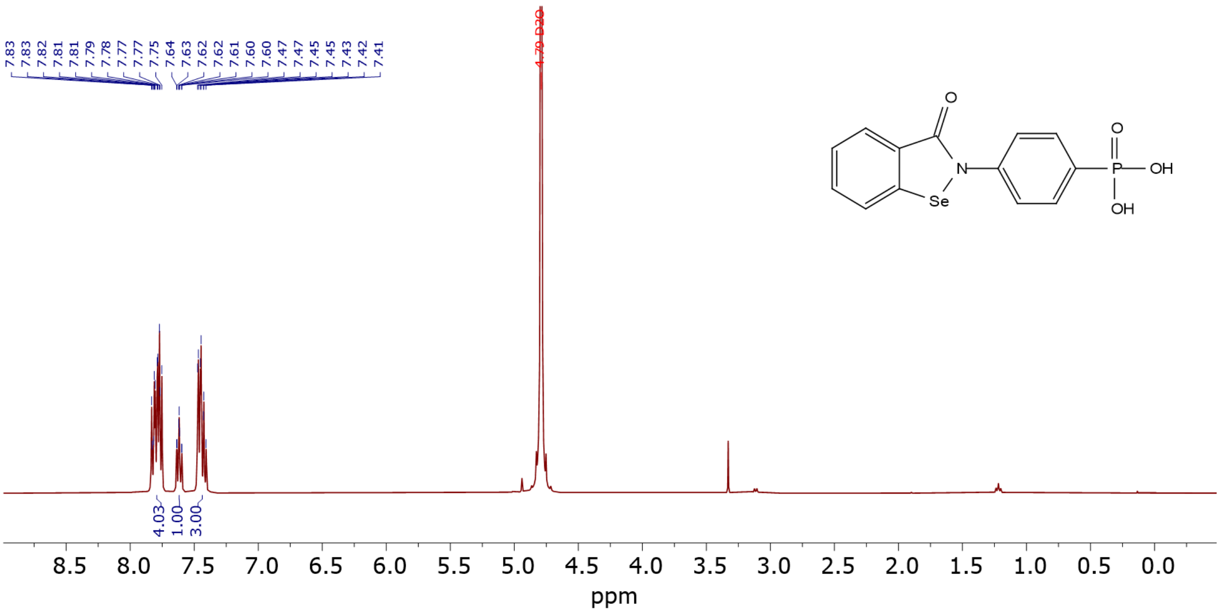

Figure S56. ^{1}H NMR spectrum of compound **1f**.

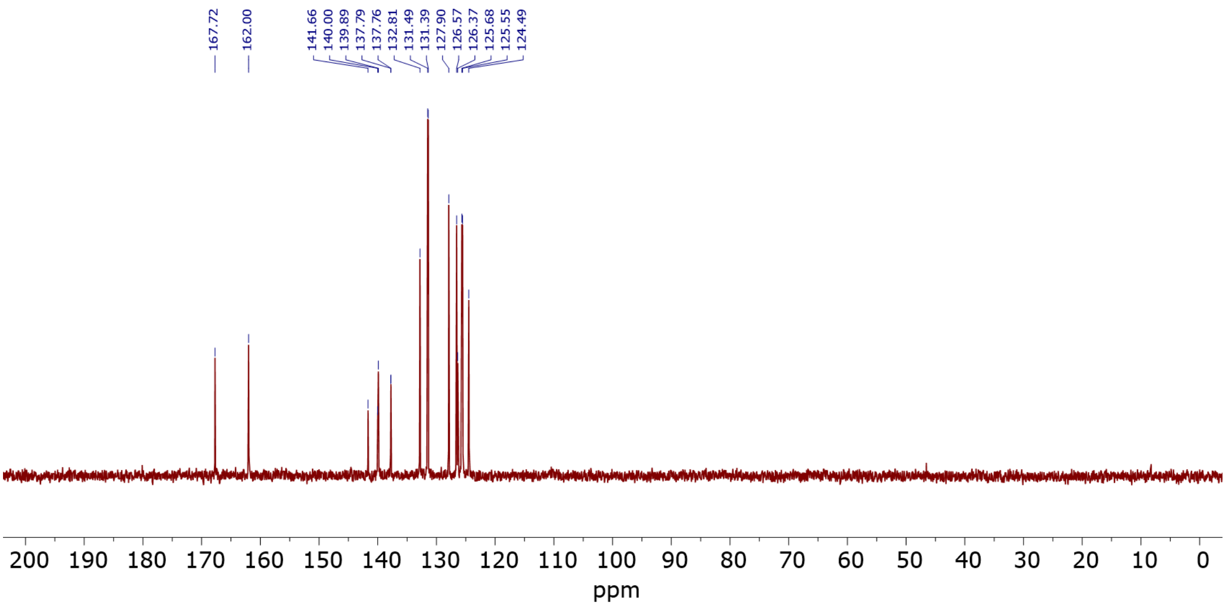

Figure S57. ^{13}C NMR spectrum of compound **1f**.

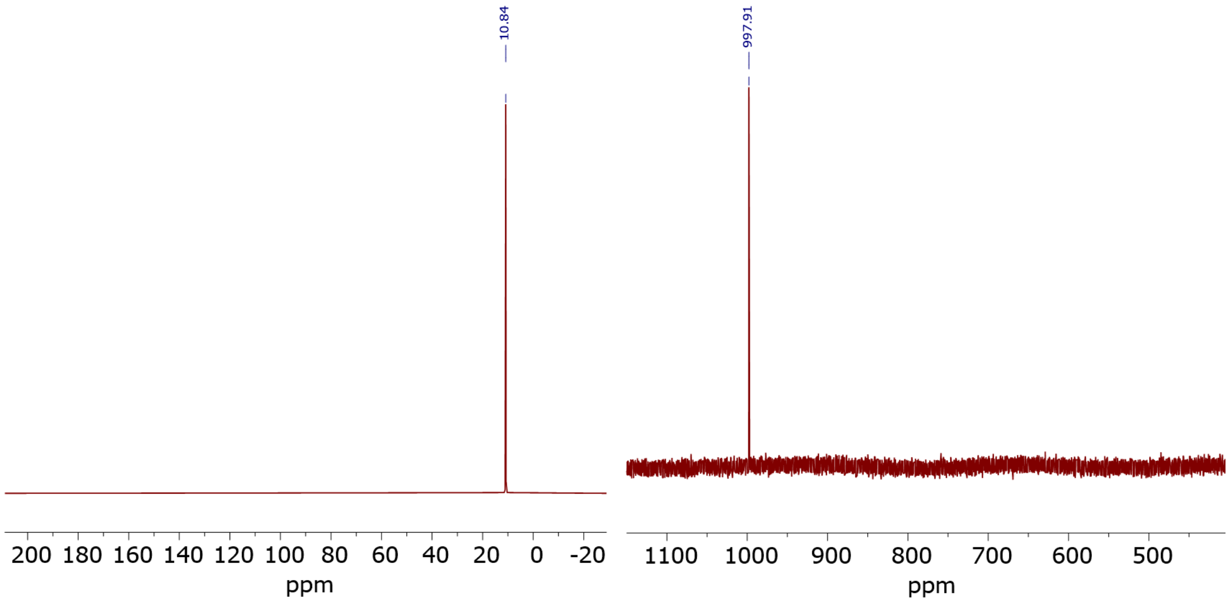

Figure S58. ^{31}P NMR (left panel) and ^{77}Se NMR (right panel) spectra of compound **1f**.

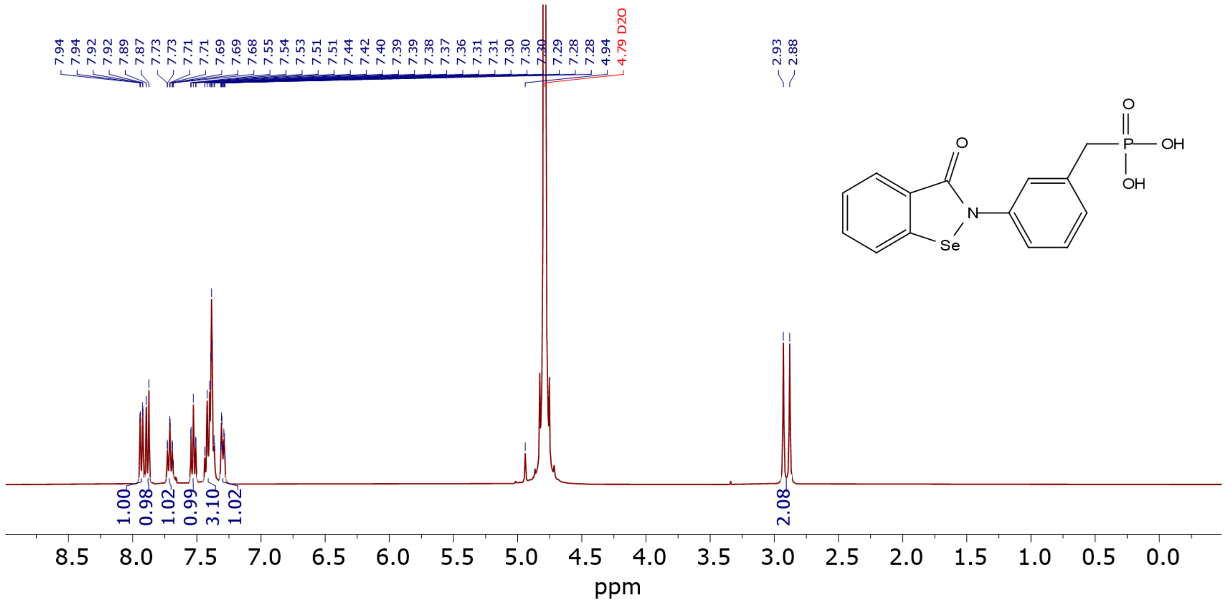

Figure S59. ^{1}H NMR spectrum of compound **1g**.

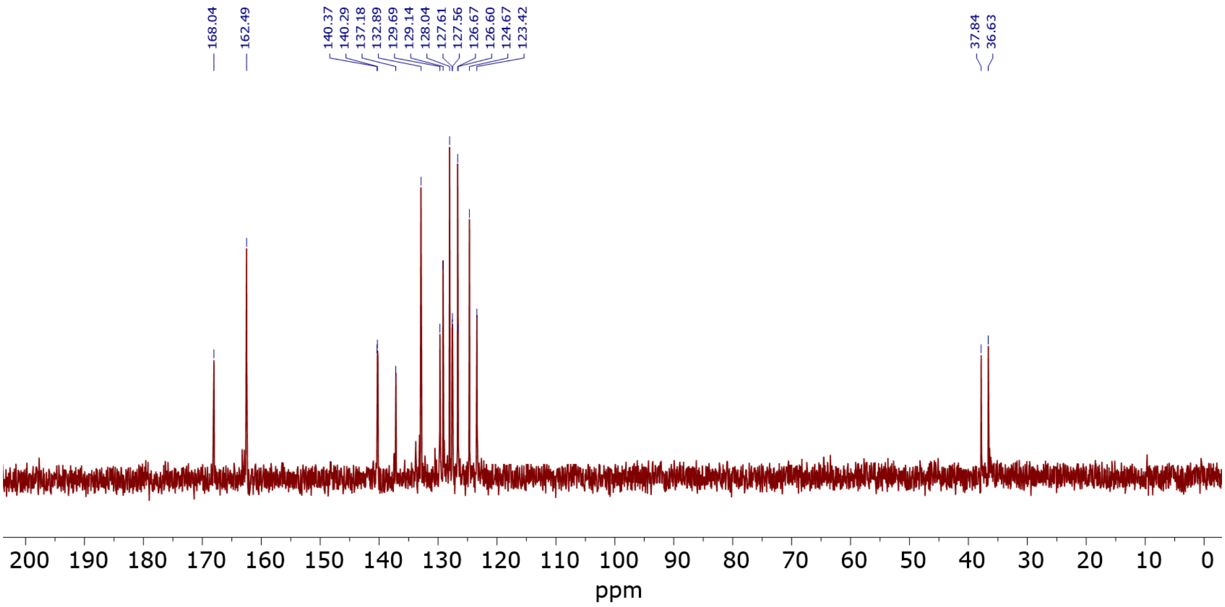

Figure S60. ^{13}C NMR spectrum of compound **1g**.

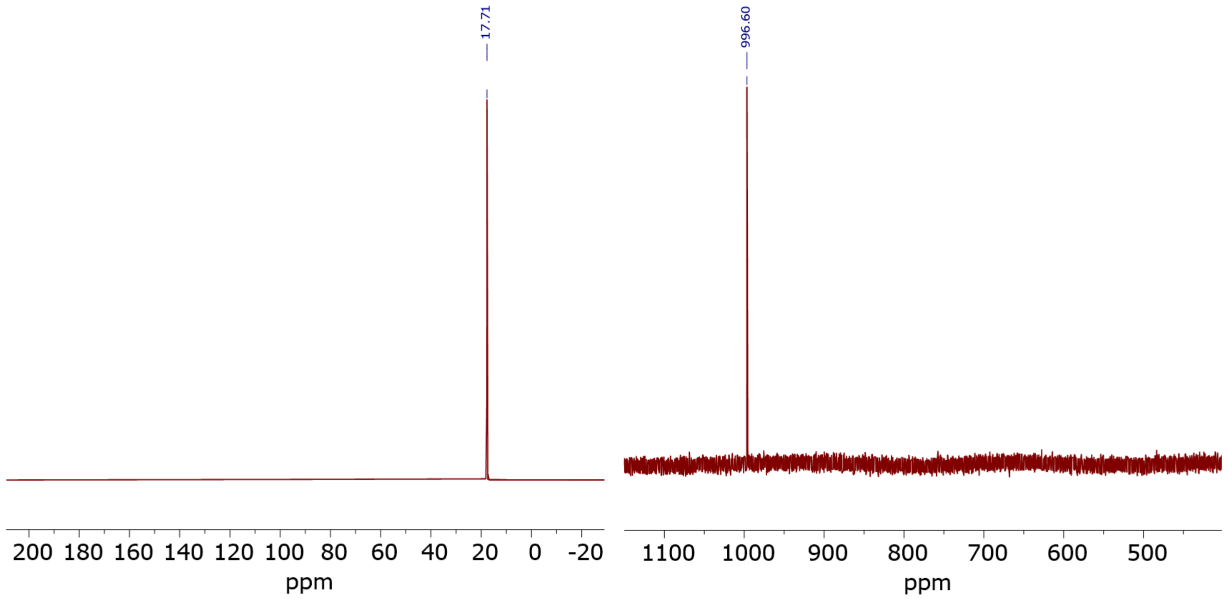

Figure S61. ^{31}P NMR (left panel) and ^{77}Se NMR (right panel) spectra of compound **1g**.

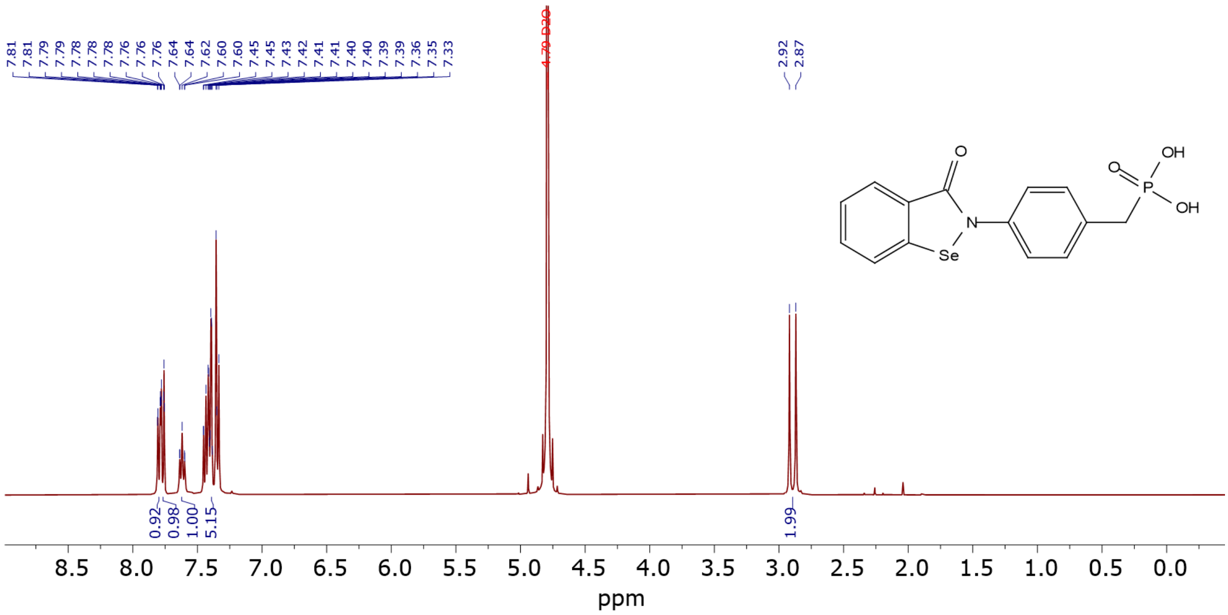

Figure S62. ^{1}H NMR spectrum of compound **1h**.

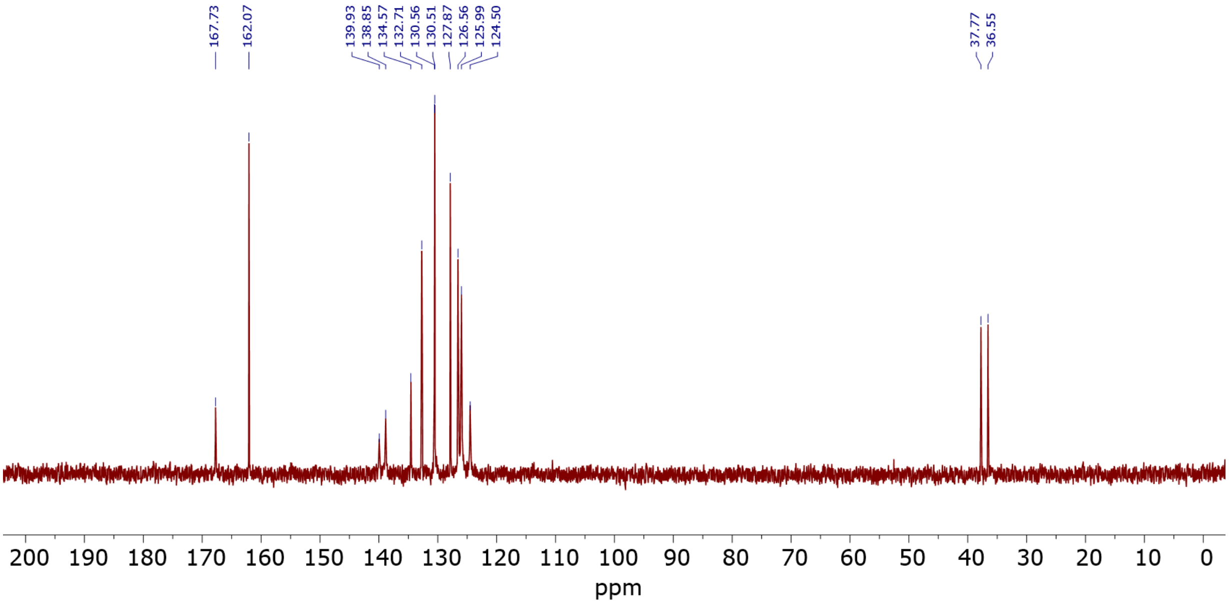

Figure S63. ^{13}C NMR spectrum of compound **1h**.

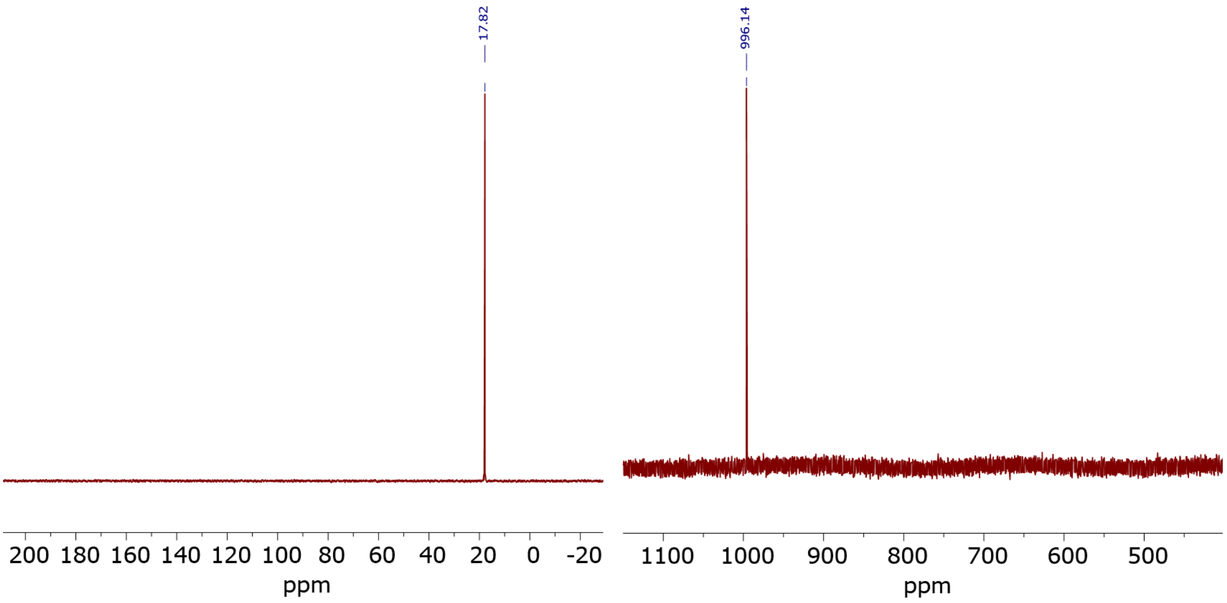

Figure S64. ^{31}P NMR (left panel) and ^{77}Se NMR (right panel) spectra of compound **1h**.

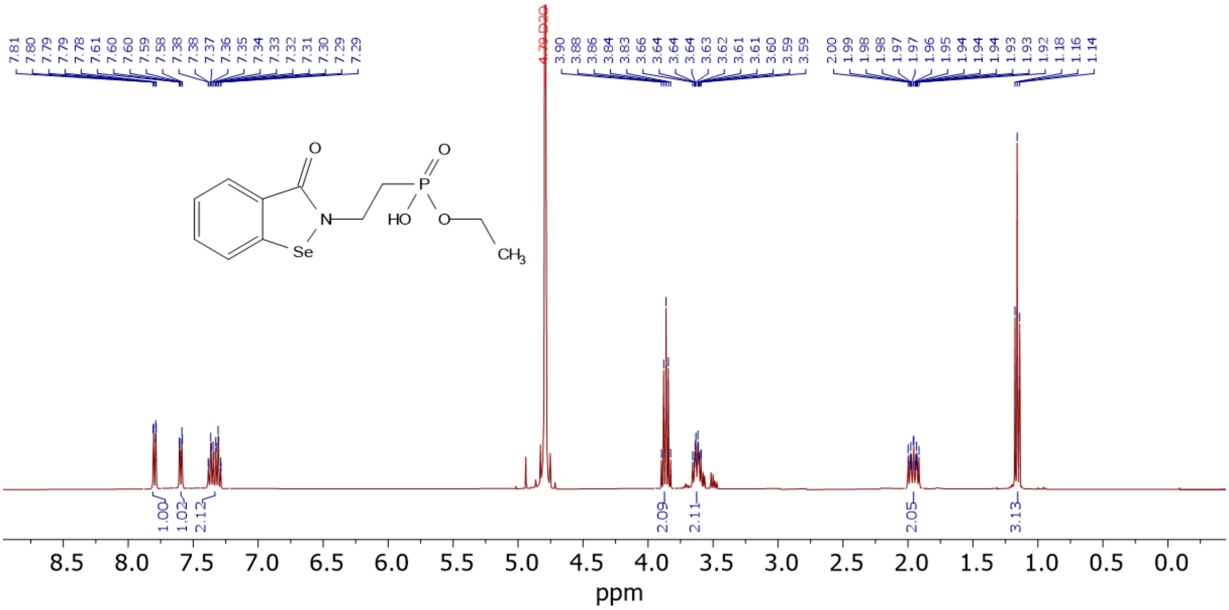

Figure 65. ^{1}H NMR spectrum of compound **15a**.

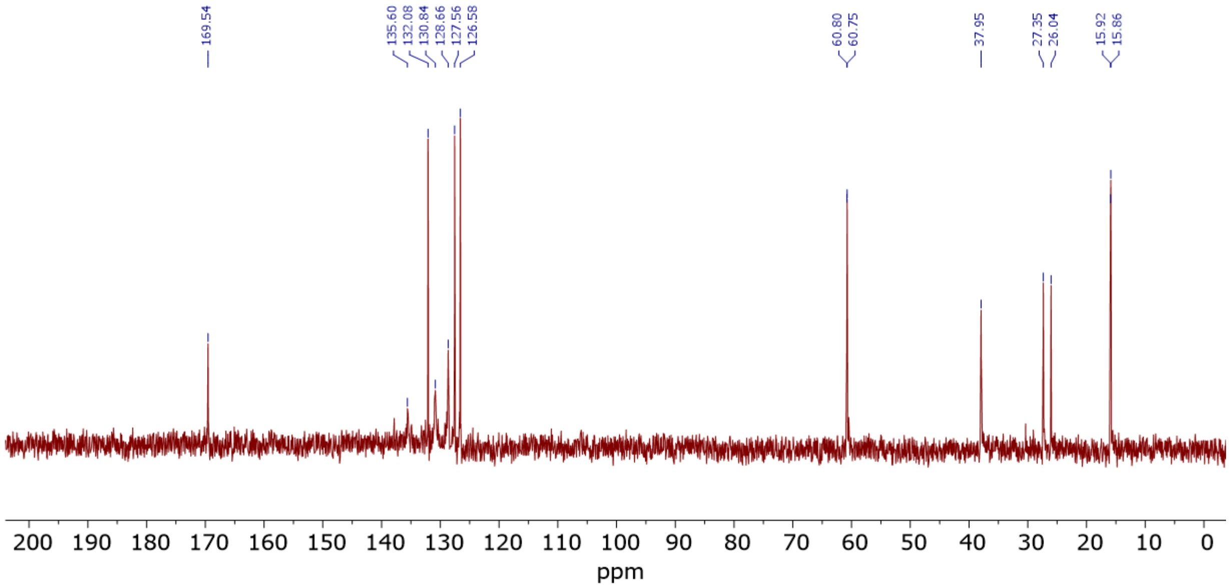

Figure 66. ^{13}C NMR spectrum of compound **15a**.

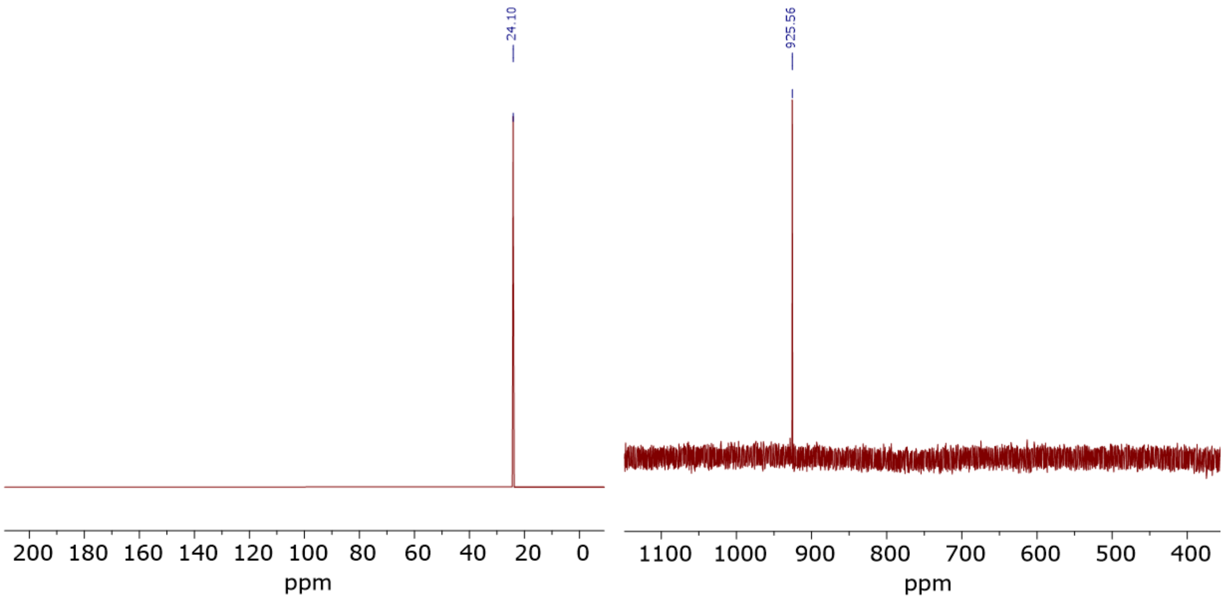

Figure 67. ^{31}P NMR (left panel) and ^{77}Se NMR (right panel) spectra of compound **13a**.

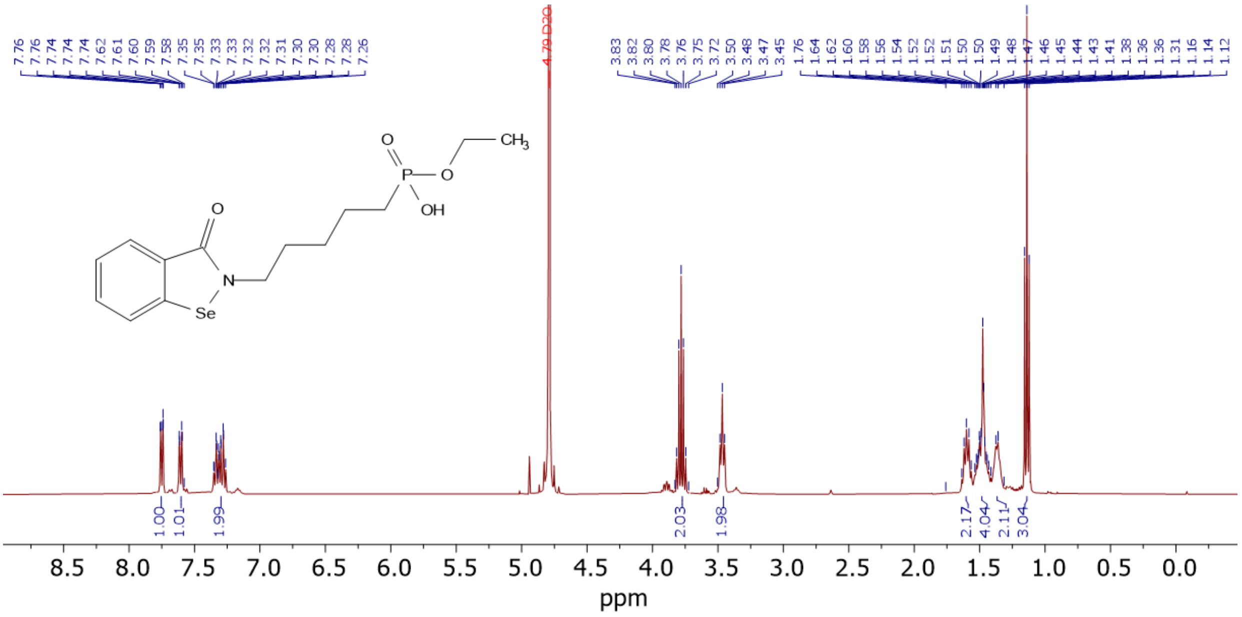

Figure 68. ^{1}H NMR spectrum of compound **15b**.

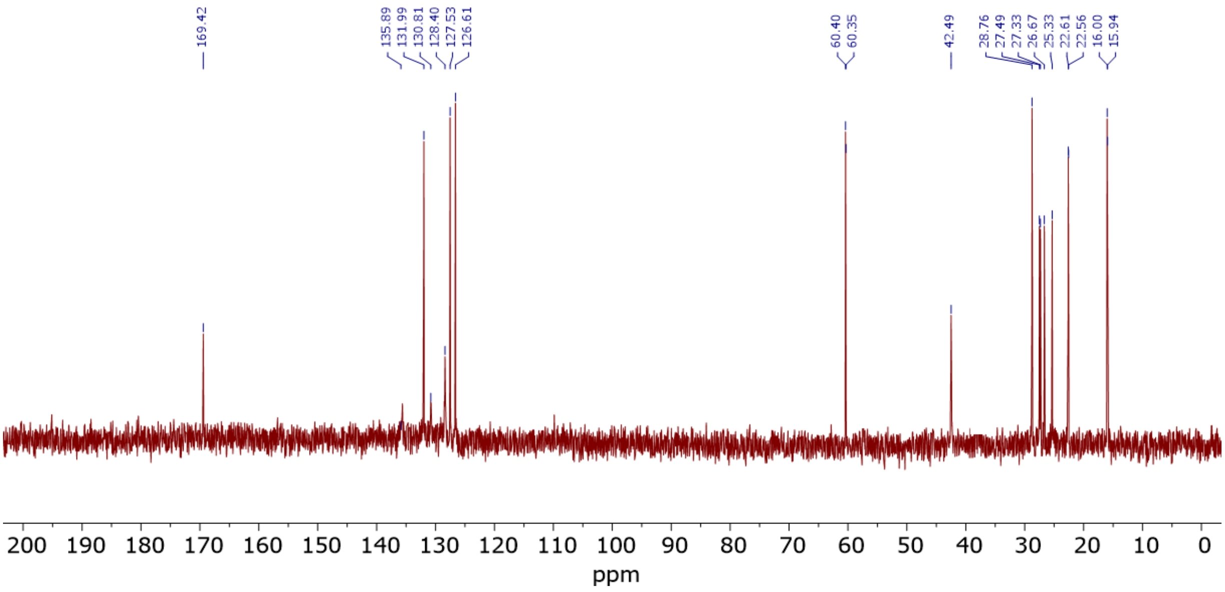

Figure 69. ^{13}C NMR spectrum of compound **15b**.

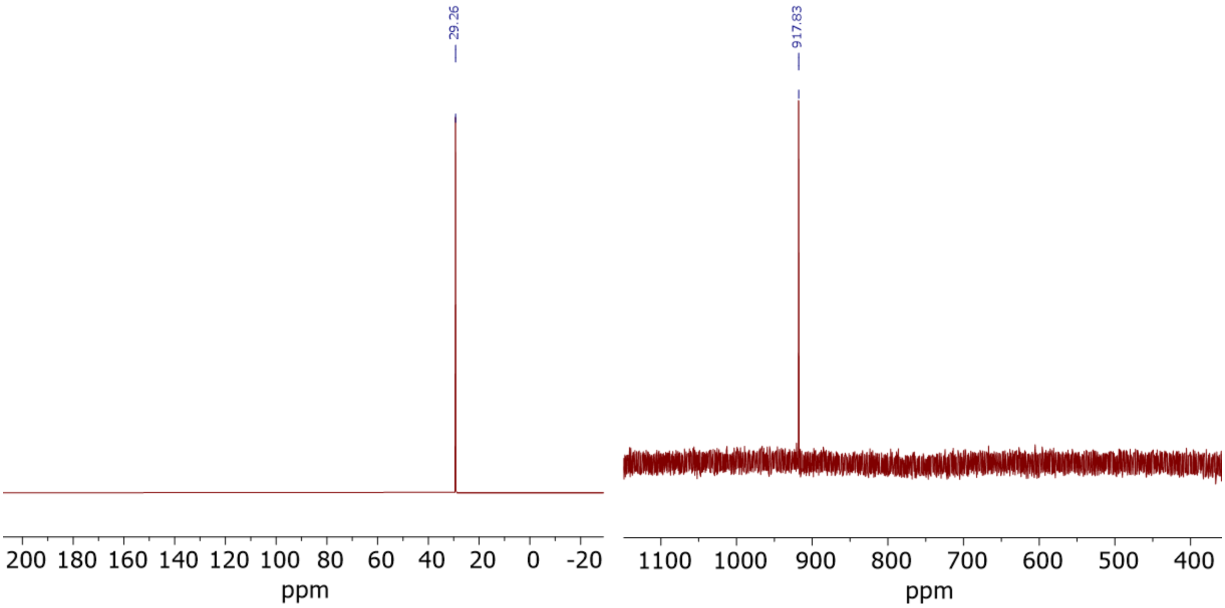

Figure 70. ^{31}P NMR (left panel) and ^{77}Se NMR (right panel) spectra of compound **15b**.

S7. Representative HPLC Analyses

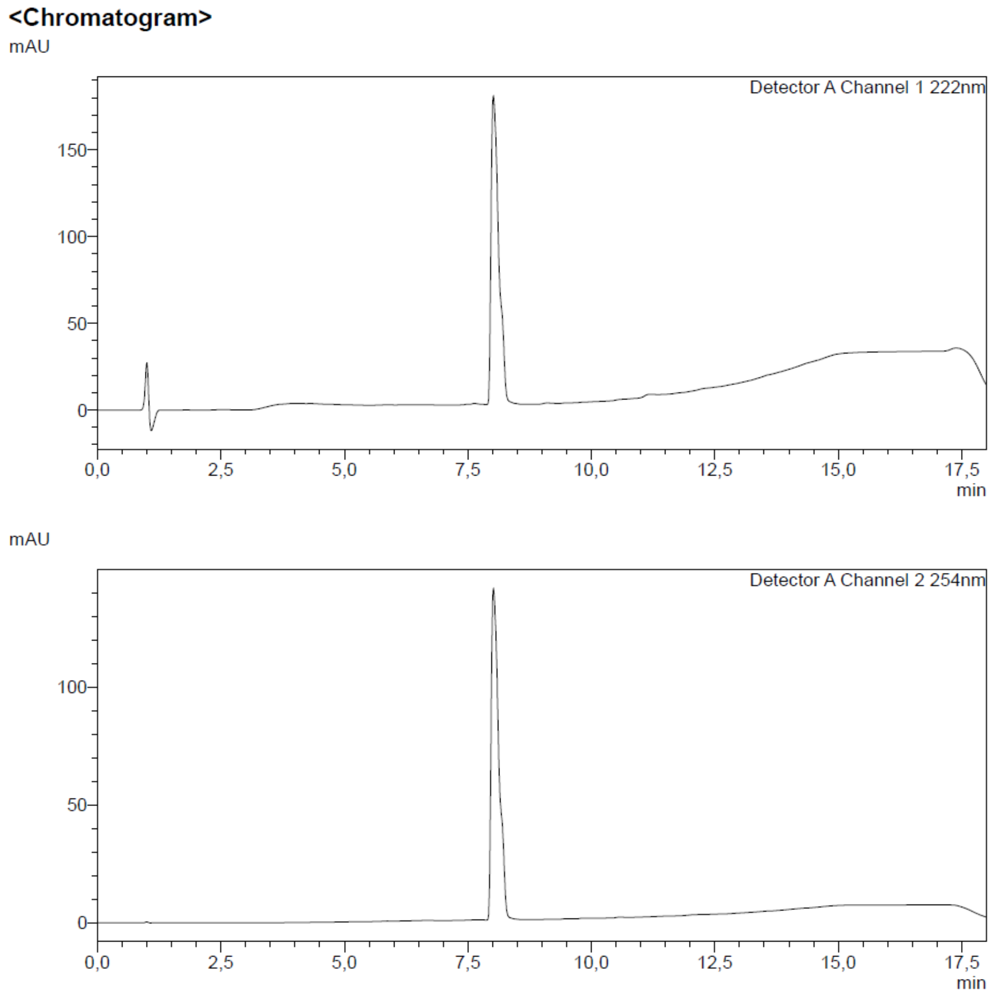

Figure S71. Analytical HPLC of compound **14f**.

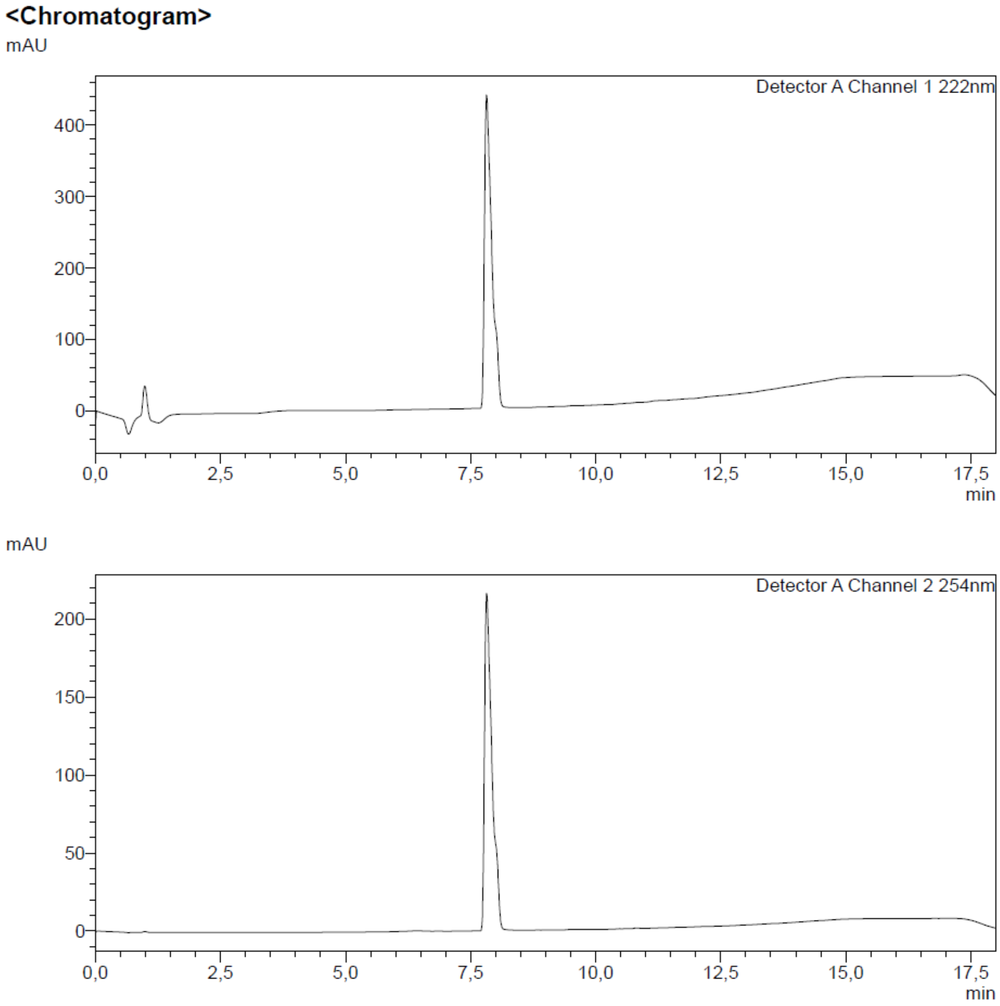

Figure S72. Analytical HPLC of compound **14g**.

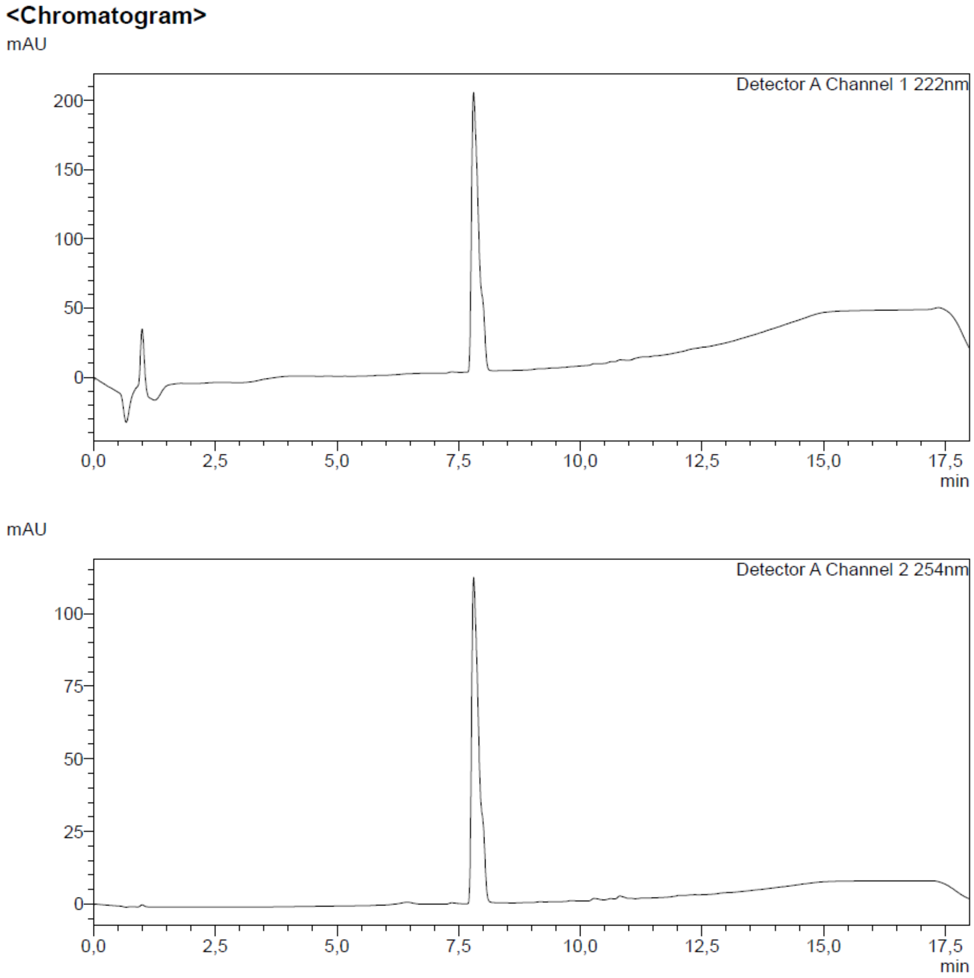

Figure S73. Analytical HPLC of compound **14h**.

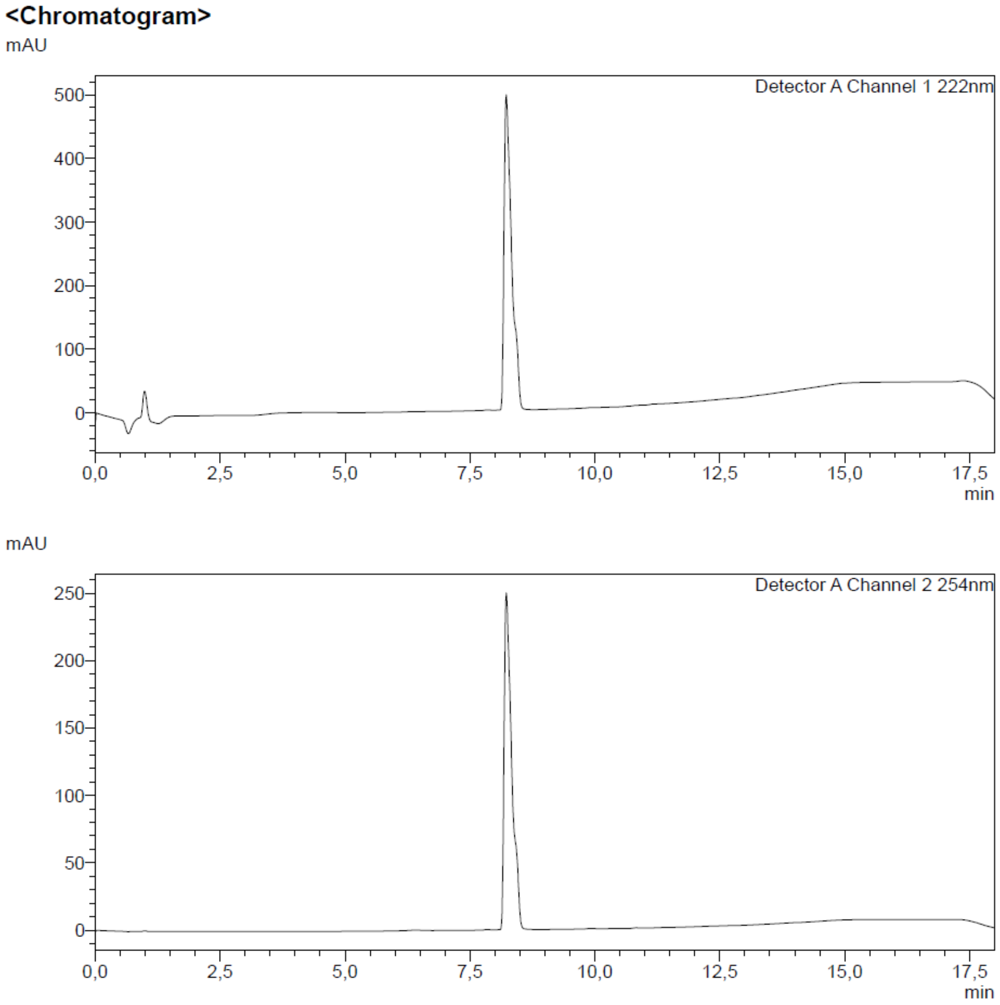

Figure S74. Analytical HPLC of compound **14i**.

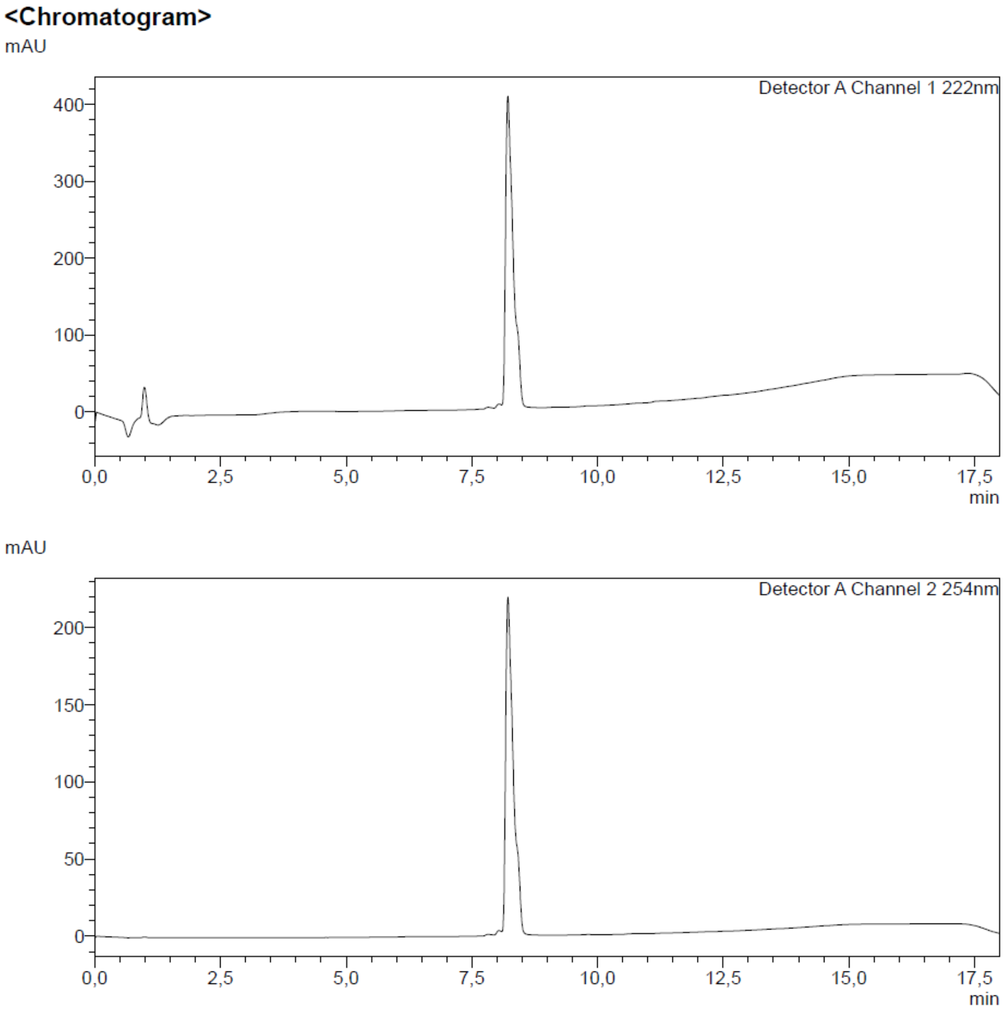

Figure S75. Analytical HPLC of compound **14j**.

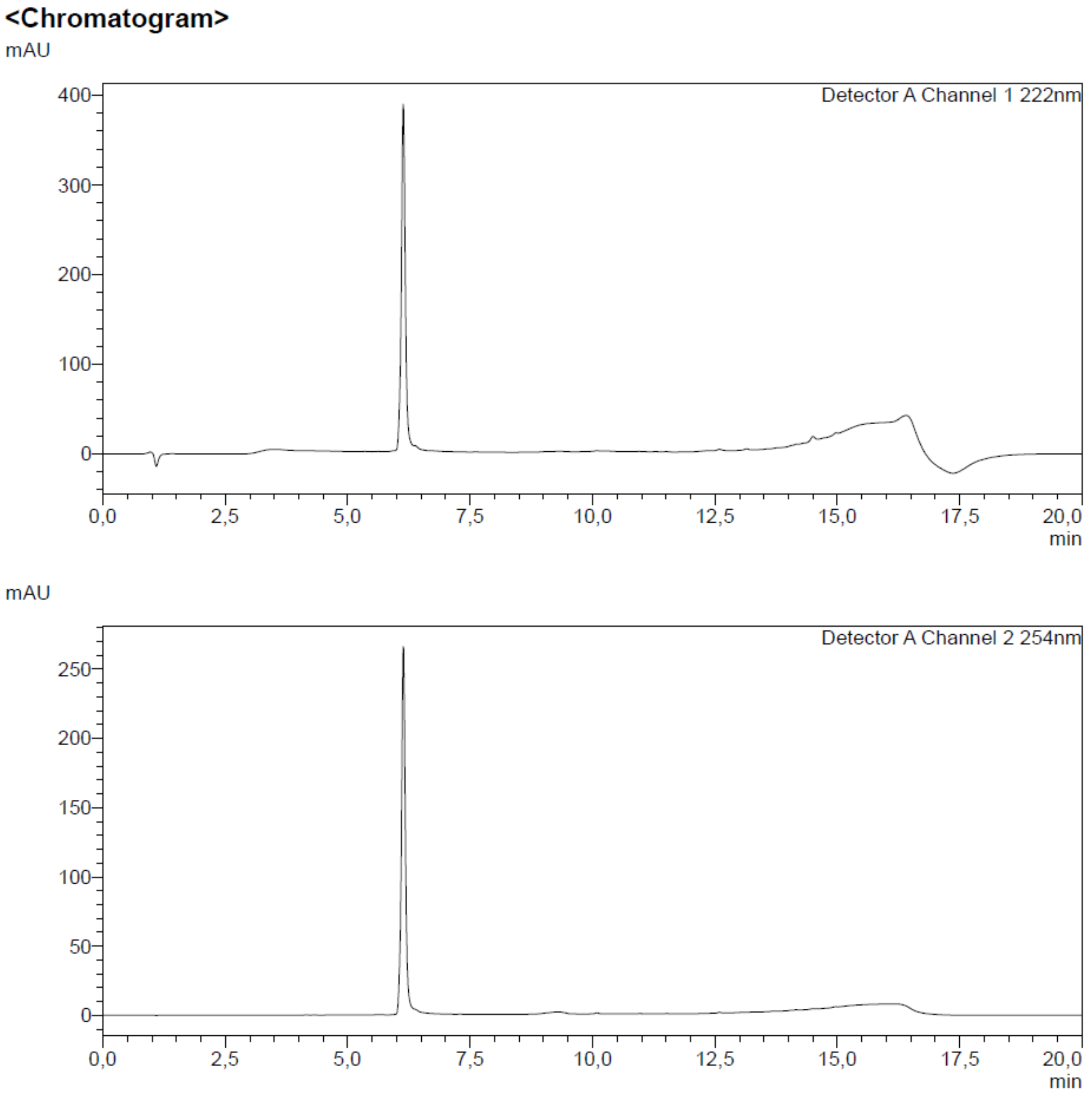

Figure S76. Analytical HPLC of compound **1b**.

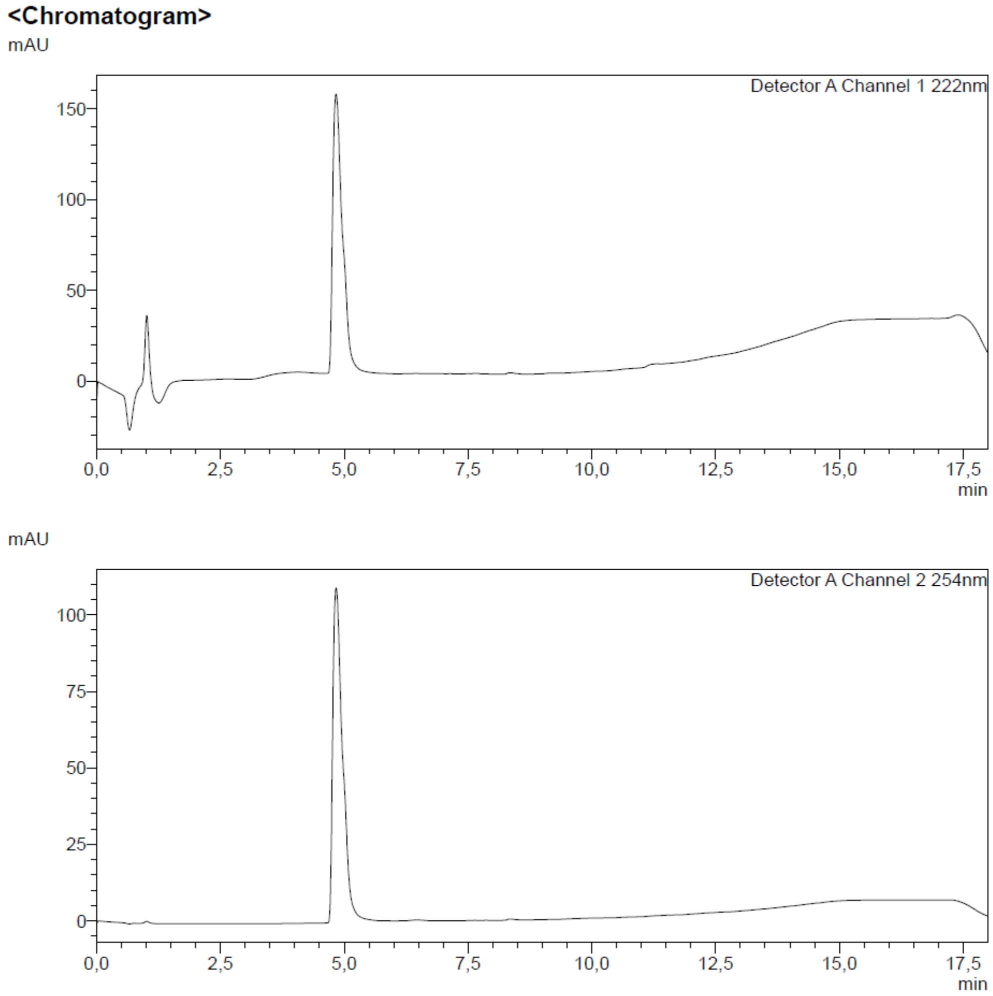

Figure S77. Analytical HPLC of compound **1d**.

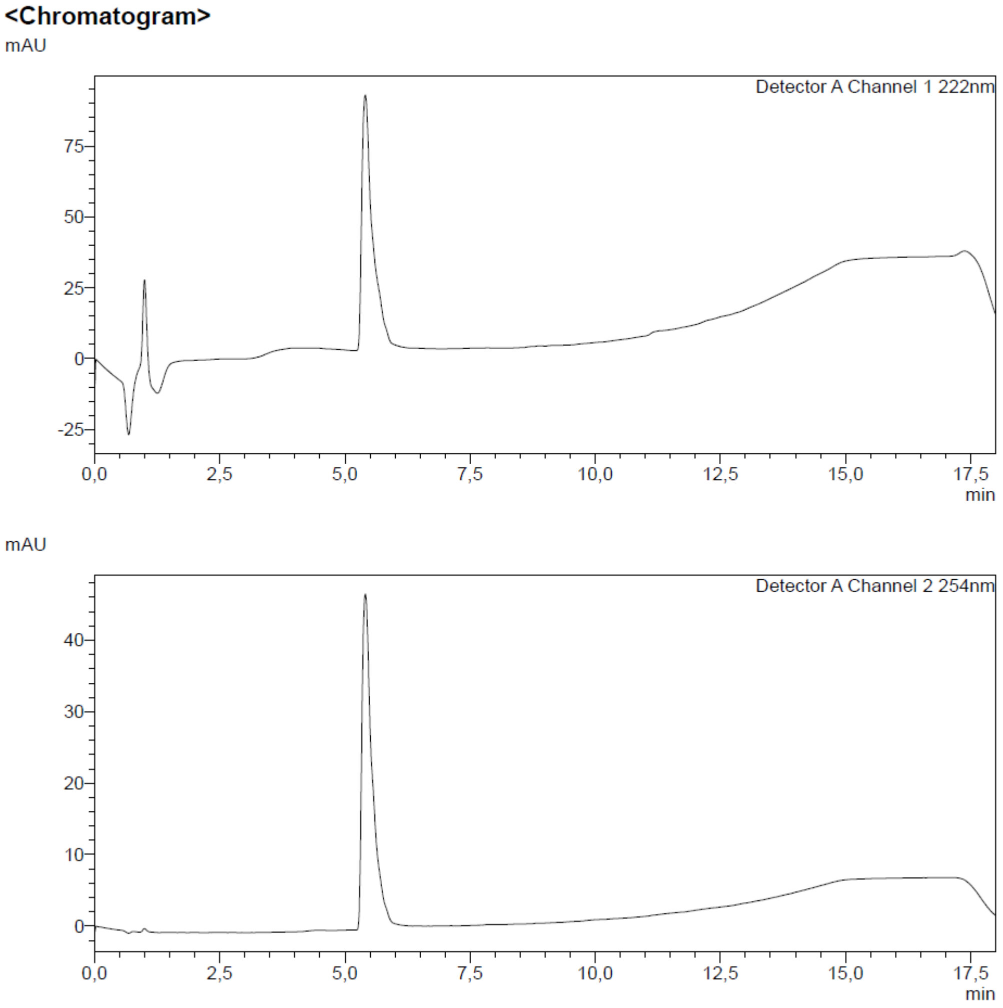

Figure S78. Analytical HPLC of compound **1g**.

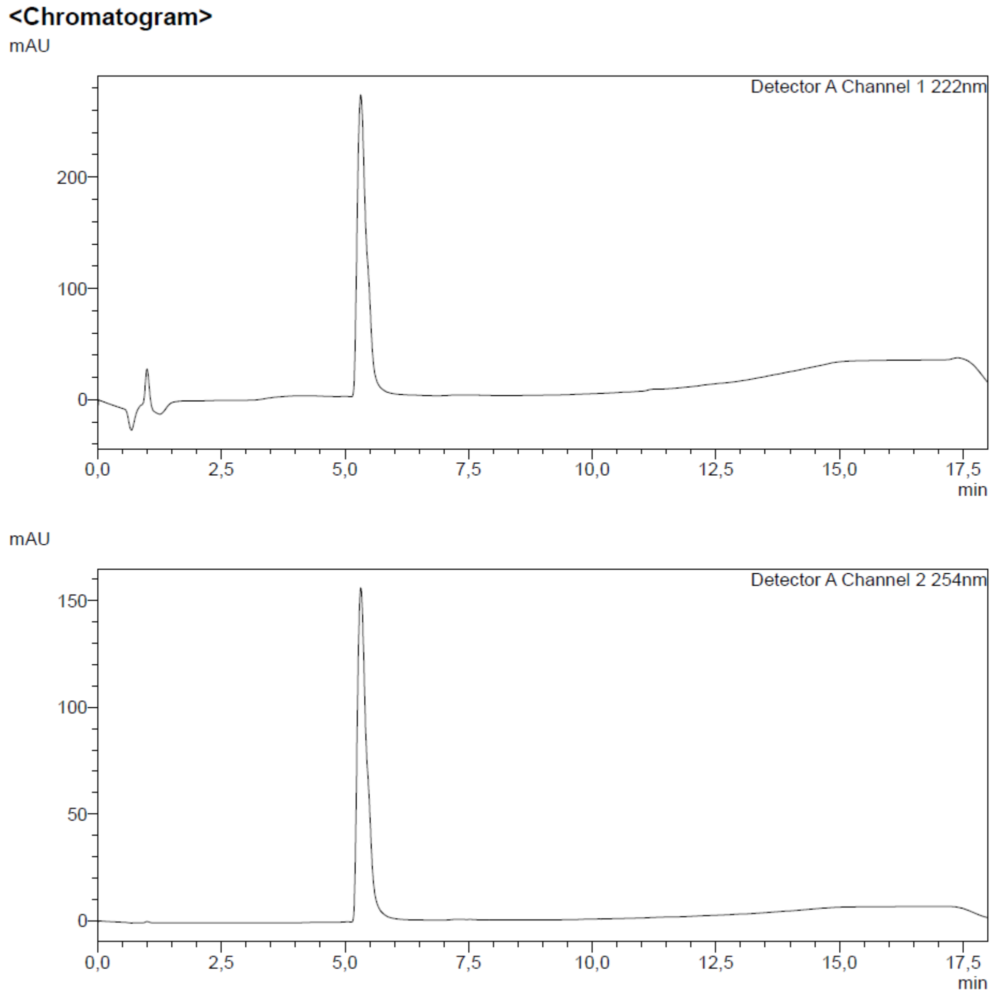

Figure S79. Analytical HPLC of compound **1h**.
